# Electronic medicine management systems in developing countries: A landscape review

**DOI:** 10.1002/bcp.70156

**Published:** 2025-07-01

**Authors:** Andrew Lambarth, Dalia Wainwright, Trisha Saha, Millicent Banks, Iona Minty, P.A.M.K. Abeywickrama, Reya V. Shah, Yogini Jani, Reecha Sofat

**Affiliations:** ^1^ Department of Clinical Pharmacology and Therapeutics City St George's, University of London London UK; ^2^ St George's University Hospitals NHS Foundation Trust London UK; ^3^ Oxford University Hospitals NHS Foundation Trust Oxford UK; ^4^ School of Medicine University College London London UK; ^5^ University Hospital Southampton NHS Foundation Trust Southampton UK; ^6^ Department of Infectious Diseases University College Hospital London UK; ^7^ Department of Surgery and Cancer Imperial College London London UK; ^8^ Post Graduate Institute of Medicine Sri Lanka; ^9^ School of Pharmacy University College London London UK; ^10^ Centre for Medicines Optimisation Research and Education UCLH NHS Foundation Trust London UK; ^11^ BHF Data Science Centre HDRUK London UK; ^12^ Department of Pharmacology and Therapeutics University of Liverpool Liverpool UK

**Keywords:** developing countries, digital, electronic, low‐ and middle‐income countries, medicine management, review

## Abstract

Medicines are a major global health expense. However, suboptimal use increases costs and causes patient harm. One way to reduce costs and increase safe, efficient medicines use is with electronic medicines management systems (EMMS). They allow easy capture of routine health data which can facilitate research, service planning and reimbursement processes. There are various barriers to healthcare digitization in developing countries (DCs), although some have overcome these. We sought to understand the landscape of EMMS use in DCs. We systematically searched six bibliographic databases from inception to 23 October 2024 for studies reporting the implementation and/or use of EMMS in countries with lower than ‘very high’ Human Development Index (HDI). We qualitatively and quantitatively summarized data on geographic location, healthcare setting and system functionality. We created an interactive map illustrating spatial and temporal trends in EMMS use. A total of 314 records described the use of EMMS in 45 DCs, 206 of which described coexistence/integration of other health data (e.g., electronic health records [EHR]). Predominantly, EMMS were for prescribing (*n* = 264) or dispensing (*n* = 66), implemented in secondary care settings and operated locally rather than regionally or nationally. Common EMMS use‐cases included adherence monitoring in human immunodeficiency virus (HIV) and tuberculosis treatment. Our findings highlight both widespread EMMS adoption—commonly in the context of a broader EHR—and persistent gaps in implementation. These insights could be used by policymakers and healthcare leaders to guide strategy and funding decisions. Existing systems could be leveraged for service planning, healthcare delivery and optimizing medicine use. Where EMMS are not yet in use, our findings provide a roadmap for stakeholders to identify and emulate successful implementations in similar healthcare settings. Expanding the interoperability and scale of EMMS could further enable transformative digital technologies, increasing efficiencies and coverage, and ultimately improving patient outcomes.

## 1 INTRODUCTION

Global expenditure on medicines continues to rise, with lower‐income countries tending to allocate a greater share of health expenditure—sometimes up to 50%—to pharmaceutical products.^1^ Despite these substantial investments, use of medicines often falls short of optimal standards, and this is a major cause of preventable patient harm.^2^ A 2023 World Health Organization (WHO) bulletin underscored mounting evidence of medication overuse in low‐ and middle‐income countries, which may contribute to increased spending.^3^, ^4^ However, it is important to note that underuse of effective therapies is also a critical source of inefficiency and deleterious health consequences.

Point‐of‐care electronic medicine management systems (EMMS)—electronic tools or software designed to facilitate any or all stages of the medicines management cycle—have the potential to improve productivity, reduce medication errors and promote cost‐effective therapy.^5^, ^6^, ^7^, ^8^, ^9^ These systems can be deployed across a spectrum of healthcare settings and at various stages of the medicine management process, encompassing prescribing, dispensing, administration, adherence monitoring and stewardship (e.g., antimicrobials). Generation of routine healthcare data not only enables pharmacovigilance and pharmacoepidemiology, but also with transformative digital technologies including artificial intelligence, digitization could represent a real step change for DC health economies.

In many high‐income nations both EMMS and more comprehensive electronic health records systems (EHR) have become commonplace, serving diverse healthcare settings.^10^, ^11^ Large electronic databases of health records, such as the Clinical Practice Research Datalink (CPRD) in the UK,^12^ Japan Medical Data Center (JMDC) Claim and Medical Data Vision (MDV) Databases in Japan,^13^ and the National Institutes of Health (NIH) Collaboratory Distributed Research Network in the USA,^14^ incorporate routinely collected medicines data and support extensive population health research. However, there are various practical and social barriers to the implementation of EMMS in DCs, which may influence uptake. Barriers to digitization are a source of health inequality, and the lack of point‐of‐care systems and electronic health data impedes both clinicians' and researchers' ability to optimally serve patients.

Here, we present a systematic review describing the recent landscape of EMMS usage in DCs. By extension, we also explore the location and setting of existing datasets, which could be harnessed for pharmacoepidemiological studies in international and underserved populations, including those who may often also be underrepresented in medical research.

## 2 METHODS

### 2.1 Search strategy

We carried out a systematic review, searching six literature databases (MEDLINE, EMBASE, CENTRAL, CINAHL, IPA, Web of Science) from inception to 28 May 2021. These searches were updated on 23 October 2024. Our search terms included MeSH and free text terms adapted from reviews on similar topics,^15^, ^16^ and built around the two key concepts of EMMS and DCs. We screened the references of relevant review articles for additional records not identified by our searches. Full search terms are provided in Supplementary Material, Section 3.

### 2.2 Screening

To be eligible, records were required to describe the use of an EMMS at the point‐of‐care, and/or the existence of electronic medicines usage data on a patient or prescription level (i.e., not a stock management level which could be affected by factors other than actual consumption). Reference to electronic medical records and related terms were only deemed eligible if there was also explicit mention of medicines information existing within these records. Private health insurance databases were not eligible. Systems were required to have been deployed in a DC, which we defined as any country not classed as ‘very high’ Human Development Index (HDI) in the 2020 United Nations Development Programme Human Development Report.^17^ A list of eligible countries is provided in the Supplementary Material, Table S3. Articles not in the English language were translated using Google Translate, but were excluded if sections relating to the electronic system of interest were not sufficiently clear and comprehensible to allow the characteristics of the system they described to be interpreted. After the removal of duplicates, for the original searches performed in 2021, three investigators independently screened records for relevance and eligibility, with disagreements settled by consensus after full‐text review.

### 2.3 Data extraction

Four investigators independently populated a data extraction table with relevant information from eligible articles. A single investigator (A.L.) reviewed all data and full‐text articles to ensure consistency in reporting, with identified discrepancies resolved by consensus. Where reported, data extracted included: the location (continent, country and specific area), healthcare setting (private healthcare, community pharmacy, primary care or secondary/tertiary care, number of sites), and system characteristics (stage of medicines management at which it was used, whether it is involved in the reimbursement process, and whether a unique patient identification number is used). We inferred the organizational level at which a system was operated or implemented, namely: local, regional, organizational/network and national. Where one or more sites were described without explicit reference to a common health authority, organization or region, this was assumed to be local.

### 2.4 Updated searches

For the updated searches performed in 2024, a single investigator (A.L.) screened and extracted data for all records, and a second investigator (M.A.) independently screened and extracted 20% to ensure consistency.

We summarized the characteristics of included articles in narrative and tabular format, and created figures using R version 4.1.2,^18^ and maps,^19^ sf^20^ and ggiraph packages.^21^ We implemented an interactive web app using shiny.^22^ For the purposes of this descriptive review, we did not deem it appropriate to assess risk of bias or evidence quality. We referred to PRISMA guidance when writing the manuscript^23^; a checklist is provided in the Supplementary Material Section 4.

## 3 RESULTS

**FIGURE 1:**
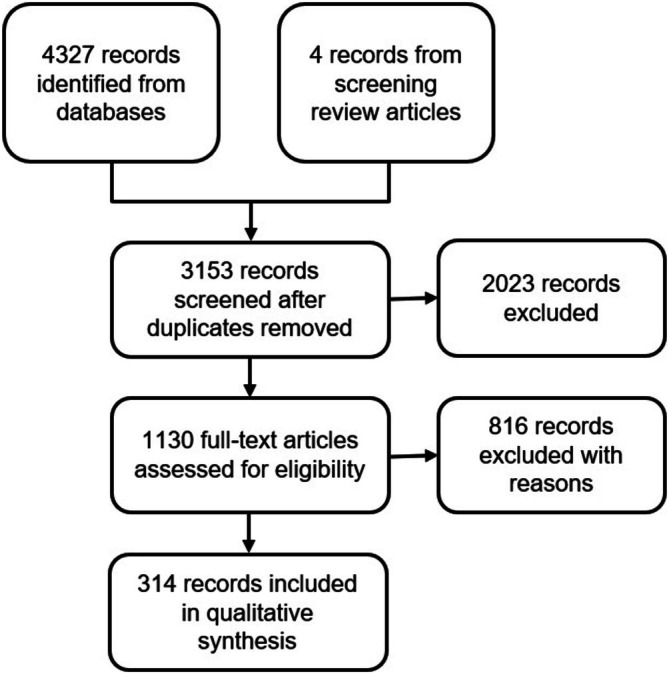
PRISMA flow chart.

Our searches identified 4327 records, and a further four eligible records were identified by manually screening the references of related articles. After duplicate removal and screening, 314 of these were found to be eligible for inclusion (Figure 1; Supplementary Material Section 2; Supplementary Table S1). A brief description of the aims and characteristics of each study is provided in Supplementary Table S2.

### 3.1 Geographical distribution

EMMS were reported in 45 different countries across five continents. Twenty of these were countries categorized as having a ‘high’ HDI, while 12 were categorized as ‘medium’, and 13 ‘low’. However, countries with a higher HDI had a higher average number of records each (median number of publications for high, medium and low HDI, respectively: 3.5, 3, 1). A total of 126 (40.1%) of 314 included records described systems in place in either Brazil or China, which both have a ‘high’ HDI. Most records (204 of 314; 65.0%) described systems that were operated on a local level, while 25 (8.0%) described regional systems, 51 (16.2%) were national, and 33 (10.5%) were implemented by an affiliated organization or network of sites.

**FIGURE 2:**
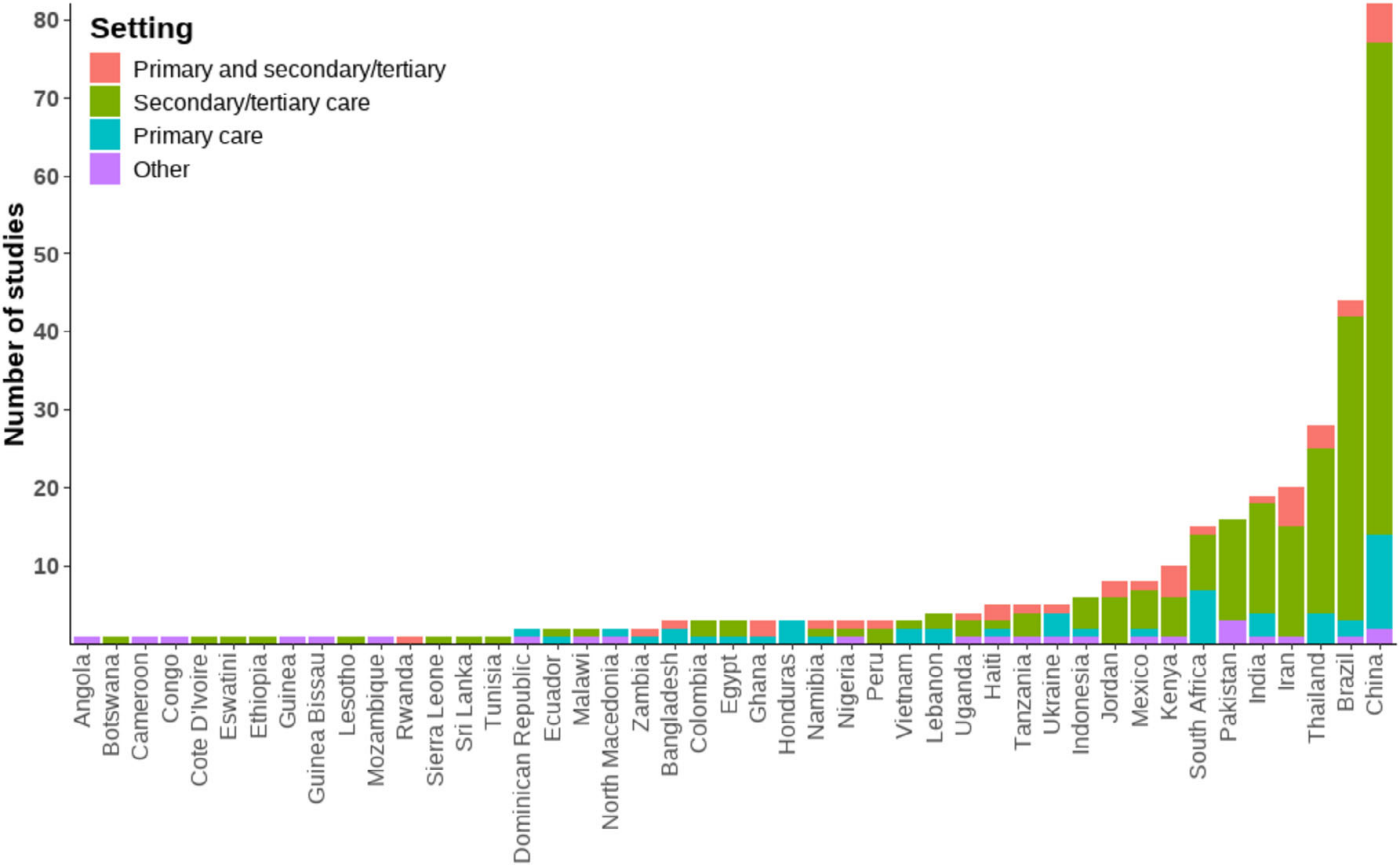
Distribution of included studies by country and setting. N.B. articles reporting a system used in multiple countries are counted once for each country.

**FIGURE 3:**
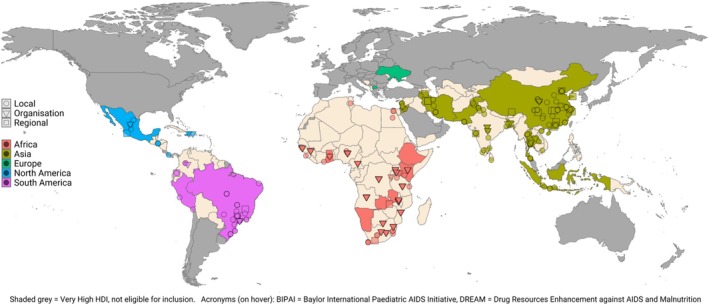
A map demonstrating the geographic distribution of the systems described in included articles. Grey = Not eligible (e.g. developed country, no HDI data); ○ = Local EMMS; □ = Regional EMMS; ∇ = EMMS used in an associated network of sites; Whole country shaded = National system. Interactive version available at https://alambarth.shinyapps.io/emms_developing_countries_app/

Figure 2 shows a breakdown of the included records by country and implementation setting. Figure 3 is a map summarizing the geographic distribution and characteristics of all included studies. Visual inspection of map plots revealed a notable absence of systems reported in parts of North Africa, Central Asia and South‐East Asia. There was also an apparent clustering of local, regional and network‐level systems within certain parts of some countries, generally in more urbanized areas (e.g., eastern China and south‐eastern Brazil).

### 3.2 Setting

The most common health setting observed was secondary or tertiary care, with 247 of 314 (78.7%) records reporting systems in place in a hospital or other specialist outpatient setting. Primary care clinical settings were reported in 76 (24.2%), and community pharmacies in 17 (5.4%) of the included records. Thirty‐five (11.1%) described a system with cross‐sector implementation (in both a primary and secondary/tertiary care setting).

Thirty (9.6%) records described EMMS that were implemented in healthcare settings which were at least partly private or for profit. Of these 18 were in ‘high’ HDI countries, 12 were in ‘medium’, and none were in ‘low’ HDI countries. While data on whether systems were open‐source was not formally collected, open‐source systems appeared to be far more commonly used in lower HDI settings. Many articles mentioned that the setting was ‘public’ or ‘government‐run’, but others did not contain sufficient detail to infer the facilities'/settings' funding sources or structures.

Many articles reported EMMS implemented for specific use‐cases. The most common example was for the provision of care for people living with human immunodeficiency virus (PLHIV). Sixteen unique articles described a system implemented for this use‐case. The next most frequent were tuberculosis (10), chemotherapy (4) and antimicrobial stewardship (4). General (4) and neonatal (3) intensive care settings were also relatively common.

### 3.3 System functionality

Most records—264 out of 314 (84.1%)—described electronic systems which had prescribing data or functionality. While most of these systems (160; 51.0%) constituted some form of electronic prescribing (e‐prescribing), the other 104 (33%) records did not describe an e‐prescribing system used at the point‐of‐care. For example, in some cases, paper prescriptions were later transcribed so that there were electronic records of prescriptions, but there was no order entry capability. Similarly, while 66 (21.0%) articles reported systems with dispensing data or functionality, only 29 of these were explicitly described as being used at the point‐of‐care.

**FIGURE 4:**
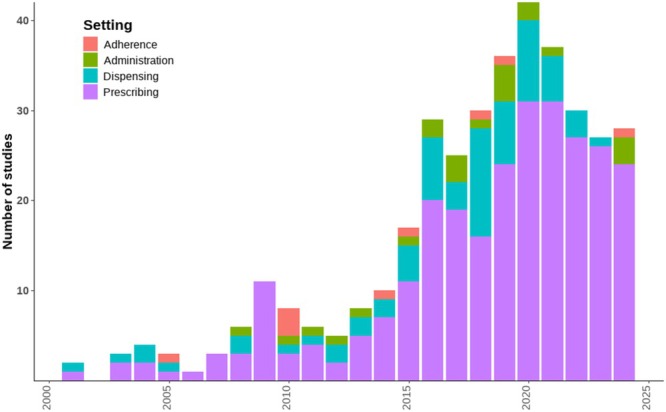
Number of included studies over time, coloured by medicine management stage. N.B. articles reporting a system used at more than one stage of the medicines management cycle are counted once for each stage

Only 14 (4.5%) records described a point‐of‐care electronic medicine administration system, such as barcode administration, with a further eight (2.5%) mentioning electronic medicine administration records. Seven (2.2%) records mentioned keeping electronic adherence data, five of which related to antiretroviral or antituberculosis drugs. Figure 4 shows a breakdown of records by year and the stage of medicines management at which they report system usage or for which digital medicines data are available.

A total of 206 (65.6%) articles describing an EMMS also mentioned the presence of or integration with other electronic health data, such as diagnoses or lab results, and 185 (59.0%) had EHR which were routinely accessible by healthcare providers.

### 3.4 Data sharing, linkage and use of personal ID numbers

Fifty‐nine (18.8%) articles mentioned the use of unique patient identification numbers within an electronic system. These were primarily systems operated at a local level, although there were 13 (4.1%) records (across eight different countries) describing national systems with unique patient identifiers. There were also 83 (2.6%) records which described some form of data sharing across multiple sites, and 32 (10.2%) which described patient‐level linkage (where a patient's record is linked across sites via either their personal details or a unique personal ID).

## 4 DISCUSSION

Our findings demonstrate that the use of EMMS in DCs is far from uncommon, although even among these countries, higher HDI was associated with more reports of EMMS use. We found that systems were often small scale and insular rather than interconnected, as well as being principally implemented in secondary care settings. They were also frequently designed to manage specific workflows associated with certain medical conditions, especially HIV. Despite this, we also found some examples of large‐scale, and, in some cases, nationally operated systems without restriction to patient subpopulations. Contrary to our expectation, most EMMS and medicines usage datasets co‐existed with other electronic health records, such as diagnoses or laboratory results, which has favourable implications for the utility and potential applications of these data. To our knowledge, this is the most comprehensive review of EMMS in DCs to date, which highlights established implementations that may serve as a roadmap for others, as well as settings and geographical locations where investment and development of these systems may be best directed.

There may be significant barriers to the implementation of EMMS in developing countries, especially in rural areas, including substantial upfront capital expenditure, digital literacy and availability, and the existence of a robust technological and workforce infrastructure.^24^ A country's level of ‘development’ can be measured in many ways, with income and economic metrics commonly being used as indicators. In fact, some organizations no longer prefer the term DCs, instead referring to low‐ and middle‐income countries. Devised by the United Nations, HDI is a useful way to measure the health and development of a country and looks at more than just economic prosperity, incorporating measures of life expectancy and education as well as gross national income.^25^ Even if economic circumstances are similar, countries with different levels of educational attainment and life expectancy will have different healthcare challenges and vary in their readiness to adopt digital technologies. By using HDI, our findings may represent a more nuanced picture of where barriers to EMMS implementation, beyond just financial constraints, have been overcome.

Understanding where EMMS are used, and where electronic health datasets exist, is a vital step in identifying potentially untapped routine health data which could be used in research and planning for clinical care. This is particularly important in the context of DCs; often home to large communities underserved by healthcare systems, and who are also frequently underrepresented in health research. We also identified an apparent lack of systems in many countries, which if validated, may have strategic implications at a policy level to inform where funding and deployment of EMMS may be most impactful. Though many barriers to implementation will persist, successful implementations of EMMS and EHR in similar settings is encouraging, including many using affordable and open‐source software. Open‐source platforms provide particularly cost‐effective solutions that are customizable to specific healthcare needs, reducing the financial burden on institutions. Closer collaboration between governments or regional authorities and the providers of such systems could expedite their adoption. Additionally, international cooperation plays a critical role in facilitating EMMS adoption, whether through financial support from organizations such as the World Bank and WHO, or through technical collaborations between countries with similar healthcare challenges. It may also be possible to upscale successful initiatives, such as local or regional EMMS frameworks, though to facilitate this it may be necessary to first deliver or expand programmes aimed at improving digital literacy among healthcare professionals. Using common data standards to ensure interoperability and effective data‐sharing across institutions and regions will be essential in maximizing the benefits of EMMS, both for direct patient care and for large‐scale health data research.

Medicines data is more intuitively structured than many other health and diagnostic datasets. Information about dosage and formulation can easily be systematically quantified, unlike the intricacies of precise diagnoses, treatment regimens and management plans. This is pertinent, because medicines data is often tightly linked to stock management and reimbursement processes. For these reasons, we anticipated that the use of EMMS systems and maintenance of medicine usage data may be more prevalent than comprehensive EHR. Where available, these data might offer a valuable resource for inferring patterns of morbidity in the absence of explicit disease datasets.

Within the included records we noted numerous specific use‐cases of EMMS for improving health outcomes at the point‐of‐care, for example, adherence monitoring for antiretroviral and tuberculosis treatment, and using electronic prescribing to minimize medication errors in high‐risk settings such as neonatal intensive care units (NICUs), intensive therapy units (ITUs), and the delivery of chemotherapy. The latter is one of three key action areas of the WHO Medication Without Harm initiative.^2^ In addition to point‐of‐care applications, we also found various examples of real‐world prescription/dispensing records (in many cases with these data collected via a point‐of‐care EMMS) being used for pharmacoepidemiological study.^26^, ^27^, ^28^, ^29^ Despite this, we found a relative lack of systems that reported the use of a patient ID number, and it was often not possible to establish if systems that are used on a regional or national scale routinely share data or have a common set of patient records across sites. In some cases, this may be due to the articles in question not reporting this information. However, where EMMS are used over multiple sites but do not have shared or linked data, pooling and linkage may often be possible, and we found examples of this within the included studies as well.^30^, ^31^ This could improve not just sample sizes and power for health records research, but also impact direct individual patient care by allowing care providers to see a more complete picture of their patients' health journey; enabling more collaborative care with better continuity between sites and sectors of health systems.

### 4.1 Strengths and limitations

Strengths of this work include the large number of literature databases that were searched, and the systematic approach to identifying and selecting relevant literature. We also considered articles published in any language for inclusion; however, this does not mitigate the fact that searches were only conducted in English, so it is likely that some relevant non‐English articles will not have been identified to start with. This is of particular relevance given that we were investigating the EMMS landscape on an international scale, as pertinent research may be especially likely to be published in a language other than English. The implementation or ongoing use of EMMS is also primarily part of service provision rather than research, so it may not be reported in the academic literature at all. A grey literature search may have helped to address this publication bias but was not performed due in part to feasibility within time constraints. The lack of certainty around the strength of correlation between deployment of an EMMS and scientific publication describing it is an important limitation, meaning this review will underestimate the true extent of deployment. We also present a synthesis of evidence based on number of publications, rather than sites or unique systems. This could result in duplicated reporting of some described EMMS, though these may have undergone updates and changes, or be described differently between articles. It also means that countries, settings and institutions where academic publishing is more commonplace will be overrepresented to some degree. Additionally, we saw that the rate of publication of EMMS‐related articles appears to be increasing, and this would benefit from contextualization and comparison with the rate of publication in countries with high HDI. Unfortunately, because our findings focus exclusively on DCs, this comparison cannot be made.

There were various challenges at the data extraction stage associated with the transformation of diverse and unstructured natural language into a structured data format. This was compounded by widely varying depths and breadths of information about study settings and system characteristics. The specification and description of an EMMS was frequently tangential to the objectives of a given article and, as a result, characterization was often vague or incomplete. We were therefore rarely able to categorically describe a feature as ‘absent’ rather than ‘not reported’. This means that systems are likely underspecified/undercharacterized. This is an unavoidable side‐effect of minimizing the possibility of positive mischaracterization: stating that features or functionality exist where they do not. Future research would be more valuable for researchers and health healthcare managers and leaders if a minimum or standard set of characteristics were used to describe EMMS. We have produced a table with a recommended characteristics for reporting (Table 1), which we hope others may use or adapt in future work.

**TABLE 1:** A recommended minimum set of characteristics for reporting when describing the use of an EMMS.

System name/specification	Setting	Functionality
Software provider Software name Software user (e.g. institution or organization) or data owner Details of implementation (where software is modular or not used ‘as is’ or ‘out of the box’)	Primary or secondary/tertiary care Specific use case (if not used in a general setting) Number of affiliated or related sites in which system is used and/or number of patients served	Management stage (prescribing, dispensing, administration, adherence monitoring, and/or stewardship) Used at point‐of‐care or database/records/data‐input only Presence of routine data sharing across sites Presence of patient linkage and/or presence of unique identifier

## 5 CONCLUSION

EMMS appear to have been adopted to a significant extent in DCs, and there is evidence that in recent years the rate of adoption is accelerating. This may reap health benefits on both an individual patient level as well as more broadly through enhancing research and service planning and provision. Of even greater potential benefit, where EMMS exist, these would seem to commonly be implemented in the context of a broader EHR. This would theoretically enable analysis of patterns of both medicines' usage and disease directly. There are well‐recognized barriers to the digital transformation of healthcare systems in DCs, but we identified many examples where these have been successfully overcome. Still, certain geographical regions and healthcare settings (e.g. northern Africa, primary healthcare and rural settings) were less represented in the sample of systems we identified in the included journal articles. However, learning from success stories with similar contexts may empower others, and guide health and policy leaders in resource‐poor settings when pursuing the implementation of EMMS.

## AUTHOR CONTRIBUTIONS

A.L., R.S., Y.J. and D.W. conceptualized and planned the study. R.S. and Y.J. supervised the work. A.L. and D.W. performed searches and piloted screening and extraction of data. A.L., D.W., T.S., M.B., I.M. and P.A. screened and extracted data. Initial synthesis and data visualization were performed by A.L. A.L. wrote the first draft, and R.S., Y.J., D.W. and R.V.S. provided comments and contributed to the critical revision of the manuscript. All authors read and approved the final draft.

## CONFLICT OF INTEREST STATEMENT

The authors declare no conflicts of interest relating to this work.

## Supporting information


**Table S1** Summary of included articles.
**Table S2** Brief narrative descriptions of included studies.
**Table S3** A list of countries eligible for inclusion.

## Data Availability

The protocol for this review was not prospectively registered. The protocol and data extracted from included studies will be shared by the corresponding author upon reasonable request.

## References

[1] World Health Organization . Global spending on health. World Health Organization; 2022.

[2] World Health Organization . Medication without harm. World Health Organization; 2017.

[3] Albarqouni L , Palagama S , Chai J , et al. Overuse of medications in low‐ and middle‐income countries: a scoping review. Bull World Health Organ. 2023;101(1):36‐61D. doi:10.2471/BLT.22.288293 36593777 PMC9795388

[4] García‐Goñi M Rationalizing pharmaceutical spending 2022 IMF Working Paper 2022 190 doi:10.5089/9798400219849.001

[5] Franklin BD , O'Grady K , Donyai P , Jacklin A , Barber N . The impact of a closed‐loop electronic prescribing and administration system on prescribing errors, administration errors and staff time: a before‐and‐after study. Qual Saf Health Care. 2007;16(4):279‐284. doi:10.1136/qshc.2006.019497 17693676 PMC2464943

[6] Chapuis C , Roustit M , Bal G , et al. Automated drug dispensing system reduces medication errors in an intensive care setting. Crit Care Med. 2010;38(12):2275‐2281. doi:10.1097/CCM.0b013e3181f8569b 20838333

[7] Austin JA , Smith IR , Tariq A . The impact of closed‐loop electronic medication management on time to first dose: a comparative study between paper and digital hospital environments. Int J Pharm Pract. 2018;26(6):526‐533. doi:10.1111/ijpp.12432 29356171

[8] Ahmed Z , Barber N , Jani Y , Garfield S , Franklin BD . Economic impact of electronic prescribing in the hospital setting: a systematic review. Int J Med Inform. 2016;88:1‐7. doi:10.1016/j.ijmedinf.2015.11.008 26878756

[9] Tanne JH . Electronic prescribing could save at least $29bn. BMJ. 2004;328(7449):1155. doi:10.1136/bmj.328.7449.1155 PMC411085 15142905

[10] Samadbeik M , Ahmadi M , Sadoughi F , Garavand A . A comparative review of electronic prescription systems: lessons learned from developed countries. J Res Pharm Pract. 2017;6(1):3‐11. doi:10.4103/2279-042X.200993 28331859 PMC5348854

[11] Zheng WY , Lichtner V , Van Dort BA , Baysari MT . The impact of introducing automated dispensing cabinets, barcode medication administration, and closed‐loop electronic medication management systems on work processes and safety of controlled medications in hospitals: a systematic review. Res Soc Adm Pharm. 2021;17(5):832‐841. doi:10.1016/j.sapharm.2020.08.001 32891535

[12] Herrett E , Gallagher AM , Bhaskaran K , et al. Data resource profile: clinical practice research datalink (CPRD). Int J Epidemiol. 2015;44(3):827‐836. doi:10.1093/ije/dyv098 26050254 PMC4521131

[13] Wakabayashi Y , Eitoku M , Suganuma N . Characterization and selection of Japanese electronic health record databases used as data sources for non‐interventional observational studies. BMC Med Inform Decis Mak. 2021;21(1):167. doi:10.1186/s12911-021-01526-6 34022876 PMC8140583

[14] Platt R , Boudreau D , Haynes K , Gurwitz J , Granger C . NIH Collaboratory Distributed Research Network (DRN). Published online 2019. Accessed June 27, 2025. https://rethinkingclinicaltrials.org/nih-collaboratory-drn/

[15] Gonçalves‐Bradley DC , Maria J , Ricci‐Cabello I , et al. Mobile technologies to support healthcare provider to healthcare provider communication and management of care. Cochrane Database Syst Rev. 2020;2020(8). doi:10.1002/14651858.CD012927.pub2 PMC7437392 32813281

[16] Pega F , Pabayo R , Benny C , Lee EY , Lhachimi SK , Liu SY . Unconditional cash transfers for reducing poverty and vulnerabilities: effect on use of health services and health outcomes in low‐ and middle‐income countries. Cochrane Database Syst Rev. 2017;(3). doi:10.1002/14651858.CD011135.pub3 PMC8962215 35348196

[17] UNDP (United Nations Development Programme) . The next frontier: human development and the Anthropocene. Human Development Report. 2020.

[18] R Core Team . R. Published online 2022. Accessed April 2, 2024. https://www.r-project.org/

[19] Brownrigg R , Minka TP , Deckmyn A . Maps: draw geographical maps. Published online 2021. Accessed April 2, 2024. https://cran.r-project.org/web/packages/maps/index.html

[20] Pebesma, E , Bivand, R , Racine, E , et al. Sf: simple features for R. Published online 2022. Accessed April 2, 2024. h https://cran.r-project.org/web/packages/sf/index.html

[21] Gohel D , Skintzos P , Bostock M . Ggiraph: make “ggplot2” graphics interactive. Published Online 2022. Accessed April 2, 2024. https://cran.r-project.org/web/packages/ggiraph/index.html

[22] Chang W , Cheng J , Allaire J , et al. Shiny: web application framework for R. Published online 2024. Accessed April 2, 2024. https://github.com/rstudio/shiny, https://shiny.posit.co/

[23] Page MJ , McKenzie JE , Bossuyt PM , et al. The PRISMA 2020 statement: an updated guideline for reporting systematic reviews. BMJ. 2021;372:n71. doi:10.1136/bmj.n71 33782057 PMC8005924

[24] Global Health Observatory for eHealth . Global diffusion of ehealth: making universal health coverage achievable: report of the third global survey on ehealth. World Health Organization; 2016.

[25] United Nations Development Programme . Human development index. Human Development reports 2023. Accessed November 30, 2023. https://hdr.undp.org/data-center/human-development-index#/indicies/HDI

[26] Truter I . Drug utilisation study of anti‐obesity products dispensed by pharmacies in South Africa. Value Health. 2017;20(9):A546‐A547. doi:10.1016/j.jval.2017.08.840

[27] Killian JA , Wilder B , Sharma A , Choudhary V , Dilkina B , Tambe M . Learning to prescribe interventions for tuberculosis patients using digital adherence data. In: Proceedings of the 25th ACM SIGKDD international conference on Knowledge Discovery & Data Mining; 2019:2430‐2438.

[28] Kosuma P , Jedsadayanmata A . Prevalence and predictors of statin treatment among patients with chronic heart failure at a tertiary‐care center in Thailand. Clin Med Insights Cardiol. 2019;13:1179546819855656. doi:10.1177/1179546819855656 31217695 PMC6558538

[29] Su L , Li Y , Xu R , et al. Association of ibuprofen prescription with acute kidney injury among hospitalized children in China. JAMA Netw Open. 2021;4(3):e210775. doi:10.1001/jamanetworkopen.2021.0775 33662136 PMC7933997

[30] Lima T d A , Beyrer C , Golub JE , et al. Inequalities in HAART uptake and differential survival according to exposure category in Rio de Janeiro, Brazil. Cad Saude Publica. 2018;34(8):e00009617. doi:10.1590/0102-311X00009617 30133651

[31] Corbell C , Katjitae I , Mengistu A , et al. Records linkage of electronic databases for the assessment of adverse effects of antiretroviral therapy in sub‐Saharan Africa. Pharmacoepidemiol Drug Saf. 2012;21(4):407‐414. doi:10.1002/pds.2252 22009899

