# Supplementary material for: Electronic medicine management systems in developing countries: A landscape review
Source: Br J Clin Pharmacol. 2025 Jul 1;91(9):2507–14. doi: 10.1002/bcp.70156 (PMC12381608; doi:10.1002/bcp.70156)
Supplement: Supplementary file 1 — Table S1 Summary of included articles. Table S2 Brief narrative descriptions of included studies. Table S3 A list of countries eligible for inclusion. [file BCP-91-2507-s001.docx]

Supplementary material

**Section 1: supplementary data tables**

**Supplementary table S1: Summary of included articles**

|  | **Location** | | | **Setting** | | | | | | | **System characteristics** | | | | | | | | | | |
| --- | --- | --- | --- | --- | --- | --- | --- | --- | --- | --- | --- | --- | --- | --- | --- | --- | --- | --- | --- | --- | --- |
|  | Continent | Country | Region/ city | Comm. pharm. | Prim. care | Spec. outpat. | Sec./tert. care | Other | Private | Specific use case | Presc. | Disp. | Admin. | Adh. | EHR | Shared data | Pt. linkage | Database only | PID | Mgmt. level | Total sites |
| Ababneh et al 2020 | Asia | Jordan |  |  |  | • | • |  |  |  | • | • |  |  | • |  |  |  |  | Local | 1 |
| Abbassi et al 2022 | Africa | Tunisia | Tunis |  |  |  | • |  |  | TPN | • |  |  |  |  |  |  |  |  | Local | 1 |
| Abdel-Qader et al 2020 | Asia | Jordan | Amman |  |  |  | • |  |  |  | • |  |  |  | • |  |  |  | • | Local | 1 |
| Abedian et al 2024 | Asia | Iran |  |  | • |  | • |  |  |  | • |  |  |  | • | • |  |  |  | Nat'l |  |
| Abu Asab et al 2019 | Asia | Jordan |  | • | • | • | • |  |  |  | • | • |  |  | • | • | • |  | • | Nat'l |  |
| Abu-Naser et al 2021 | Asia | Jordan | Irbid |  |  |  | • |  |  |  | • |  |  |  | • |  |  |  |  | Local | 1 |
| Afrash et al 2023 | Asia | Iran | Tehran |  |  |  | • |  |  | Cancer | • |  |  |  |  |  |  |  |  | Local | 1 |
| Agh et al 2021 | Europe | North Macedonia |  |  |  |  |  | • |  |  | • |  |  |  |  |  |  |  |  | Nat'l |  |
| Ahmed et al 2014 | Asia | Bangladesh |  |  | • |  |  | • | • |  | • |  |  |  | • |  |  |  | • | Org. | 2 |
| Al Meslamani et al 2021 | Asia | Jordan |  |  |  | • |  |  |  |  | • | • |  |  | • |  |  |  |  | Local | 36 |
| Al Meslamani et al 2022 | Asia | Jordan |  |  | • |  | • |  |  |  | • |  |  |  | • |  |  |  |  | Nat'l |  |
| Alipour et al 2024 | Asia | Iran |  |  |  |  | • |  |  |  | • |  |  |  |  |  |  |  |  | Nat'l |  |
| Allen et al 2007 | Africa | Rwanda |  |  | • | • |  |  |  | HIV | • |  |  |  | • | • |  |  |  | Org. | 6 |
| Amaral et al 2022 | South America | Brazil | Sao Paulo |  |  |  |  | • |  |  | • |  |  |  | • |  |  |  |  | Local |  |
| Amir et al 2022 | Asia | Pakistan | Peshawar |  |  |  | • |  |  |  | • |  |  |  |  |  |  |  |  | Local | 1 |
| Aneja et al 2022 | Asia | India | Bathinda |  |  |  | • |  |  | Psych. telemed. | • |  |  |  |  |  |  |  |  | Local | 1 |
| Arabian et al 2024 | Asia | Iran |  |  | • |  | • |  |  |  | • |  |  |  |  |  |  |  |  | Nat'l |  |
| Asefa et al 2016 | Africa | Namibia |  |  | • |  |  |  |  | HIV |  | • |  |  |  | • |  |  |  | Nat'l |  |
| Aslam et al 2023 | Asia | Pakistan |  |  |  |  |  | • |  | TB | • |  |  |  |  | • | • | • | • | Nat'l |  |
| Atif et al 2022 | Asia | Pakistan |  |  |  |  |  | • |  | TB | • |  |  |  |  | • | • | • | • | Nat'l |  |
| Atthobari et al 2010 | Asia | Indonesia | Yogyakarta |  |  | • |  |  |  |  | • |  |  |  |  |  |  |  |  | Local | 1 |
| Awad et al 2018 | Asia | Lebanon | Beirut |  |  | • | • |  |  |  |  |  |  |  |  |  |  | • | • | Local | 1 |
| Ayala-Estrada et al 2016 | North America | Mexico |  |  |  |  |  | • |  | Growth hormone def. |  |  |  |  |  | • |  |  |  | Org. |  |
| Azim et al 2019 | Asia | Pakistan | Khyber Pakhtunkhwa |  |  |  | • |  | • |  | • | • | • |  | • |  |  |  |  | Local | 1 |
| Aziz et al 2015 | Asia | Pakistan | Lahore |  |  |  | • |  |  | Cancer | • |  |  |  |  |  |  |  |  | Local | 1 |
| Badin et al 2023 | South America | Brazil | Manaus |  |  |  | • |  |  |  | • |  |  |  | • |  |  |  |  | Nat'l |  |
| Baesso et al 2022 | South America | Brazil | Tubarao |  |  |  | • |  |  |  | • |  |  |  | • |  |  |  | • | Local | 1 |
| Bahmani et al 2022 | Asia | Iran |  |  | • |  | • |  |  |  | • |  |  |  |  | • |  |  |  | Nat'l |  |
| Barigela et al 2021 | Asia | India | Nalgonda |  | • |  |  |  |  |  |  |  |  |  | • |  |  |  |  | Local | 2 |
| Bonella et al 2016 | South America | Brazil | Uberlandia |  |  |  | • |  |  |  | • |  |  |  |  |  |  |  |  | Local | 1 |
| Bouh et al 2024 | Asia | Bangladesh |  |  | • |  | • |  |  |  | • |  |  |  | • | • |  |  |  | Nat'l |  |
| Bouraghi et al 2024 | Asia | Iran |  |  | • |  | • |  |  |  | • |  |  |  |  |  |  |  |  | Nat'l |  |
| Buawangpong et al 2020 | Asia | Thailand | Chiang Mai |  | • |  |  |  |  |  | • |  |  |  | • |  |  |  |  | Local | 1 |
| Campos et al 2021 | South America | Brazil | Minas Gerais |  | • |  | • |  |  |  |  | • |  |  |  | • | • |  | • | Reg'l |  |
| Carmona et al 2012 | South America | Brazil | Ribeirao Preto |  |  |  | • |  |  |  | • |  |  |  |  |  |  |  |  | Local | 1 |
| Cassiani et al 2003 | South America | Brazil | Sao Paulo |  |  |  | • |  |  |  | • | • |  |  |  |  |  |  |  | Local | 1 |
| Chai et al 2023 | Asia | China | Zhengzhou |  |  |  | • |  |  |  | • |  |  |  | • |  |  |  |  | Local | 1 |
| Chang et al 2020 | Asia | China | Guizhou |  | • |  |  |  |  |  | • |  |  |  |  | • |  |  |  | Reg'l | 132 |
| Chen et al 2019 | Asia | China | Xiamen |  |  |  | • |  |  |  | • | • |  |  |  |  |  |  |  | Local | 1 |
| Chen et al 2020 | Asia | China | Beijing |  |  |  | • |  |  |  |  |  | • |  | • |  |  |  |  | Local | 1 |
| Chen et al 2022 | Asia | China | Nanjing |  |  |  | • |  |  |  | • |  |  |  | • |  |  |  |  | Local | 1 |
| Chinwong et al 2021 | Asia | Thailand | Chiang Mai |  |  |  | • |  |  |  |  | • |  |  | • |  |  |  |  | Local | 1 |
| Colletti Junior et al 2018 | South America | Brazil |  |  |  |  | • |  |  | Intensive care | • |  |  |  | • |  |  |  |  | Local | 189 |
| Connolly et al 2015 | Asia | China | Shanghai |  |  |  |  | • |  |  |  |  |  |  | • |  |  |  |  | Local |  |
| Corbell et al 2012 | Africa | Namibia |  |  |  | • |  |  |  | HIV |  | • |  |  | • | • | • |  | • | Nat'l | 35 |
| Costa et al 2004 | South America | Brazil | Ribeirao Preto |  |  |  | • |  |  |  | • | • |  |  | • |  |  |  | • | Local | 1 |
| Costa et al 2021 | South America | Brazil | Ribeirao Preto |  |  |  | • |  |  | Intensive care | • |  | • |  | • |  |  |  |  | Local | 1 |
| Cueva et al 2020 | South America | Ecuador | Quito |  |  | • |  |  | • |  | • |  |  |  | • |  |  |  |  | Local | 1 |
| Curtarelli et al 2019 | South America | Brazil | Sao Paulo |  |  |  | • |  |  |  | • |  |  |  | • |  |  |  |  | Local | 1 |
| Cury et al 2019 | South America | Brazil | Campo Grande |  |  | • | • |  | • | Inflamm. bowel disease |  |  |  |  |  |  |  | • |  | Local | 1 |
| Custodio et al 2023 | South America | Brazil |  |  | • |  | • |  |  |  | • |  |  |  | • | • | • |  | • | Nat'l |  |
| da Rocha et al 2021 | South America | Brazil | Curitiba |  |  | • | • |  |  | Cancer | • |  |  |  |  |  |  |  |  | Local | 1 |
| Daher et al 2020 | Asia | Jordan | Amman |  |  |  | • |  |  | NICU | • |  |  |  | • |  |  |  | • | Local | 1 |
| Dao et al 2024 | Asia | Vietnam | Ho Chi Minh City |  |  |  | • |  |  |  |  |  | • |  | • |  |  |  |  | Local | 1 |
| Darawad et al 2019 | Asia | Jordan |  |  |  |  | • |  |  |  |  |  | • |  | • | • | • |  | • | Nat'l | 3 |
| De et al 2021 | Asia | India | Kolkata |  |  | • |  |  |  |  | • |  |  |  | • |  |  |  |  | Local | 1 |
| De Oliveira et al 2020 | South America | Brazil | Ribeirao Preto |  | • |  |  |  |  |  |  | • |  |  |  | • |  | • |  | Reg'l |  |
| de Souza et al 2016 | South America | Brazil | Brasilia |  |  |  | • |  |  | NICU | • |  |  |  | • |  |  |  |  | Local | 1 |
| Dethlefs et al 2019 | North America | Dominican Republic |  |  | • |  |  |  |  | Diabetes and blood pressure |  | • |  |  | • |  |  |  |  | Org. | 2 |
| Diaz et al 2020 | South America | Colombia |  | • | • |  |  |  | • |  | • | • |  |  |  | • |  |  |  | Org. |  |
| Do et al 2023 | Asia | Vietnam | Nam Dinh |  | • |  |  |  |  |  | • |  |  |  |  |  | • |  | • | Local | 48 |
| Donneyong et al 2019 | Africa | Ghana |  |  | • | • | • |  |  |  | • | • |  |  |  | • | • | • | • | Nat'l |  |
| Dorairaj et al 2018 | Asia | India |  |  | • |  |  |  |  |  |  |  |  |  | • | • |  |  |  | Org. | 20 |
| Doubova et al 2014 | North America | Mexico |  |  |  | • |  |  |  |  | • |  |  |  | • |  |  |  |  | Nat'l |  |
| Du et al 2023 | Asia | China | Wuxi |  |  |  | • |  |  | Psychiatry telemedicine | • |  |  |  | • |  |  |  |  | Local | 1 |
| Duangsong et al 2022 | Asia | Thailand |  |  |  | • | • |  |  |  | • |  |  |  | • |  |  |  |  | Local | 2 |
| Elhawary et al 2022 | Africa | Egypt | Cairo |  |  |  | • |  |  |  | • |  |  |  |  |  |  |  |  | Local | 1 |
| Elias et al 2017 | South America | Brazil | Sao Paulo |  |  | • | • |  |  |  | • |  |  |  | • |  |  |  |  | Local | 1 |
| Fan et al 2019 | Asia | China | Beijing |  |  |  | • |  |  |  | • |  |  |  | • |  |  |  |  | Local | 1 |
| Fatima et al 2024 | Asia | Pakistan | Karachi |  |  |  | • |  |  |  | • |  |  |  |  |  |  |  |  | Local | 1 |
| Ferracini et al 2016 | South America | Brazil | Sao Paulo |  |  |  | • |  |  |  | • |  |  |  |  |  |  |  |  | Local | 1 |
| Fraser et al 2013 | South America | Peru |  |  | • | • |  |  |  | MDR-TB | • | • |  |  | • | • |  |  |  | Nat'l |  |
| Fu et al 2019 | Asia | China | Dongcheng district |  | • |  |  |  |  |  | • |  |  |  | • |  |  |  |  | Reg'l | 66 |
| Fumis et al 2012 | South America | Brazil | Sao Paulo |  |  |  | • |  |  |  | • |  | • |  |  |  |  |  |  | Local | 1 |
| Ghadieh et al 2015 | Asia | Lebanon | Beirut |  | • |  |  |  |  |  |  |  |  |  | • |  |  |  |  | Local | 1 |
| Gimenes et al 2019 | South America | Brazil |  |  |  |  | • |  |  |  | • |  |  |  |  |  |  |  |  | Local | 3 |
| Gimenes et al 2006 | South America | Brazil | Sao Paulo |  |  |  | • |  |  |  | • |  |  |  |  |  |  |  |  | Local | 1 |
| Gonzalez-Perez et al 2019 | North America | Mexico |  |  | • |  |  |  |  |  |  |  |  |  | • |  |  |  |  | Local | 1 |
| Goodman-Meza et al 2021 | North America | Mexico |  | • |  | • | • |  |  |  | • | • |  |  |  | • |  | • |  | Nat'l |  |
| Green et al 2015 | Africa | Kenya |  |  | • | • |  |  |  |  | • |  |  | • | • | • |  |  |  | Nat'l |  |
| Gu et al 2023 | Asia | China | Zunyi |  |  |  | • |  |  |  | • | • |  |  | • |  |  |  | • | Local | 1 |
| Guan et al 2021 | Asia | China | Beijing |  |  |  | • |  |  |  | • |  |  |  | • |  |  |  |  | Local | 1 |
| Guidoni et al 2016 | South America | Brazil | Ribeirao Preto |  |  |  | • |  |  |  | • |  |  |  |  |  |  |  |  | Local | 1 |
| Guidoni et al 2014 | South America | Brazil | Ribeirao Preto |  |  |  | • |  |  |  | • |  |  |  |  |  |  |  | • | Local | 1 |
| Guo et al 2021 | Asia | China | Beijing |  | • |  | • |  |  |  | • |  |  |  | • | • | • |  |  | Reg'l |  |
| Guo et al 2021 | Asia | China | Beijing |  |  |  | • |  |  |  | • |  |  |  | • |  |  |  |  | Local | 1 |
| Hampapur et al 2017 | Asia | India | Mumbai |  |  |  | • |  |  |  | • | • |  |  | • |  |  |  | • | Local | 1 |
| Haskew et al 2015 | Africa | Kenya |  |  |  | • | • |  |  | Child and maternal health |  |  |  |  | • | • |  |  |  | Org. | 5 |
| He et al 2023 | Asia | China | Shaoguan |  | • |  | • |  |  |  | • |  |  |  | • | • | • |  | • | Reg'l | 37 |
| Hernandez-Avila et al 2015 | North America | Mexico | Colima |  |  | • |  |  |  |  | • |  | • |  | • | • |  |  |  | Reg'l | 117 |
| Hinojosa-Amaya et al 2016 | North America | Mexico | Nuevo Leon |  |  |  | • |  |  |  | • | • | • |  | • |  |  |  |  | Local | 1 |
| Hitti et al 2017 | Asia | Lebanon | Beirut |  |  |  | • |  | • | A+E/ER | • |  |  |  | • |  |  |  | • | Local | 1 |
| Hoagland et al 2020 | South America | Brazil | Rio de Janeiro |  |  | • | • |  |  | HIV | • |  |  |  |  |  |  |  |  | Local | 1 |
| Hossain et al 2019 | Asia | Bangladesh | Bheramara |  | • |  |  | • |  |  | • |  |  |  | • | • | • |  | • | Org. | 32 |
| Hua 2016 | Asia | China | Wuhan |  |  |  | • |  |  |  | • |  |  |  | • |  |  |  | • | Local | 1 |
| Hua et al 2020 | Asia | China | Wuhan |  |  |  | • |  |  |  | • | • |  |  | • |  |  |  | • | Local | 1 |
| Huang et al 2020 | Asia | China | Hunan Province |  |  |  | • |  |  |  | • |  |  |  | • |  |  |  |  | Local | 3 |
| Idrees et al 2018 | North America | Honduras | San Jose |  | • |  |  |  |  |  |  | • |  |  |  |  |  |  |  | Local | 1 |
| Jazayeri et al 2003 | North America | Haiti |  |  |  | • |  |  |  | HIV | • |  |  |  | • | • |  |  |  | Org. | 6 |
| Jebraeily et al 2024 | Asia | Iran |  |  |  |  | • |  |  |  | • |  |  |  |  |  |  |  |  | Nat'l |  |
| Jeddi et al 2024 | Asia | Iran |  |  | • | • |  |  |  |  | • |  |  |  |  |  |  |  |  | Nat'l |  |
| Jenghua et al 2020 | Asia | Thailand |  |  |  | • | • |  |  |  | • |  |  |  | • |  |  |  |  | Local | 2 |
| Jenghua et al 2022 | Asia | Thailand |  |  |  | • | • |  |  |  | • |  |  |  | • |  |  |  |  | Local | 2 |
| Jiang et al 2024 | Asia | China | Nanjing |  |  |  | • |  |  |  | • |  |  |  | • |  |  |  |  | Local | 1 |
| Jiao et al 2020 | Asia | China | Sichuan Province |  |  | • | • |  |  |  | • |  |  |  | • |  |  |  |  | Local |  |
| Jokar et al 2023 | Asia | Iran | Shiraz |  |  |  | • |  |  | Anaesthesia | • |  |  |  | • |  |  |  |  | Local | 1 |
| Joseph et al 2020 | Asia | India | Kerala |  |  | • | • |  | • |  |  |  |  |  | • |  |  |  |  | Local | 1 |
| Joshi et al 2024 | Asia | India | Mira Bhayandar |  |  |  | • |  |  |  | • |  |  |  | • |  |  |  |  | Local | 1 |
| Kadayaprath et al 2024 | Asia | India | New Delhi |  |  |  | • |  |  |  | • |  |  |  | • |  |  |  |  | Local | 1 |
| Kandasamy et al 2021 | Asia | India | Coimbatore |  |  |  | • |  | • |  | • |  |  |  |  |  |  |  |  | Local | 1 |
| Karajizadeh et al 2021 | Asia | Iran | Shiraz |  |  |  | • |  |  |  | • |  |  |  | • |  |  |  |  | Local | 1 |
| Karajizadeh et al 2023 | Asia | Iran | Shiraz |  |  |  | • |  |  |  | • |  |  |  | • |  |  |  |  | Local | 1 |
| Kassem et al 2021 | Africa | Egypt |  | • |  |  |  |  |  |  | • |  |  |  |  |  |  |  |  | Local |  |
| Kazemi et al 2011 | Asia | Iran | Hamadan Province |  |  |  | • |  |  |  | • |  | • |  | • |  |  |  |  | Reg'l | 15 |
| Kazemi et al 2010 | Asia | Iran | Hamadan province |  |  |  | • |  |  |  | • |  |  |  | • |  |  |  |  | Reg'l | 15 |
| Kenwy et al 2019 | Africa | Egypt | Cairo |  |  | • | • |  |  |  | • |  |  |  |  |  |  |  | • | Local | 1 |
| Khammarnia et al 2018 | Asia | Iran | Shiraz |  |  |  | • |  |  |  | • |  |  |  |  |  |  |  |  | Local | 1 |
| Khammarnia et al 2017 | Asia | Iran | Shiraz |  |  |  | • |  |  | Intensive care | • |  |  |  |  |  |  |  |  | Local | 1 |
| Khan et al 2020 | Asia | India |  |  | • | • |  |  | • |  | • |  |  |  | • | • |  |  |  | Org. |  |
| Khan et al 2018 | Asia | Pakistan | Karachi |  |  |  | • |  |  |  | • |  |  |  |  |  |  |  |  | Local | 1 |
| Khan et al 2021 | Asia | India |  |  |  |  | • |  |  |  | • |  |  |  | • | • |  |  |  | Org. | 150 |
| Khowaja et al 2008 | Asia | Pakistan | Karachi |  |  |  | • |  |  |  | • | • | • |  |  |  |  |  |  | Local | 1 |
| Killian et al 2019 | Asia | India | Mumbai |  |  | • |  |  |  | TB |  |  |  | • |  | • |  |  |  | Reg'l | 252 |
| Kimaiyo et al 2005 | Africa | Kenya |  |  |  | • | • |  |  | HIV |  | • |  | • | • | • | • |  | • | Org. | 7 |
| Koopmans et al 2018 | Africa | South Africa | Cape Town |  |  |  | • |  |  |  |  | • |  |  |  |  |  |  |  | Local | 1 |
| Kosuma et al 2019 | Asia | Thailand | Phitsanulok |  |  | • |  |  |  |  | • |  |  |  | • |  |  |  |  | Local | 1 |
| Kruger et al 2021 | Africa | South Africa |  |  |  |  | • |  |  | AMS | • |  |  |  |  |  |  | • |  | Local |  |
| Kruse et al 2022 | Africa | South Africa | Durban |  |  |  | • |  |  |  | • |  |  |  | • |  |  |  |  | Local | 1 |
| Kruse et al 2024 | Africa | South Africa | Durban |  |  |  | • |  |  |  | • |  |  |  | • |  |  |  |  | Local | 1 |
| Kwiatkowska et al 2020 | Asia | China | Anhui Province |  | • |  |  |  |  |  | • |  |  |  | • |  |  |  | • | Reg'l | 1367 |
| Lakkis et al 2015 | Asia | Lebanon | Beirut |  | • |  |  |  |  |  |  |  |  |  | • |  |  |  |  | Local | 1 |
| Lan et al 2021 | Asia | China | Lishui |  |  |  | • |  |  |  | • |  |  |  | • |  |  |  |  | Local | 1 |
| Larasati et al 2024 | Asia | Indonesia | Yogyakarta |  |  |  | • |  |  |  | • |  |  |  | • |  |  |  |  | Local | 1 |
| Layton et al 2005 | Asia | Thailand | Khon Kaen |  |  | • |  |  |  |  | • |  |  |  |  |  |  | • |  | Local | 1 |
| Le et al 2024 | Asia | China | Beijing |  |  |  | • |  |  |  | • |  |  |  | • |  |  |  |  | Local | 1 |
| Leal et al 2020 | South America | Brazil | Rio Grande do Sul |  |  |  | • |  | • |  | • |  |  |  | • |  |  |  |  | Local | 1 |
| Lestari et al 2024 | Asia | Indonesia |  |  |  |  |  | • |  | TB | • |  |  |  |  | • |  |  |  | Nat'l |  |
| Li et al 2014 | Asia | China | Chengdu |  |  |  | • |  |  |  | • |  |  |  | • |  |  |  |  | Local | 1 |
| Li, Foy et al 2018 | North America | Honduras | San Jose |  | • |  |  |  |  |  |  | • |  |  |  |  |  |  |  | Local | 1 |
| Li, Sheyu et al 2018 | Asia | China | Chengdu |  |  |  | • |  |  |  | • |  |  |  | • |  |  |  | • | Local | 1 |
| Li, Zhang et al 2013 | Asia | China | Xi An |  |  |  | • |  |  |  | • |  | • |  | • |  |  |  |  | Local | 1 |
| Li, Zhou et al 2013 | Asia | China | Hangzhou |  |  |  | • |  |  |  | • |  |  |  | • |  |  |  |  | Local | 1 |
| Liang et al 2018 | Africa | Uganda |  |  |  |  | • |  |  |  | • |  |  |  | • |  |  |  | • | Org. | 2 |
| Liang et al 2021 | Asia | China |  |  |  |  | • |  |  |  |  |  |  |  | • |  |  |  |  | Nat'l | 16000 |
| Lim et al 2021 | Asia | Thailand | Ubon Ratchathani |  |  |  | • |  |  |  | • |  |  |  | • |  |  |  |  | Local | 1 |
| Lima et al 2014 | South America | Brazil | Rio de Janeiro |  |  |  | • |  |  |  | • |  |  |  | • |  |  |  |  | Local | 1 |
| Lima et al 2018 | South America | Brazil |  |  |  | • |  | • |  | HIV | • | • |  |  | • | • | • |  | • | Nat'l |  |
| Limsomwong et al 2023 | Asia | Thailand | Hat Yai |  |  |  | • |  |  |  | • |  |  |  | • |  |  |  |  | Local | 1 |
| Lin et al 2018 | Asia | China | Yinzhou |  | • | • | • |  |  |  | • | • |  |  | • | • | • |  | • | Reg'l | 294 |
| Liu et al 2015 | Asia | China |  |  |  | • |  |  |  | TB |  |  |  |  |  | • |  |  |  | Org. | 18 |
| Liu et al 2016 | Asia | China | Qian Jiang City |  | • |  |  |  |  |  | • |  |  |  | • |  |  |  |  | Reg'l | 20 |
| Liu et al 2013 | Africa | Kenya |  |  |  | • |  |  |  | HIV | • | • |  |  | • | • | • |  | • | Nat'l | 23 |
| Liu et al 2022 | Asia | China | Guangzhou |  |  |  | • |  |  |  | • |  |  |  | • |  |  |  |  | Local | 1 |
| Lobo et al 2017 | South America | Brazil | Porto Alegre |  |  |  | • |  |  |  | • |  |  |  |  |  |  |  |  | Local | 1 |
| Lyu et al 2022 | Asia | China | Guangzhou |  |  |  | • |  |  |  | • |  |  |  | • |  |  |  |  | Local | 1 |
| Maarsingh et al 2022 | Africa | Uganda | Mukono District |  |  |  |  | • |  | Short-term medical missions | • | • |  |  | • | • | • |  | • | Org. |  |
| Mabirizi et al 2018 | Africa | Namibia |  | • | • | • | • |  |  | HIV |  | • |  | • |  | • |  |  |  | Nat'l | 87 |
| Madruga et al 2018 | South America | Brazil |  |  |  | • |  |  |  | HIV | • | • |  |  | • | • | • |  |  | Nat'l |  |
| Maisso et al 2009 | South America | Brazil |  |  |  |  | • |  |  |  | • |  |  |  |  |  |  |  |  | Local | 1 |
| Maksymovych et al 2020 | Europe | Ukraine |  | • | • |  |  |  |  | 23 WHO essential drugs | • |  |  |  |  | • |  |  |  | Nat'l | 7840 |
| Mashoka et al 2019 | Africa | Tanzania | Dar es Salaam |  |  |  | • |  |  | A+E/ER | • |  |  |  | • |  |  |  |  | Local | 1 |
| Messou et al 2011 | Africa | Cote D'Ivoire | Abidjan |  |  | • |  |  |  | HIV | • |  |  |  |  |  |  | • |  | Local | 3 |
| Miao et al 2020 | Asia | China | Sichuan Province |  |  |  | • |  |  |  | • |  |  |  | • |  |  |  |  | Local | 5 |
| Migowa et al 2018 | Africa | Kenya | Nairobi |  |  | • | • |  | • |  | • |  |  |  |  |  |  |  |  | Local | 1 |
| Miranda-Chavez et al 2024 | South America | Peru | Tacna |  |  |  | • |  |  |  | • |  | • |  | • |  |  |  |  | Local | 1 |
| Mohammad et al 2020 | Africa | South Africa | Durban |  |  |  | • |  |  |  | • |  |  |  | • |  |  |  |  | Local | 1 |
| Moura et al 2011 | South America | Brazil | Vitoria da Conquista |  |  |  | • |  |  |  |  |  |  |  |  | • |  | • |  | Nat'l |  |
| Mwita et al 2022 | Africa | Tanzania | Mwanza |  |  |  | • |  |  |  | • |  |  |  | • |  |  |  |  | Local | 1 |
| Nabovati et al 2016 | Asia | Iran | Khorasan Razavi |  |  |  |  | • |  |  | • | • |  |  |  | • |  | • |  | Reg'l |  |
| Namnabati et al 2017 | Asia | Iran | Isfahan |  |  |  | • |  |  | NICU | • |  | • |  |  |  |  |  |  | Local | 1 |
| Naz et al 2021 | Asia | Pakistan |  |  |  |  |  | • |  | TB | • |  |  |  |  | • |  | • |  | Nat'l |  |
| Nguyen et al 2023 | Asia | Vietnam | Nam Dinh province |  | • |  |  |  |  |  | • |  |  |  | • |  | • |  | • | Local | 112 |
| Niazkhani et al 2020 | Asia | Iran | Urmia |  |  | • | • |  |  | Renal Tx | • |  |  |  |  |  |  |  |  | Local | 1 |
| Novriani et al 2015 | Asia | Indonesia | Jakarta |  |  | • | • |  |  | TB |  |  |  |  | • |  |  |  |  | Local | 1 |
| Nucita et al 2009 | Africa | Mozambique |  |  |  |  |  | • |  | HIV | • |  |  |  | • | • |  |  |  | Org. | 14 |
| Nucita et al 2009 | Africa | Malawi |  |  |  |  |  | • |  | HIV | • |  |  |  | • | • |  |  |  | Org. | 9 |
| Nucita et al 2009 | Africa | Tanzania |  |  |  |  |  | • |  | HIV | • |  |  |  | • | • |  |  |  | Org. | 2 |
| Nucita et al 2009 | Africa | Kenya |  |  |  |  |  | • |  | HIV | • |  |  |  | • | • |  |  |  | Org. | 3 |
| Nucita et al 2009 | Africa | Guinea |  |  |  |  |  | • |  | HIV | • |  |  |  | • | • |  |  |  | Org. | 1 |
| Nucita et al 2009 | Africa | Guinea Bissau |  |  |  |  |  | • |  | HIV | • |  |  |  | • | • |  |  |  | Org. | 1 |
| Nucita et al 2009 | Africa | Cameroon |  |  |  |  |  | • |  | HIV | • |  |  |  | • | • |  |  |  | Org. | 1 |
| Nucita et al 2009 | Africa | Congo |  |  |  |  |  | • |  | HIV | • |  |  |  | • | • |  |  |  | Org. | 1 |
| Nucita et al 2009 | Africa | Angola |  |  |  |  |  | • |  | HIV | • |  |  |  | • | • |  |  |  | Org. | 1 |
| Nucita et al 2009 | Africa | Nigeria |  |  |  |  |  | • |  | HIV | • |  |  |  | • | • |  |  |  | Org. | 2 |
| Nunez-Sanchez et al 2016 | North America | Mexico |  |  |  |  | • |  |  |  | • |  |  |  |  |  |  |  |  | Local | 2 |
| Obreli-Neto et al 2011 | South America | Brazil | Salto Grande |  |  |  | • |  |  |  | • | • |  |  | • |  |  |  |  | Local | 1 |
| Okumura et al 2016 | South America | Brazil | Porto Alegre |  |  |  | • |  |  |  | • | • |  |  |  |  |  |  |  | Local | 1 |
| Oluoch et al 2016 | Africa | Kenya | Siaya County |  |  | • | • |  |  | HIV |  |  |  |  | • |  |  |  |  | Org. | 13 |
| Oreagba et al 2016 | Africa | Nigeria |  |  |  |  | • |  |  |  | • |  |  |  |  |  |  |  |  | Local | 1 |
| Orrell et al 2012 | Africa | South Africa | Masiphumelele | • |  |  |  |  |  |  |  | • |  |  | • |  |  |  |  | Local | 1 |
| Otieno et al 2021 | Africa | Ghana |  |  | • | • |  | • |  | Blood pressure | • |  |  |  | • |  |  |  |  | Org. | 5 |
| Otieno et al 2021 | Africa | Kenya |  |  | • | • |  | • |  | Blood pressure | • |  |  |  | • |  |  |  |  | Org. | 4 |
| Owusu et al 2018 | Africa | Ghana |  |  | • |  |  |  |  |  | • |  |  |  |  |  |  |  |  | Local |  |
| Oza et al 2017 | Africa | Sierra Leone | Kerry Town |  |  |  | • |  |  |  | • |  | • |  | • |  |  |  | • | Local | 1 |
| Pannoi et al 2024 | Asia | Thailand | Hat Yai |  | • |  | • |  |  |  | • |  |  |  | • |  |  |  |  | Local | 1 |
| Peerawaranun et al 2024 | Asia | Thailand |  |  | • |  | • |  |  |  | • |  |  |  | • | • | • |  | • | Nat'l |  |
| Periasamy et al 2023 | Asia | India | Tamil Nadu |  |  | • |  |  | • | Sexual health | • |  |  |  |  | • |  |  |  | Org. |  |
| Petrushevska et al 2015 | Europe | North Macedonia |  | • | • |  |  |  |  |  | • | • |  |  |  | • |  |  |  | Nat'l |  |
| Pina-Hincapie et al 2020 | South America | Colombia | Bogota |  |  |  | • |  |  |  | • |  |  |  | • |  |  |  |  | Local | 1 |
| Pinto Pizzo et al 2015 | South America | Brazil | Sao Paulo |  |  |  | • |  |  |  | • |  |  |  |  |  |  |  |  | Local | 1 |
| Pongwecharak et al 2007 | Asia | Thailand | Songkhla Province |  |  |  | • |  |  |  | • |  |  |  |  |  |  | • |  | Local | 1 |
| Prasert et al 2019 | Asia | Thailand |  |  |  | • | • |  |  |  | • |  |  |  | • | • |  |  |  | Nat'l |  |
| Prasert et al 2018 | Asia | Thailand |  |  |  | • | • |  |  |  | • | • |  |  |  | • |  | • |  | Nat'l |  |
| Prattanaprateep et al 2017 | Asia | Thailand | Bangkok |  |  | • | • |  |  |  | • |  |  |  |  |  |  | • | • | Local | 1 |
| Priya et al 2017 | Asia | India | Kochi |  |  |  | • |  |  |  | • |  |  |  |  |  |  |  |  | Local | 1 |
| Purssell et al 2017 | Asia | Pakistan | Lahore |  |  |  | • |  |  |  | • |  |  |  |  |  |  |  |  | Local | 1 |
| Puspitasari et al 2022 | Asia | Indonesia | Bandung City |  | • |  |  |  |  |  | • |  |  |  | • |  |  |  |  | Local | 1 |
| Puttkammer et al 2020 | North America | Haiti |  |  | • | • | • |  |  |  |  | • |  |  | • | • |  |  |  | Nat'l | 120 |
| Puttkammer et al 2016 | North America | Haiti |  |  | • |  |  |  |  |  | • | • |  |  | • | • |  |  |  | Nat'l | 124 |
| Puttkammer et al 2018 | North America | Haiti |  |  |  |  |  | • |  |  |  | • |  |  | • | • |  |  |  | Nat'l |  |
| Puttkammer et al 2014 | North America | Haiti |  |  | • | • | • |  |  |  | • | • |  | • | • | • |  |  |  | Nat'l | 100 |
| Qin et al 2017 | Asia | China |  |  |  |  | • |  |  |  | • |  |  |  | • |  |  |  | • | Local | 1 |
| Qiu et al 2018 | Asia | China |  |  |  | • | • |  |  |  | • | • |  |  | • |  |  |  | • | Local | 2 |
| Qureshi et al 2021 | Asia | Pakistan | Karachi |  |  |  | • |  | • |  | • |  |  |  |  |  |  |  |  | Local | 1 |
| Raeesi et al 2021 | Asia | Iran |  |  |  | • | • |  |  |  | • | • |  |  | • |  |  |  |  | Nat'l | 349 |
| Rafael et al 2019 | South America | Brazil | Sao Paulo |  |  |  | • | • | • | PN | • |  |  |  |  |  |  |  |  | Org. |  |
| Raiman et al 2015 | Africa | South Africa | Cape Town |  |  |  | • |  |  |  | • | • |  |  |  |  |  |  | • | Local | 1 |
| Ratchakit-Nedsuwan et al 2020 | Asia | Thailand | Chiang Rai |  |  | • |  |  |  | TB |  |  |  |  |  |  |  |  |  | Local | 1 |
| Rattanachotphanit et al 2020 | Asia | Thailand |  |  |  | • | • |  |  |  | • |  |  |  | • | • |  |  |  | Nat'l | 865 |
| Rattanaumpawan et al 2018 | Asia | Thailand | Bangkok |  |  |  | • |  |  |  | • | • |  |  |  |  |  |  |  | Local | 1 |
| Riaz et al 2014 | Asia | Pakistan | Lahore |  |  |  | • |  |  |  | • |  |  |  |  |  |  |  |  | Local | 1 |
| Rosa et al 2019 | South America | Brazil | Belo Horizonte |  |  |  | • |  |  |  | • | • |  |  |  |  |  |  |  | Local | 2 |
| Roy et al 2020 | Africa | Zambia |  |  | • | • |  |  |  |  |  |  |  |  | • | • |  |  |  | Nat'l |  |
| Roy et al 2023 | Asia | India |  |  |  |  |  | • |  | Ophth. | • |  |  |  | • | • | • |  |  | Org. | 195 |
| Ruiz-Talero et al 2020 | South America | Colombia | Bogota |  |  |  | • |  |  |  | • |  |  |  | • |  |  |  |  | Local | 1 |
| Sae-Ang et al 2023 | Asia | Thailand | Hat Yai |  | • |  | • |  |  |  | • |  |  |  | • |  |  |  |  | Local | 1 |
| Sahu et al 2023 | Asia | India |  |  | • |  |  |  |  |  | • |  |  |  |  |  |  |  |  | Local |  |
| Saleem et al 2019 | Asia | Pakistan | Punjab |  |  |  | • |  |  | AMS | • |  |  |  |  |  |  |  |  | Local | 34 |
| Sanchez et al 2023 | South America | Ecuador | Quito |  | • |  |  |  |  |  | • |  |  |  | • |  |  |  |  | Reg'l | 21 |
| Santos et al 2015 | South America | Brazil | Brasilia |  |  |  | • |  |  |  | • |  |  |  | • |  |  |  |  | Local | 1 |
| Santos Garcia et al 2020 | South America | Brazil | Porto Alegre |  | • |  |  |  |  |  |  |  |  |  | • |  |  |  |  | Local | 1 |
| Santoyo-Fexas et al 2020 | North America | Mexico | Monterrey |  |  | • | • |  |  | Rheum. | • |  |  |  |  |  |  |  |  | Local | 1 |
| Serafim et al 2010 | South America | Brazil | Ribeirao Preto |  |  |  | • |  |  |  | • | • | • |  |  |  |  |  | • | Local | 1 |
| Seshadri et al 2018 | Asia | India |  |  |  | • |  |  | • | Diabetes | • |  |  |  | • | • |  |  |  | Org. | 35 |
| Shang et al 2024 | Asia | China | Nanjing |  |  |  | • |  |  |  | • |  |  |  | • |  |  |  |  | Local | 1 |
| Shawahna et al 2011 | Asia | Pakistan | Lahore |  |  |  | • |  |  |  | • |  |  |  | • |  |  |  |  | Local | 1 |
| Shen et al 2020 | Asia | China |  |  |  | • | • |  |  | Cancer |  |  |  |  | • | • |  |  |  | Org. | 12 |
| Siddiqui et al 2007 | Asia | Pakistan | Karachi |  |  |  | • |  |  |  | • |  |  |  |  |  |  |  |  | Local | 1 |
| Silva et al 2008 | South America | Brazil |  |  |  |  | • |  |  |  | • | • |  |  |  |  |  |  |  | Local | 1 |
| Singh et al 2023 | Asia | India | Chandigarh |  |  | • |  |  |  |  | • |  |  |  |  |  |  |  |  | Local | 1 |
| Skosana et al 2021 | Africa | South Africa |  |  |  |  | • |  |  | AMS | • |  |  |  |  |  |  | • |  | Local |  |
| Souza e Silva et al 2015 | South America | Brazil | Tubarao |  |  |  | • |  |  |  | • |  |  |  | • |  |  |  |  | Local | 1 |
| Su et al 2021 | Asia | China |  |  |  |  | • |  |  |  | • |  |  |  | • |  |  |  |  | Local | 25 |
| Surapat et al 2021 | Asia | Thailand | Bangkok |  |  |  | • |  |  |  | • |  |  |  | • |  |  |  |  | Local | 1 |
| Syarkevych et al 2016 | Europe | Ukraine |  |  |  |  |  | • |  | Type 1 diabetes | • |  |  |  |  | • |  | • |  | Nat'l |  |
| Syowai et al 2020 | Africa | Kenya |  | • | • | • |  |  |  | HIV | • | • |  |  | • | • |  |  | • | Nat'l | 918 |
| Tan et al 2024 | Asia | China | Xiamen |  | • |  | • |  |  |  | • |  |  |  | • | • | • |  | • | Reg'l | 326 |
| Tan et al 2022 | Asia | China | Guangzhou |  | • |  |  |  |  |  | • |  |  |  |  | • |  |  |  | Reg'l | 13 |
| Tanaka et al 2015 | South America | Brazil | Sao Paulo |  |  |  | • |  |  | Paeds nutrition | • | • |  |  |  |  |  |  |  | Local | 1 |
| Tang et al 2019 | Asia | China | Shanghai |  |  |  | • |  |  |  | • |  | • |  | • |  |  |  | • | Local | 1 |
| Tansitpong et al 2020 | Asia | Thailand |  |  |  | • | • |  |  |  | • |  |  |  |  | • | • | • | • | Nat'l |  |
| Thompson et al 2010 | Africa | Nigeria | Kaduna |  | • | • |  |  |  | Child and maternal health |  |  |  |  | • |  |  |  |  | Local | 1 |
| Tian et al 2022 | Asia | China | Chengdu |  |  | • |  |  |  |  | • |  |  |  | • | • |  |  |  | Reg'l |  |
| Tierney et al 2010 | Africa | Kenya |  |  | • | • |  |  |  | HIV |  |  |  | • | • |  |  |  | • | Org. | 23 |
| Tierney et al 2010 | Africa | Tanzania |  |  | • | • |  |  |  | HIV |  |  |  | • | • |  |  |  | • | Org. | 3 |
| Tierney et al 2010 | Africa | Uganda |  |  | • | • |  |  |  | HIV |  |  |  | • | • |  |  |  | • | Org. | 3 |
| Tilahun et al 2015 | Africa | Ethiopia |  |  |  |  | • |  |  |  | • |  |  |  | • |  |  |  |  | Nat'l | 5 |
| Tong et al 2023 | Asia | China | Chengdu |  |  |  | • |  |  |  | • |  |  |  | • |  |  |  |  | Local | 1 |
| Tragulpiankit et al 2014 | Asia | Thailand | Suphan Buri |  |  |  | • |  |  |  |  |  |  |  | • |  |  |  |  | Local | 1 |
| Truter 2016 | Africa | South Africa |  | • |  |  |  |  | • |  | • | • |  |  |  | • | • |  | • | Org. | 327 |
| Truter 2001 | Africa | South Africa |  | • |  |  |  |  | • |  | • | • |  |  |  | • | • | • | • | Org. |  |
| Truter 2017 | Africa | South Africa |  | • |  |  |  |  | • |  | • | • |  |  |  | • |  |  |  | Org. |  |
| Truter 2018 | Africa | South Africa |  | • | • |  | • |  |  |  |  | • |  |  |  | • |  | • |  | Org. |  |
| Truter et al 2016 | Africa | South Africa |  | • |  |  |  |  | • |  | • | • |  |  |  | • | • |  | • | Org. | 327 |
| Truter et al 2004 | Africa | South Africa |  | • |  |  |  |  | • |  | • | • |  |  |  | • |  |  |  | Org. |  |
| Vaidotas et al 2019 | South America | Brazil | Sao Paulo |  |  |  | • |  |  |  | • |  | • |  | • | • |  |  |  | Org. | 4 |
| Valencia et al 2018 | South America | Peru | Surquillo |  |  | • |  |  |  | Cancer | • |  | • |  | • |  |  |  | • | Local | 1 |
| Van Der Hoven et al 2022 | Africa | South Africa | The Western Cape |  | • |  |  |  |  |  |  | • |  |  |  | • | • |  | • | Reg'l |  |
| Varavithya et al 2022 | Asia | Thailand | Khlong Nueng |  |  | • | • |  |  |  | • |  |  |  | • |  |  |  |  | Local | 1 |
| Vatcharavongvan et al 2017 | Asia | Thailand | Pathum-Thani |  | • |  |  |  |  |  | • |  |  |  | • |  |  |  |  | Local | 1 |
| Vatcharavongvan et al 2019 | Asia | Thailand | Pathum-Thani |  | • |  |  |  |  |  | • |  |  |  | • |  |  |  | • | Local | 1 |
| Vatcharavongvan et al 2021 | Asia | Thailand |  |  | • |  |  |  |  |  | • |  |  |  | • |  |  |  |  | Local | 8 |
| Volpe et al 2016 | South America | Brazil | Brasilia |  |  |  | • |  |  |  | • |  | • |  | • |  |  |  | • | Local | 1 |
| Vonasek et al 2021 | Africa | Botswana |  |  |  | • |  |  |  | HIV |  |  |  |  | • | • |  |  |  | Org. | 1 |
| Vonasek et al 2021 | Africa | Eswatini |  |  |  | • |  |  |  | HIV |  |  |  |  | • | • |  |  |  | Org. | 1 |
| Vonasek et al 2021 | Africa | Lesotho |  |  |  | • |  |  |  | HIV |  |  |  |  | • | • |  |  |  | Org. | 1 |
| Vonasek et al 2021 | Africa | Malawi |  |  |  | • |  |  |  | HIV |  |  |  |  | • | • |  |  |  | Org. | 1 |
| Vonasek et al 2021 | Africa | Tanzania |  |  |  | • |  |  |  | HIV |  |  |  |  | • | • |  |  |  | Org. | 2 |
| Vonasek et al 2021 | Africa | Uganda |  |  |  | • |  |  |  | HIV |  |  |  |  | • | • |  |  |  | Org. | 1 |
| Wang et al 2016 | Asia | China | Shanghai |  | • | • | • |  |  |  | • |  |  |  | • |  |  |  | • | Local | 1 |
| Wang et al 2017 | Asia | China | Hangzhou |  |  |  | • |  |  | Cancer | • | • | • |  |  |  |  |  |  | Local | 1 |
| Wang et al 2022 | Asia | China |  |  |  |  | • |  |  |  | • |  |  |  | • |  |  |  |  | Local | 2 |
| Wang et al 2023 | Asia | China | Guangdong |  | • |  |  |  |  |  | • |  |  |  | • | • | • |  | • | Local | 37 |
| Wang et al 2022 | Asia | China | Guizhou |  | • |  |  |  |  |  | • |  |  |  | • | • | • |  | • | Local | 75 |
| Wang, Dai et al 2020 | Asia | China |  |  |  |  |  | • |  | AMS |  |  |  |  | • |  |  |  |  | Local | 103 |
| Wang, Liu et al 2020 | Asia | China | Hubei |  | • |  |  |  |  |  | • |  |  |  | • | • |  |  |  | Reg'l | 2300 |
| Wang, Peng et al 2019 | Asia | China | Hangzhou |  |  |  | • |  |  | TPN | • |  |  |  | • |  |  |  |  | Local | 1 |
| Wang, Wang et al 2019 | Asia | China | Wendeng |  | • |  |  |  |  |  | • | • |  |  | • |  |  |  |  | Reg'l | 212 |
| Wang, Xuan et al 2020 | Asia | China |  | • | • |  |  |  |  |  | • | • |  |  |  |  |  |  | • | Local |  |
| Weeks et al 2017 | North America | Dominican Republic | Paraiso |  |  |  |  | • |  |  | • |  |  |  | • |  |  |  |  | Local | 1 |
| Wei et al 2019 | Asia | China | Rong county |  | • |  |  |  |  |  | • |  |  |  |  |  |  | • |  | Reg'l | 14 |
| Wei et al 2022 | Asia | China | Beijing |  |  |  | • |  |  |  | • |  |  |  |  |  |  |  |  | Local | 1 |
| Wei et al 2024 | Asia | China | Tibet |  |  |  | • |  |  | TB |  |  |  | • |  |  |  |  |  | Reg'l |  |
| Wei et al 2023 | Asia | China | Nanjing |  |  |  | • |  |  |  |  |  |  |  | • |  |  |  |  | Local | 3 |
| Winijkul et al 2021 | Asia | Thailand |  |  |  | • | • |  |  | AF | • |  |  |  |  | • | • | • |  | Org. | 27 |
| Wu et al 2016 | Asia | China | Beijing |  |  |  | • |  |  |  | • |  |  |  |  |  |  |  |  | Local | 1 |
| Wu et al 2021 | Asia | China | Fuyang |  |  |  | • |  |  |  |  |  |  |  | • |  |  |  |  | Local | 1 |
| Xi et al 2023 | Asia | China | Beijing |  |  |  | • |  |  |  | • |  |  |  |  |  |  |  |  | Local | 1 |
| Xia et al 2014 | Asia | China |  |  |  | • |  |  |  | TB |  | • |  |  | • | • | • |  | • | Nat'l |  |
| Xiao et al 2022 | Asia | China | Beijing |  |  |  | • |  |  |  | • |  |  |  |  |  |  |  |  | Local | 1 |
| Xin et al 2015 | Asia | China | Hangzhou |  |  | • |  |  |  |  | • | • |  |  |  |  |  |  |  | Local | 1 |
| Xu et al 2016 | Asia | China | Beijing |  |  |  | • |  |  |  | • |  |  |  | • |  |  |  |  | Local | 2 |
| Xu et al 2021 | Asia | China | Beijing |  |  |  | • |  |  |  | • |  |  |  | • |  |  |  |  | Local | 1 |
| Xu et al 2022 | Asia | China |  |  |  |  | • |  |  |  | • | • |  |  |  |  | • |  | • | Local | 5 |
| Yan et al 2017 | Africa | Zambia | Lusaka Province |  | • |  |  |  |  |  | • |  |  |  | • | • |  |  |  | Reg'l | 46 |
| Yan et al 2018 | Asia | China | Wuhan |  |  | • |  | • |  |  | • |  |  |  | • |  |  |  |  | Local | 1 |
| Yang et al 2013 | Asia | China |  |  |  |  | • |  |  |  | • |  |  |  |  | • |  |  |  | Local | 79 |
| Yang, Liao et al 2019 | Asia | China | Xiamen |  |  | • | • |  |  |  | • |  |  |  |  |  |  |  |  | Local | 1 |
| Yang, Lin et al 2019 | Asia | China | Xiamen |  |  |  | • |  |  |  | • |  |  |  |  |  |  |  |  | Local | 1 |
| Yue et al 2020 | Asia | China | Chengdu |  |  |  | • |  |  |  | • | • | • |  | • |  |  |  | • | Local | 1 |
| Yugandhar et al 2017 | Asia | India | Pondicherry |  |  |  | • |  |  |  | • |  |  |  |  |  |  |  |  | Local | 1 |
| Yulistiani et al 2023 | Asia | Indonesia | Surabaya |  |  |  | • |  |  |  | • |  |  |  |  |  |  |  |  | Local | 1 |
| Yusof et al 2023 | Asia | Thailand | Pathum-Thani |  |  | • | • |  |  |  | • |  |  |  |  |  |  | • |  | Local | 1 |
| Zaffar et al 2020 | Asia | Pakistan | Lahore |  |  |  | • |  |  |  | • |  |  |  |  |  |  |  |  | Local | 1 |
| Zaliska et al 2020 | Europe | Ukraine |  |  | • | • | • |  |  |  | • |  |  |  | • | • |  |  |  | Nat'l |  |
| Zaliska et al 2021 | Europe | Ukraine |  |  | • |  |  |  |  |  | • |  |  |  |  | • |  |  |  | Nat'l |  |
| Zaliska et al 2021 | Europe | Ukraine |  |  | • |  |  |  |  | CV disease | • |  |  |  |  | • |  |  |  | Nat'l |  |
| Zare et al 2024 | Asia | Iran | Tehran |  |  |  | • |  |  |  | • |  |  |  |  |  |  | • |  | Local | 1 |
| Zaver et al 2020 | North America | Honduras | San Jose |  | • |  |  |  |  |  |  | • |  |  |  |  |  |  |  | Local | 1 |
| Zhang et al 2021 | Asia | Sri Lanka | Southern Province |  |  | • | • |  |  |  | • |  |  |  |  |  |  |  |  | Local | 1 |
| Zhang et al 2020 | Asia | China |  |  |  | • | • |  |  |  | • |  |  |  | • |  |  |  |  | Local | 2 |
| Zhang et al 2017 | Asia | China |  |  |  |  | • |  |  |  | • |  |  |  | • |  |  |  |  | Local | 4 |
| Zhang et al 2016 | Asia | China | Taiyuan |  |  |  | • |  |  |  | • |  |  |  | • |  |  |  |  | Local | 1 |
| Zhang et al 2024 | Asia | China | Hangzhou |  |  |  | • |  |  | Intensive care | • |  | • |  | • |  |  |  |  | Local | 1 |
| Zhang et al 2023 | Asia | China | Jiaxing |  |  |  | • | • |  | COVID-19 | • |  |  |  | • |  |  |  |  | Local | 1 |
| Zhao et al 2022 | Asia | China | Beijing |  |  |  | • |  |  |  | • |  |  |  | • |  |  |  |  | Local | 1 |
| Zhao et al 2024 | Asia | China |  |  |  |  | • |  |  |  | • |  |  |  | • |  |  |  |  | Local | 930 |
| Zhou et al 2008 | Asia | China | Hangzhou |  |  |  | • |  |  |  | • |  |  |  |  |  |  |  |  | Local | 1 |
| Zhou et al 2023 | Asia | China | Chengdu | • |  |  |  |  |  |  | • |  |  |  |  |  |  |  |  | Reg'l | 6000 |
| Zhu et al 2017 | Asia | China | Guigang City |  |  | • | • |  |  |  | • |  |  |  | • |  |  |  |  | Local | 1 |
| Zhu et al 2024 | Asia | China | Hangzhou |  |  |  | • |  |  |  | • |  |  |  | • |  |  |  |  | Local | 1 |

Abbreviations: AMS = Antimicrobial stewardship, HIV = Human immunodeficiency virus, AF = Atrial fibrillation, TB = Tuberculosis, MDR-TB = Multiple drug-resistant tuberculosis, NICU = Neonatal intensive care unit, Tx = Transplant, Rheum = Rheumatology, PN = Parenteral nutrition, TPN = Total parenteral nutrition, A+E/ER = Accident and emergency/Emergency room, Paeds = Paediatric, Ophth. = Ophthalmology, Growth hormone def. = Growth hormone deficiency, Inflamm. bowel disease = Inflammatory bowel disease, Psych. telemed. = Psychiatry telemedicine, COVID-19 = Coronavirus disease 2019, CV disease = Cardiovascular disease, Org. = Organization, Nat’l = National, Reg’l = Regional, Comm. Pharm = Community pharmacy, Prim. Care = Primary care, Sec./tert. Care = Secondary/tertiary care, Presc. = Prescribing, Disp. = Dispensing, Admin. = Administration, Adh. = Adherence, EHR = Electronic health record, Pt. linkage = Patient linkage, PID = Patient identifier (e.g. a unique medical record number), Mgmt. level = Management level

**Supplementary table S2: Brief narrative descriptions of included studies**

| **Study** | **Identifier/link** | **Summary/notes** |
| --- | --- | --- |
| Ababneh et al 2020 | 10.1111/jphs.12356 | Comparison of medication/dispensing errors with electronic and paper prescriptions at 2 hospitals in Jordan. |
| Abbassi et al 2022 | 10.1016/j.pharma.2022.05.002 | CPOE for parenteral nutrition in neonatal intensive care. |
| Abdel-Qader et al 2020 | 10.1111/jphs.12376 | Investigating prescribing error rates, and concluded more training needed with the e-prescribing system for doctors. System name: Hakeem. |
| Abedian et al 2024 | 10.3233/SHTI240077 | Describes methods for developing standardized state-wide e-prescribing model to integrate with existing EMR. Uses an OpenEMR structure. |
| Abu Asab et al 2019 | 10.13140/RG.2.2.21716.01927 | Both local, pharmacy specific systems and nationwide EMR are mentioned. Local pharmacy systems span stock management to patient-level dispensing data. National data apparently is used to avoid duplicate dispensing and is linked to specific patients and longitudinal dispensed medicines records. System name: Hakeem. |
| Abu-Naser et al 2021 | 10.1177/0018578720985428 | Describes complete EMR system with prescription records. Study aimed to evaluate pharmacist-led interventions on prescribing error rates. |
| Afrash et al 2023 | 10.4103/jehp.jehp_263_22 | Implementation of a home-grown CPOE for chemotherapy prescribing in a tertiary hospital, study investigated error rates pre- and post-implementation. |
| Agh et al 2021 | 10.3389/fphar.2021.685696 | Survey of medication management practices across Europe. E-prescription availability in developing countries as per survey Yes: North Macedonia, Partly: Albania, Bosnia and Herzegovina, Not: Moldova. Given corroborating information about national availability of e-prescribing in North Macedonia, this has been included. Given only partial availability, lack of detail, and no corroborating information for Albania and Bosnia and Herzegovina, these have not been included. |
| Ahmed et al 2014 | 10.1587/transcom.E97.B.540 | Portable (briefcase sized) clinic system with electronic records stored for future consultations, and ability to generate e-prescriptions after remote tele-consultations. Used to reach small rural communities in Bangladesh (specifically two geographically distant districts near two major cities). |
| Al Meslamani et al 2021 | 10.1007/s40267-020-00806-x | 36 outpatient clinics from around Jordan were included - local electronic databases of prescriptions were queried and QT-interval prolonging drugs were investigated. |
| Al Meslamani et al 2022 | https://scholarworks.indianapolis.iu.edu/server/api/core/bitstreams/991729d3-f179-46fe-9de9-03f12c4bfc4b/content | Homegrown electronic prescribing system used in all public healthcare facilities in Jordan. Present study investigated medication errors related to e-prescribing within one emergency department. |
| Alipour et al 2024 | 10.1016/j.ijmedinf.2023.105282 | Nationwide Iranian e-prescribing systems (Iran Health Insurance Organization and Social Security Organization). Study in question was questionnaire-based and sought to understand patient experience with these systems at 5 teaching hospitals. |
| Allen et al 2007 | https://ebooks.iospress.nl/publication/11001 | OpenMRS implementation, use as described in Kenya where first used for management of PLHIV. Facilitates e-prescribing and medical records keeping. |
| Amaral et al 2022 | 10.1590/1516-3180.2021.0475.R3.03112021 | Sample of Sao Paolo physicians answered survey questions about telemedicine use during COVID pandemic. 72.6% reported using e-prescribing, while 61.4% also reported using electronic medical records. Specific systems not reported. |
| Amir et al 2022 | 10.1177/27550834221119689 | Article describing the experience and process of one site in a tertiary hospital implementing a CPOE system. |
| Aneja et al 2022 | 10.4103/jmhhb.jmhhb_191_22 | Describes use of WhatsApp messenger and landline phones to consult with patients remotely. Unclear how the prescribing system works but describes prescription being 'generated' and saving time for patient to travel to consultations. |
| Arabian et al 2024 | 10.3389/fmed.2024.1385256 | Survey of patients to understand their perspectives on Iran's nationwide e-prescribing system. One patient describes a perception that the system will use a 'national code', presumed to be a patient identifier, but this is not elaborated upon further |
| Asefa et al 2016 | 10.1377/hlthaff.2016.1294 | Editorial/comment describing national programme in Namibia using electronic dispensing records to identify people who fail to collect their ART, aiming to improve adherence. |
| Aslam et al 2023 | 10.4103/1995-7645.370148 | Study using data from an electronic data management system (Electronic Nominal Record System) and another pharmacovigilance database (Active Tuberculosis Drug Safety Monitoring and Management), both specifically for TB. Sought to investigate and quantify ADRs among individuals receiving anti-TB medicines. |
| Atif et al 2022 | 10.3389/fmed.2021.790718 | Study using data from an electronic data management system (Electronic Nominal Record System), specifically for TB. Sought to investigate and quantify adverse drug reactions among individuals receiving anti-TB medicines in one clinical unit. |
| Atthobari et al 2010 | https://onlinelibrary.wiley.com/doi/epdf/10.1002/pds.2019?saml_referrer | E-prescribing implemented in Indonesian clinic, and found this increased generic prescribing rates significantly. |
| Awad et al 2018 | 10.1186/s12879-018-3512-z | Lebanese hospital correlating antifungal consumption from pharmacy records with types of fungal infections isolated in inpatients. |
| Ayala-Estrada et al 2016 | 10.1159/000451040 | EasyPod growth hormone delivery system, tracks adherence and drug administration for a small number of patients nationally with growth hormone deficiency. Produces data on adherence to this single drug. |
| Azim et al 2019 | 10.1186/s12913-019-4420-7 | Non-governmental tertiary hospitals in Pakistan, one with e-prescribing, the other without, measured medication errors in cancer inpatients. Mentions highly upgraded CPOE system with clinical decision support system. There also seem to be records of dispensing and administration, but unclear if electronic system used at point-of-care. |
| Aziz et al 2015 | 10.1177/183335831504400303 | Chemotherapy-specific CPOE implemented in tertiary cancer centre. Integrated with wider hospital HIS, but HIS not described. Describes improvements in error rates and cost-savings. |
| Badin et al 2023 | 10.1371/journal.pone.0280891 | Used data from a system called AGHU (Application for Management of University Hospitals), which contains prescriptions and other clinical information, to study predictors of mortality in illness caused by COVID. This study only reports use in one hospital, but this system is implemented in public hospitals nationally. |
| Baesso et al 2022 | 10.7399/fh.13039 | EMR system described within the context of one hospital. Reportedly provided by TASY (Philips). Pharmacovigilance study looking at adverse responses to a select group of medicines in a hospital setting. |
| Bahmani et al 2022 | 10.18502/jimc.v5i4.11347 | Nationwide Iranian e-prescribing system. Article presents ethical and legal concerns around confidentiality and data-sharing. |
| Barigela et al 2021 | 10.4103/ijcm.IJCM_304_20 | Primary health clinics surveyed as to availability of a variety of electronic systems. 2 sites have records of patient medicines, but unclear where these sites are. No sites had e-prescribing. No other EMM systems included in survey. |
| Bonella et al 2016 | 10.1016/j.bjid.2016.07.002 | Trial of an alert on third day of intravenous antibiotics and auto-discontinuation on 5th day. |
| Bouh et al 2024 | 10.1177/20552076241281626 | Nationwide health information system (shared health record), amalgamated from the District Health Information Software DHIS2. Built by adapting OpenMRS. |
| Bouraghi et al 2024 | 10.1186/s12913-024-11144-3 | Nationwide Iranian e-prescribing system. Study used survey to explore physician opinions on this system. |
| Buawangpong et al 2020 | 10.1186/s12875-020-01183-0 | Thai primary care clinics within a hospital with EMRs documenting medications prescribed. Unclear if specifically e-prescribing. |
| Campos et al 2021 | 10.1590/1413-81232021269.19462020 | Pharmacovigilance study using region-wide data on thalidomide dispensing provided by the regional public health service (part of the national health service termed the SUS). Not obvious from writing whether the reported pharmaceutical care management system is implemented nationally. However, does also mention SICLOM and SNGPC as national medicine dispensing/distribution databases. |
| Carmona et al 2012 | 10.1590/S0100-879X2012007500005 | Describes alerting system/antimicrobial stewardship using e-prescribing to decrease rates of vancomycin-resistant entrerococcus after an outbreak. |
| Cassiani et al 2003 | 10.1590/S0080-62342003000400006 | E-prescribing introduced at this tertiary university hospital in 1998 in a phased manner. This article investigates sources of medication errors in the (as of 2003) hybrid written and electronic system. |
| Chai et al 2023 | 10.2147/JMDH.S432522 | Randomized controlled trial to study impact of changing medicines reconciliation processes. Describes an electronic medical records system with medicines data and computerized order entry. |
| Chang et al 2020 | 10.1016/j.jiph.2020.05.027 | Describes HIS which contains info on prescriptions, unclear if electronically prescribed. Intervention intended to reduce inappropriate antibiotic prescribing by physicians. |
| Chen et al 2019 | 10.1016/j.sapharm.2019.02.006 | Investigation of new medication error monitoring system. "MEMS" system described is for manual input or medication errors data, however e-prescribing and dispensing (barcode) systems are also described |
| Chen et al 2020 | 10.1007/s43441-019-00027-z | Government military hospital EMR data on drugs administered to inpatients used to investigate a new adverse drug reaction monitor/alarm system. |
| Chen et al 2022 | 10.1007/s40121-022-00608-w | Observational study to explore relationships between antimicrobial use and resistance in a single hospital. Used electronic medical records. |
| Chinwong et al 2021 | 10.1016/j.sapharm.2021.01.003 | Retrospective study of cardiovascular outcomes and medication adherence at a single hospital site. Used 'electronic hospital database' data and reports existence of what sound like dispensing data. |
| Colletti Junior et al 2018 | 10.5935/0103-507X.20180057 | Survey sent to ICU physicians around Brazil re: EMR/e-prescribing systems. Most used EMRs and worked in private hospitals. Notably 66.7% of survey respondents who did not use an electronic system were in state hospitals rather than private ones. 189 of 204 sites reported use of EMR and e-prescribing. |
| Connolly et al 2015 | 10.1016/j.jval.2015.09.2816 | Abstract only. Medicines cost data from regional EMR database in Shanghai used to evaluate cost impact of providing hormone replacement therapy to post-menopausal women. |
| Corbell et al 2012 | 10.1002/pds.2252 | Describes probablistic method of record linkage for three databases of information re: HIV patients in Namibia. Contains dispensing records, blood results, and important demographic variables. May only contain information about anti-retroviral medicines rather than all drugs. Created newly linked dataset for PLHIV on anti-retroviral therapy. Previously no shared unique identifier across data sources. Settings included but were not not limited to hospitals. |
| Costa et al 2004 | 10.1016/j.ijmedinf.2004.02.006 | Data from one hospital with EMR, prescribing, and administration records. Description of implementing newly added drug distribution system. Patients have unique barcodes. |
| Costa et al 2021 | 10.15649/cuidarte.1332 | Survey of nursing staff in an intesive care unit to ascertain perceptions about EMR in use. System not described in any more detail but medication data clearly included and alludes to point-of-care medicine administration system. |
| Cueva et al 2020 | 10.1155/2020/5423080 | Ecuadorian private outpatient clinic - describes EMR with data on antibiotics prescribed to patients with PID. No specific mention of eprescribing or dispensing systems. |
| Curtarelli et al 2019 | 10.1590/1677-5449.004018 | Hospital inpatient electronic records queried to investigate VTE prophylaxis. |
| Cury et al 2019 | 10.2147/JIR.S190929 | Used EMR of inflammatory bowel disease management (including drugs used) to analyse time course/management/natural history of illness. Standardised questionnaire filled in for new inflammatory bowel disease patients at first consult, then input into database. |
| Custodio et al 2023 | 10.1016/j.bjpt.2023.100553 | Observational study looking retrospectively at outcomes for people with spinal disorders at a single centre. Describes a national (SUS managed) electronic record system which has data on prescriptions. Mentions DATASUS in passing which is the governmental SUS department responsible for health data management. |
| da Rocha et al 2021 | 10.1177/10781552211017650 | E-prescribing system in place at tertiary university hospital, specifically for antineoplastic regimes. |
| Daher et al 2020 | 10.1055/s-0040-1721424 | Neonatal unit developed neonate specific e-prescribing system. Found this decreased errors and improved efficiency. Also mentioned faster documentation in medical records. |
| Dao et al 2024 | 10.1080/20523211.2024.2381099 | Retrospective analysis of EMR data for people with heart failure at one hospital site to understand economic costs. |
| Darawad et al 2019 | 10.1111/nhs.12620 | National implementation of EMR in 2009, important aspect is barcode medicines administration and medicine administration record. Article used surveys to assess satisfaction among nurses with the barcode administration system. No clear mention of e-prescribing or other elements of electronic health system used. |
| De et al 2021 | 10.4103/ijd.IJD_771_20 | Dermatology clinic in India reviewed prescription records in EMRs to investigate novel antihistamine usage. |
| De Oliveira et al 2020 | https://doi.org/10.1590/0102-311X00060520 | Dispensing records for a region of Brazil. Queried to investigate prescribing trends of psychotropic drugs. |
| de Souza et al 2016 | 10.1186/s12887-016-0551-8 | Local EMR for patients on NICU at a specialist hospital. Unclear if also e-prescribing. Investigated prescribing patterns in neonates, especially off-label and potentially harmful medicines |
| Dethlefs et al 2019 | 10.1016/j.diabres.2019.01.010 | Rural health clinics in two geographically distant areas of DR - no clear data sharing between them. Data manually inputted to an Access database called "Salud". Dashboards and automated reports used in part to monitor number of distinct active patients and medication dispensing and stock management. |
| Diaz et al 2020 | 10.1016/j.rcp.2018.05.001 | Data managed by MC21 (a private business management company that serves insurers). Contains information on outpatient medicines prescribed and dispensed under the remit of affiliated insurers (pharmacy benefit management system). Population 2.3m covered by affiliated insurers (~5% of population). Also contains demographic information. Online clinical alert system for drug interactions/contraindications etc forms part of system. Investigated risks associated with tricyclic antidepressant prescribing. |
| Do et al 2023 | 10.1016/S1473-3099(23)00125-1 | Cluster randomized controlled trial to study impact of point-of-care c-reactive protein testing to help targeting antibiotic prescribing. Used data from primary care providers that maintainted electronic record databases. Describes linkage for some patients using a health insurance number. |
| Donneyong et al 2019 | 10.1111/bcpt.13136 | National insurance claims database with individual IDs and prescription claims. Reports that based on its structure, it could easily be adapted/used for drug utilization research. |
| Dorairaj et al 2018 | 10.1161/CIRCULATIONAHA.118.038192 | Assesses efficacy of an mhealth intervention for blood pressure mangement - unclear if deployed beyond trial end. However, paper also mentions that the Indian government plans to roll-out EMR system in the public healthcare system soon at the time of writing. Not clear if previous and current medication records are based on e.g. prescribed/self-reported history/dispensed medicines. |
| Doubova et al 2014 | 10.1186/1471-2393-14-168 | Describes The Mexican Institute of Social Security (IMSS) which provides care for 47m Mexican nationals, and "uses EMR for the routine provision of healthcare". Four sites in Mexico City included in analysis to investigate quality of ante-natal care, and data included records of prescriptions e.g. for folic acid and metronidazole. |
| Du et al 2023 | 10.3389/fpsyt.2023.1195298 | Descriptive study exploring prescriptions issued through a psychiatric telemedicine/internet hospital set up during COVID pandemic. |
| Duangsong et al 2022 | 10.11909/j.issn.1671-5411.2022.07.001 | Study of potentially inappropriate medicines in heart failure patients. Data from two different geographically distant public hospitals in Thailand. EMMS only superficially described. Same system as Jenghua 2020 and 2022. |
| Elhawary et al 2022 | 10.1007/s40264-022-01219-7 | Single site refugee hospital in Cairo with a home-grown electronic prescribing system. Present article is abstract only, but compares pre- and post-implementation medication errors. |
| Elias et al 2017 | 10.1007/s11255-017-1650-7 | Investigation of secondary hyperparathyroidism in chronic kidney disease patients in one hospital, via data extracted from electronic charts. Mention of prescription records within this database, but unclear if these were e-prescriptions or not. |
| Fan et al 2019 | 10.1080/14740338.2019.1645832 | Chindren's hospital in Beijing - EMR electronic prescription records used to investigate medicines which may cause anaemia. |
| Fatima et al 2024 | 10.1136/bmjoq-2023-002527 | Single site tertiary hospital implemented CPOE. Still use paper documentation as well at time of writing. Study to audit medicines reconciliation and compare paper system with CPOE. |
| Ferracini et al 2016 | 10.1186/s40360-016-0082-9 | Investigation of positive deviance (small changes on individual scale) to improve patient care etc. Measured based on medication errors - at least some of the prescriptions were electronic. Medicine error reporting system including gradual increase in proportion of prescriptions which were electronically prescribed. Also described voluntary/self report medication error system. |
| Fraser et al 2013 | 10.3233/978-1-61499-289-9-627 | This article describes the development of the partners in health (PIH) EMR and Open-MRS(TB), and broadly characterizes their deployment in various countries and settings. At the time this paper was written, OpenMRS-TB was deployed in Haiti, Pakistan, Nepal, Tajikistan, Indonesia, Kenya and Botswana. OpenMRS was reportedly first deployed in 2006 and was by 2013 used in over 50 developing countries primarily to support HIV treatment and primary care. System supports medicines management (prescribing, dispensing and forecasting e.g. stock use). Linkage intranationally but not internationally. |
| Fu et al 2019 | 10.1002/pds.5064 | Primary care electronic medical records queried to investigate potentially inappropriate medicines. While this research did not have access to patient-level data, patient IDs and linkage does reportedly exist. |
| Fumis et al 2012 | 10.1186/cc11124 | Abstract only. ICU in Sao Paolo implemented CPOE system with barcode administration. Reports results of satisfaction survey. |
| Ghadieh et al 2015 | 10.1016/j.vaccine.2015.07.050 | EMR in lebanon 'family medicine' clinic (primary healthcare physician clinic at a hospital) - included data on demographics, medical history, and whether patients had received pneumococcal vaccine. Unclear if contained data on other medicines. |
| Gimenes et al 2006 | https://www.researchgate.net/publication/28130961_Electronic_prescription_as_contributing_factor_for_hospitalized_patients'_safety | Brazilian tertiary hospital, CPOE and handwritten prescriptions used. Compared error rates and sources of errors. |
| Gimenes et al 2019 | 10.1371/journal.pone.0220248 | Investigation of patients with drug-drug interationc via nasogastric tubes. 6 hospitals in Brazil participated, of which three (unclear which ones) use e-prescribing. |
| Gonzalez-Perez et al 2019 | https://www.medigraphic.com/cgi-bin/new/resumenI.cgi?IDARTICULO=93108 | EMR system with records of medicines available. Investigated cost of schizophrenia at this single primary care (family medicine) site. |
| Goodman-Meza et al 2021 | 10.1016/S2468-2667(20)30260-7 | Prescription data from national database used to investigate opioid usage. Includes public and private pharmacy dispensing records. No mention of whether these included hospital and/or community pharmacies. |
| Green et al 2015 | 10.1186/s13063-015-0558-8 | Implementation of openMRS (AMPATH MRS/AMRS) in western Kenya, specific personnel employed to enter handwritten encounter information into the EMR, including prescribed medicines. |
| Gu et al 2023 | 10.1016/j.archger.2023.104939 | Study of potentially inappropriate medicines prescribed for chronic conditions at a single hospital site. Reports presence of both dispensing and prescribing data but no explicit report of point of care electronic prescribing or dispensing system. |
| Guan et al 2021 | 10.1097/PTS.0000000000000753 | Study using electronic prescription records at a single hospital site to investigate association between physician workload and prescription quality. Found higher workload associated with increased risk of inappropriate prescribing. |
| Guidoni et al 2014 | 10.1007/s11239-013-1030-9 | Used electronic data to investigating demographics, past medical history, and lab results of patients given warfarin as an inpatient at this tertiary hospital. |
| Guidoni et al 2016 | 10.1007/s11096-016-0336-z | Data from e-prescribing system database local to this large hospital. Diagnoses recorded within related hospital system. No mention of dispensing or administration records/systems. |
| Guo et al 2021 | 10.1370/afm.2686 | Uses data from Beijing's public medical insurance database, reports electronic medical records system but no description of this. Studied diabetes medicines and found decreasing complexity and costs of treatment over time. |
| Guo et al 2021 | 10.1097/MD.0000000000026912 | Uses EMR data to study antipsychotic prescribing patterns at a single hospital site. |
| Hampapur et al 2017 | https://www.aacc.org/-/media/Files/Meetings-and-Events/Annual-Meeting/2017/AACC17_AM_B006B028.pdf?la=en&hash=20A65B1FEA7738799B2A30CE290CD8B973F351ED | Abstract only. This hospital in Mumbai reportedly has had a fully paperless operation since 2013. The specific medicines related systems mentioned in this abstract are EMR, electronic prescribing (within broader CPOE), and dispensing records. |
| Haskew et al 2015 | 10.1016/j.ijmedinf.2015.01.005 | OpenMRS based system deployed in rural Kenya for mother and child care. Information on antiretroviral and tuberculosis treatment, and screeining for malaria and hypertension. |
| He et al 2023 | 10.1136/bmjopen-2022-068545 | Used EMR data from 37 rural primary health care hospitals to study antibiotic prescribing trends. |
| Hernandez-Avila et al 2015 | 10.1016/j.jval.2015.09.108 | Extraction of data, including medicines data, regarding diabetes care from primary care EMR covering all primary care clinics in a region of Mexico (full coverage by 2013, first roll-out 2005). |
| Hinojosa-Amaya et al 2016 | 10.1111/jep.12535 | Local EMR at a large hospital in northern Mexico, study assessing medication errorss with e-prescribing vs written. In results tables mentions ability to discern between errors made at different stages of drug delivery process, and presence of a 'fully electronic system'. |
| Hitti et al 2017 | 10.5811/westjem.2017.6.32037 | E-prescribing system deployed in an emergency department. Error rates compared pre- and post roll-out. |
| Hoagland et al 2020 | 10.1016/j.bjid.2020.05.004 | Unitaid funded project delivering e-prescribed pre-exposure prophylaxis to at-risk patients via a hospital-led clinic in Rio de Janeiro. |
| Hossain et al 2019 | 10.1089/tmj.2018.0081 | Portable health system allowing e-consultation and e-prescrition, integrated with EMR. Delivered to rural communities in Bangladesh. |
| Hua 2016 | 10.1136/ejhpharm-2015-000731 | University hospital in China trialling new feature for monitoring rational antibiotic use using data from CPOE and EMR systems. |
| Hua et al 2020 | 10.1016/j.japh.2020.04.006 | Describes electronic pharmacy system at hospital in Wuhan - mentions e-prescribing and that ~20,000 these electronic orders have been reviewed by the pharmacy. |
| Huang et al 2020 | 10.1007/s11096-020-01031-2 | Two hospital sites in Hunan province, describes EMR with many data, including pharmacotherapies that had been prescribed. Unclear nature of medicines data, e.g. if e-prescribing/dispensing records. |
| Idrees et al 2018 | <https://www.scienceopen.com/document?vid=7028bda4-6520-4763-844e-bc5517d89ba1> | Abstract only. Rural clinic in Honduras implementing electronic pharmacy management system with dispensing and stock data. Studied impact of system on prescribing patterns for communicable diseases. Same system as In Zaver 2020. |
| Jazayeri et al 2003 |  | EMR prototype deployed in impoverished rural areas of Haiti for the purpose of HIV monitoring and management - has e-prescribing functionality. Describes plans to scale-up in the future. |
| Jebraeily et al 2024 | 10.1186/s12911-024-02687-w | Physicians interviewed to perform SWOT analysis for e-prescribing system in place nationally in Iranian hospitals since 2019. Technological infrastructure recognised as pivotal both as a strength and potential weakness. |
| Jeddi et al 2024 | 10.48307/iahsj.2024.414098.1054 | Questionnaire based study of physicians' use of different e-prescribing features and functionalities in outpatient settings, where e-prescribing reportedly rolled out over 5 years prior to article publication. Reports several means used to identify patients but no mention of unique patient ID number |
| Jenghua et al 2020 | 10.1111/ijcp.13878 | Two hospital sites in different provinces, investigated potentially inappropriate medicines in heart failure from electronic medical databases which contained information on prescriptions at inpatient and outpatient departments. Same system as Jenghua 2022 and Duangsong et al 2022. |
| Jenghua et al 2022 | 10.18549/PharmPract.2022.1.2487 | Two hospital sites in different provinces, investigated potentially inappropriate medicines in heart failure from electronic medical databases which contained information on prescriptions at inpatient and outpatient departments. Same system as Jenghua 2020 and Duangsong et al 2022. |
| Jiang et al 2024 | 10.1007/s00262-024-03829-9 | Study using EMR data to investigate factors associated with survival in small cell lung cancer. Included data on chemotherapy regimen and various other clinical and laboratory measures. |
| Jiao et al 2020 | 10.1155/2020/8828738 | Comprehensive e-health application designed and deployed in several use-cases mentioned at the end of the paper - unclear if entirety of the system is utilised by all of these consumers/users of the system (describes using the system for certain workflows/aspects of service delivery like outpatient follow-up, but not necessarily all aspects). Reportedly has a role in e-prescribing, ordering and recording biomedical tests/results, and interface for video or in-person consultations. Software system used widely in Sichuan regional hospitals ("dozens", however only 8 explicitly mentioned). |
| Jokar et al 2023 | 10.1186/s12871-023-02335-2 | Study reporting development and implementation of anaesthesia module for existing EMR and e-prescribing system at one hospital site. |
| Joseph et al 2020 | 10.1186/s43044-020-00088-8 | Extracted data from an EMR. Contains records of medicines given during hospital stay; which are related to billing information generated at discharge. |
| Joshi et al 2024 | 10.15605/jafes.039.01.15 | EMR data used to study acute coronary syndrome risk in patient with type 2 diabetes. |
| Kadayaprath et al 2024 | 10.4103/ijc.ijc_565_21 | Retrospective study using EMR e-prescription data to audit breast cancer treatment at a single centre. |
| Kandasamy et al 2021 | 10.1111/ijcp.14489 | Observational study of prescribing errors at one private hospital site. 4% of prescriptions were computerized. |
| Karajizadeh et al 2021 | 10.5812/jhealthscope.104607. | CPOE used for 'all orders' since 2015 at a teaching hospital. |
| Karajizadeh et al 2023 | 10.1016/j.ijmedinf.2023.105135 | Studied implementation of new high-risk drug-drug interaction alerting system in intensive care setting within an existing CPOE and EMR system. Found increased rates of adherence to alerts. |
| Kassem et al 2021 | 10.1016/j.jsps.2021.08.009 | Sampled community pharmacy prescriptions with errors from various regions in Egypt. Around 20% were computerized prescriptions. Found differences in types of errors between handwritten and e-prescriptions. |
| Kazemi et al 2010 | 10.2196/jmir.1284 | All 15 university affiliated hospitals in this province use the same clinical information system with CPOE. Investigated medication errors in a neonatal ward, comparing CPOE with nurse led order entry with physician calidation/verification. Paper-based medicines administration record. Improvements in number of errors was noted, and increased rate of heeded/complied-with warnings. |
| Kazemi et al 2011 | 10.1007/s10916-009-9338-x | Region in which all university affiliated hospitals have this HIS, which is described in detail in this paper. Includes EMR, CPOE, medicines administration records and e-prescribing. Study evaluates effect of roll-out in a neonatal unit at one of these hospitals in three stages (without CPOE, with CPOE, and with CPOE+CDS feature). |
| Kenwy et al 2019 | 10.1016/j.ijmedinf.2019.04.005 | Specialist hospital outpatient clinic investgated medication errors before and after roll-out of e-prescribing system. |
| Khammarnia et al 2017 | 10.14196/mjiri.31.69 | CPOE trialled in intensive care ward in tertiary centre in Shiraz, Iran. |
| Khammarnia et al 2018 | 10.5812/semj.66301 | Abstract only. Investigated medication errors before and after implementation of new CPOE on mobile devices. Found significant drop in overall prescription errors. |
| Khan et al 2018 | 10.1002/rth2.12125 | Abstract only. CPOE data in a Karachi hospital investigated for rivaroxaban prescribing trends to assess safety. |
| Khan et al 2020 | 10.1007/s40801-020-00211-w | Both primary and secondary care electronic records introduced to dozens of clinics nationally in 2016. Contains records of prescriptions. Not mentioned if e-prescribing functionality present. |
| Khan et al 2021 | 10.1080/14779072.2021.1941872 | Studied coronary artery disease management using EMR data from software administered by HealthPlix Technologies PRV which reportedly fulfills day-to-day operational needs of 12 medical specialties across 150+ cities in 20 states in India. |
| Khowaja et al 2008 | 10.2147/tcrm.s2646 | CPOE system in place, investigated for sources of medication errors and reasons for under/non-reporting. Describes system having a role in reducing errors at the prescription, dispensing, and administration stage of drug delivery. |
| Killian et al 2019 | 10.1145/3292500.3330777 | Deep learning model trained on directly observed therapy (DOT) data from a city in India, allowed prediction of those who will miss doses. |
| Kimaiyo et al 2005 | 10.1016/j.ijmedinf.2005.03.002 | OpenMRS implementation for HIV management at various centres in Kenya. Related to Liu et al 2013 - this described the older system which uses handwritten data input to database on a weekly basis. Includes data on anti-retrovirals. |
| Koopmans et al 2018 | 10.1016/j.ijid.2018.05.020 | EMMS in a hospital pharmacy, with data used to study antimicrobial prescribing trends in paediatric patients. System provided by JAC Medicines Management software since 2000. Hospital does not have access to the e-prescribing module. Quote from article: Although electronic prescribing is not available, electronic pharmacy stock management tools are widely used in public sector South African healthcare facilities. |
| Kosuma et al 2019 | 10.1177/1179546819855656 | Database of medical and pharmacy claims at a Thai hospital, used to study statin use prevalence and predictors. |
| Kruger et al 2021 | 10.1080/21548331.2021.1889213 | Purpose-built web app for antimicrobial stewardship through point-prevalence monitoring. Development towards real-time data collection and since used in nation-wide studies. Same system used in Skosana 2021. |
| Kruse et al 2022 | 10.17159/2078-5151/SAJS3670 | Describes fully computerized prescribing system at one quarternary hospital. Compares error rates and types with different prescribing systems at 3 other hospitals, finding more than increased technological complexity alone is required to reduce medication error rates, emphasizing human factors engineering and persistent critical assessment. |
| Kruse et al 2024 | 10.4102/safp.v66i1.5845 | As in another Kruse et al (2022) paper, describes fully computerized prescribing system at one quarternary hospital. Compares error rates and types with different prescribing systems at 3 other hospitals, then develops an error reduction strategy. |
| Kwiatkowska et al 2020 | 10.1186/s12913-020-05308-0 | Studied antimicrobial use using electronic prescription records. Lots of commercial pharmacy use was observed. Some village clinics have e-records entered at point-of-care, weekly, or yearly as part of prescription audits, but township health centres are obliged to enter medical insurance ID at the point-of-care to get reimbursed. While this study was about antimicrobials, all medicines are reportedly managed this way. |
| Lakkis et al 2015 | 10.1111/jep.12386 | Quality of diabetes care investigated using medical therapies data within EMR/insurance records. |
| Lan et al 2021 | 10.5414/CP203861 | Retrospective study of antivirals for COVID-19 in hospitalized patients. Used EMR data from a single hospital site. |
| Larasati et al 2024 | 10.21315/mjms2024.31.4.13 | Uses EMR data from single hospital site to study drug-related problems among patients with type 2 diabetes. |
| Layton et al 2005 | 10.1016/S1098-3015(10)67743-1 | Abstract only. Outpatient electronic prescription records investigated at single teaching hospital for prescribing pattern changes around the time of marketing policy changes. |
| Le et al 2024 | 10.1155/2024/5354295 | EMR records from a single site used to retrospectively study hypertension related to apatinib use. |
| Leal et al 2020 | 10.36416/1806-3756/e20180325 | Hospital EMR with CPOE. Looked into changes in venous thromboembolism prophlaxis after implementation of a new protocol. |
| Lestari et al 2024 | 10.1016/j.lansea.2023.100294 | Used data from national electronic program database system (e-TB manager) to study drug-resistant tuberculosis management. |
| Li et al 2014 | 10.1002/pds.3701 | Abstract only. West China hospital, assessed EMR prescription records for discharged diabetic patients. No mention specifically of e-prescribing. Investigated different classes of anti-diabetic medicines prescribed while inpatients. |
| Li, Foy et al 2018 | 10.1002/jac5.1059 | Abstract only. Newly implemented (2016) electronic dispensing and stock management system. Studied how non-electronic prescribing trends changed after deployment. Rural clinic, unclear location. IV fluids included so uncertain whether setting is primary/secondary care. |
| Li, Sheyu et al 2018 | 10.1111/jebm.12291 | Hospital EMR with order entry capability. Used to characterise/describe diabetes inpatients at baseline for a future study. |
| Li, Zhang et al 2013 | 10.1007/s10916-013-9937-4 | Discusses local 'comprehensive EMR' at hospital. Alludes to nationwide distribution of similar systems, but specifically describes them as not linked/individually designed by different hospitals. Used in this case for studying antibiotic stewardship. |
| Li, Zhou et al 2013 | 10.1016/j.sapharm.2013.06.005 | Letter to the editor describing a local e-prescribing system - alludes to nationwide adoption of e-prescribing, but is not clear about exactly how common/widespread it is. Talks about addition of current diagnosis on prescriptions to help pharmacists assess appropriateness of prescription. |
| Liang et al 2018 | 10.2196/formative.9658 | Regionally deployed EMR system that allows real-time updates on drug stock, and interface for identifying drug-drug interactions when prescribing (linked to Uganda National Drug Authority drug reactions reporting system). Unique identifiers used, but are not consistent across sites. |
| Liang et al 2021 | 10.2196/24813 | Presents data on rates of adoption of EMR and health information systems from US and Chinese national surveys (ONC data briefs, HIMSS annual surveys and CHIMS). Reports 85% adoption in Chinese hospitals as of 2018. Contrasts drivers of and challenges to adoption in different countries. Does not describe EMMS in detail. |
| Lim et al 2021 | 10.1016/j.jinf.2020.10.040 | Uses electronic health records data from single hospital site to study antimicrobial prescriptions and resistance. |
| Lima et al 2014 | 10.1590/S1984-82502014000300016 | Study reporting development of a feature that shows basic vancomycin pharmacokinetics at the point of e-prescribing, with the intention of improving dose/rate selection for body weight and creatinine clearance. Tertiary hospital in Rio. |
| Lima et al 2018 | 10.1590/0102-311X00009617. | Same system mentioned in Madruga et al 2018 paper - national HIV/AIDS monitoring system. Describes linking multiple parts of this system using probablistic methods, but only using a subset from Rio de Janeiro. Does not go into depth about data content of each database. SICLOM, SINAN-AIDS, SISCEL and SIM are the original unlinked databases, containing various information from mortality data to lab results, and the SICLOM electronic dispensing system. |
| Limsomwong et al 2023 | 10.3389/fonc.2023.1138169 | Uses data from a health information system at single hospital site to study opioid use rates in palliative care contexts. |
| Lin et al 2018 | 10.1136/bmjopen-2017-019698 | Regional health information system with linked data in Eastern China. Used EMR data to study cardiovascular care and outcomes. |
| Liu et al 2013 | 10.1111/jphs.12002 | OpenMRS implementation (AMPATH) for HIV management at various centres in Kenya. 23 sites contribute prescription data to prescribing/dispensed medicines database, and all have AMPATH MRS, but only 8 sites have point-of-care e-prescription entry. At the time of wrting/inception, some pharmacies used electronic systems and allowed for e-precriptions, others still used handwritten logs. New OpenMRS modules developed to create a dispensing record database and assist with ART adherence monitoring. Also dealt with stock management. |
| Liu et al 2015 | 10.1371/journal.pmed.1001876 | Use of different adherence improvement approaches, including e.g. an electronic pillbox, investigated in cluster randomized trial. |
| Liu et al 2016 | 10.1097/MD.0000000000003965 | Investigated 'public reporting of healthcare performance' - used e-prescriptions from 20 outpatient clinics in one region. |
| Liu et al 2022 | 10.1136/ejhpharm-2020-002356 | Retrospective study of drug-related problems among Parkinson's disease patients at single hospital site. Only superficial description of EMMS. |
| Lobo et al 2017 | 10.17058/reci.v7i1.7279 | CPOE in a Brazilian hospital, analysis of records used to evaluate new antimicrobial optimization programme. |
| Lyu et al 2022 | 10.3389/fmed.2022.936234 | Retrospective analysis of EMR data for people with receiving Chinese herbal medicine for migraine at a single hospital site. |
| Maarsingh et al 2022 | 10.3389/frhs.2022.960427 | Implemented a purpose-built EMR system at a series of 1-day medical camps in 5 rural villages. Used battery-powered hardware due to lack of electricity. Systems developed by Infotech Solutions in 2018 using an open-source web platform (TikiWiki), an open-source database platform (MySQL), and an open-source web server (Apache). Found significant productivity improvement with electronic vs paper-based system. |
| Mabirizi et al 2018 | 10.9745/GHSP-D-18-00157 | Nationwide gradual implementation of electronic pharmacy/medicine stock data for PLHIV/ART. Data at both a national (stock) level but also pharmacy-specific level including electronic dispensing functionality. Initially the e-dispensing tool (EDT) was used at hospitals and 'high volume health centres' but later a mobile EDT was also used at rural primary care centres. |
| Madruga et al 2018 | 10.1590/1413-812320182311.24742016 | SICLOM Braziilian national dispensing system for monitoring patients on antiretroviral medicines - has individual patient level data on drugs prescribed and dispensed. This study looked at data on patients registered at two pharmacies in tertiary hospitals. |
| Maisso et al 2009 | 10.1590/S0102-311X2009000200009 | Precription errors analysed from 4 hospitals in 3 different states in Brazil - one hospital had electronic prescriptions. |
| Maksymovych et al 2020 | 10.1016/j.jval.2020.04.826 | Abstract only. Ukraine implemented e-prescribing system in 2019 for 23 essential medicines. Almost 11 million prescriptions have apparently been captured via this data. Unclear if available data are linked to patients, but some demographics available including e.g. urban/rural. Only 36.5% of pharmacies nationwide were using this e-prescribing system. |
| Mashoka et al 2019 | 10.1016/j.afjem.2019.07.002 | Describes deployment of EMR 'EDIS' in emergency medicine department. Has records of all medications and orders, as well as labs and other patient information. Also describes a system 'eFMS' which has been developed under government oversight and deployed at some hospitals, with roll-out planned for all hospitals in the country. However, this system is not described, and it is unclear if it would gether medicines information/have any EMM system. |
| Messou et al 2011 | 10.1097/QAI.0b013e3182084b5a | 3 HIV clinics in 'economic capital' of Cote D'Ivoire which have 'computerised databases' of prescriptions. Unclear if these are e-prescriptions or computerized/scanned copies of hand-written ones, or part of a wider EMR. |
| Miao et al 2020 | 10.1097/MD.0000000000018714 | Compares antibiotic use in several different regional hospitals, extracted from electronic records. |
| Miao et al 2020 | 10.1097/MD.0000000000018714 | Studies inappropriate antibiotic prescribing in different types of hospital using EMR data. Does not clarify whether there is data sharing across sites or if this has been pooled manually. Unclear where each of the included hospitals is located. |
| Migowa et al 2018 | 10.1177/2042098618781520 | Studied change in medication errors using a voice recognition system to generate prescriptions. |
| Miranda-Chavez et al 2024 | 10.1177/11786329241280812 | Data from EMR at a single hospital site to investigate antibiotic use at end of life. |
| Mohammad et al 2020 | https://www.sajaa.co.za/index.php/sajaa/article/view/2324 | EMR used to extract details about the management of osteogenesis imperfecta patients at this single hospital, including medicines used around the time of surgery. |
| Moura et al 2011 | 10.1007/BF03256929 | Describes a national system of records of hospitalisations - administrative rather than clinical records including information on drugs prescribed. Unclear if the hospitals use e-prescribing etc, but information is digitalised and managed by government. Database maintained by Brazilian Unified Health System (SUS) |
| Mwita et al 2022 | 10.1177/27550834221098597 | Used data from outpatient electronnic prescription records at a single hospital site to study prevalence of brand-name prescribing. Found higher rates of brand name prescribing in inpatient setting than outpatient and suggests this may be due to inpatient prescribing being paper-based vs electronic in outpatients. |
| Nabovati et al 2016 | 27499793 | Studied potential drug-drug interactions using de-identified database from 2 large insurance companies, of which government has a copy. |
| Namnabati et al 2017 | 10.22038/IJN.2016.20213.1226 | Studied nurse satisfaction with electronic medication administration record and prescription system deployed in neonatal intensive care unit. |
| Naz et al 2021 | 10.1186/s12879-021-06935-6 | Describes electronic national reporting system for tuberculosis (TB) management, which takes the form of a combined excel sheet containing information about the patients’ sociodemographic characteristics, history of TB treatment, regimen, outcomes, comorbidites, current treatment regimen etc. |
| Nguyen et al 2023 | 10.1016/j.lanwpc.2022.100611 | Uses routinely collected e-prescribing data from outpatient settings to study antibiotic prescribing trends for acute respiratory illness in one Vietnamese province. |
| Niazkhani et al 2020 | 10.1186/s12911-020-01196-w | Home-grown system for managing renal transplant patients, includes CPOE and this article describes build and implementation of a clinical decision support system module for drug interactions. |
| Novriani et al 2015 | https://www.atsjournals.org/doi/pdf/10.1164/ajrccm-conference.2015.191.1_MeetingAbstracts.A2776 | Investigated drug resistant tuberculosis treatment quality using EMR data at a tertiary hospital in Indonesia. |
| Nucita et al 2009 | 10.1186/1472-6947-9-42 | Describes multi-national roll-out of Drug Resources Enhancement against AIDS and Malnutrition (DREAM) software for management of PLHIV and antiretroviral therapy (ART). Patient IDs used and may be linked across intranational sites, although this is not clear as seems to require LAN connection. Also has input allowing non-ART medicines to be prescribed with a drop down of formulary items. |
| Nunez-Sanchez et al 2016 | http://www.latamjpharm.org/resumenes/35/8/LAJOP_35_8_1_4.pdf | Prescription errors compared between a hospital with CPOE and one without. Only abstract accessible. |
| Obreli-Neto et al 2011 | 10.1007/s11096-011-9518-x | Investigates medication adherence using computerised prescription records integrated into EMR. |
| Okumura et al 2016 | 10.18549/PharmPract.2016.03.717 | CPOE implemented 9 years prior to study. Investigation of changes in prescribing rates/patterns of a spcecific antibiotic prior to deployment vs after. |
| Oluoch et al 2016 | 10.1016/S2352-3018(15)00242-8 | Regional HIV clinics in Kenya, EMRs rolled-out which contian info on anti-retroviral regimens and track/alert re: immune failure. Kenya Medical Research Institute (KEMRI) assists these clinics with data management. |
| Oreagba et al 2016 | 10.1016/j.jval.2016.03.1983 | Abstract only. Pre-existing e-prescription system used to implement error alerting which decreased prescription errors over 3 year period. |
| Orrell et al 2012 | 10.1097/QAI.0b013e31827e6080 | Intelligent dispensing of antiretroviral therapy (iDART) pharmacy system as well as clinic data manually recorded and computerised on a weekly basis. Compared ART stock consumption with number of patients in clinic who were supposed to be on ART as a measure of treatment adherence. Scanned bottle barcode and linked with patient upon dispensing. |
| Otieno et al 2021 | 10.1111/jch.14174 | Mobile app that stores information about automated blood pressure readings for people with HTN - also allows e-prescription generation remotely. |
| Owusu et al 2018 | 10.1161/hyp.72.suppl_1.P389 | Abstract only. Study to investigate community/pharmacy led hypertension management. E-prescriptions from community physicians already implemented and available to community pharmacies. |
| Oza et al 2017 | 10.2196/jmir.7881 | OpenMRS deployed in one ebola treatment center in Sierra Leone during an outbreak. Functionality included ordering and monitoring medicines, looking up lab results, and documenting patient encounters. |
| Pannoi et al 2024 | 10.7717/peerj.16892 | EMR data from single hospital site used to retrospectively study association between proton pump inhibitors and cardiovasculuar diseases. |
| Peerawaranun et al 2024 | 10.1111/dme.15378 | Describes healthcare structure and data management for public healthcare system in Thailand. Quote: Health service data in Thailand are standardized and stored in electronic health records for both outpatient and inpatient care in all levels of healthcare services, primary to tertiary care, although different programmes and software are used. Personal and medical history, physical examination, diagnoses and treatments, health promotion and disease prevention activities are entered and stored in electronic health records in 43 standard folders, all of which are linked with unique national identification numbers. The data are collated in a government centralized database system called ‘Health Data Center’ and used by the National Health Security Office (NHSO) for reimbursement purposes. All Thai people have to register in one of primary care catchment areas in the province in which they live, and generally receive treatment in the healthcare services in their province. People are able to receive treatments and care outside their registered district and province only if they are referred by their doctors. All hospital admissions nationwide are recorded and identified through the NHSO database. |
| Periasamy et al 2023 | 10.4103/ijstd.ijstd_13_23 | Describes set-up of a sexual health telemedicine and advice service, including provision of instant e-prescriptions. |
| Petrushevska et al 2015 | 10.3889/oamjms.2015.004 | Macedonian Ministry of Health electronic database of prescribed and 'realized' prescriptions. Also used data from health insurance fund. Investigated benzodiazepine prescribing tendencies. Clinic and hospital not yet available within database at point of writing. |
| Pina-Hincapie et al 2020 | 10.1016/j.aller.2019.10.007 | Studied predictors of prescription of albuterol in infants with bronchiolitis using EMR data at one hospital. |
| Pinto Pizzo et al 2015 | 10.3233/978-1-61499-564-7-940 | Brazilian tertiary centre with a new CPOE module, already using EMR. Studied impact on pharmaceutical interventions, noting reduced rates of order transcription. |
| Pongwecharak et al 2007 | 10.1111/j.1365-2753.2006.00680.x | Studied use of aspirin, statins, and renin-angiotensin system inhibitors in diabetic patients. Data from several hospitals collected (small regional 'primary care', up to tertiary teaching hospital). Only one of the hospitals investigated (the secondary/tertiary care one) used an electronic prescribing system. |
| Prasert et al 2018 | 10.1016/j.sapharm.2017.05.012 | Several regional Thai hospitals contributed data, which was used in conjunction with datat from the 'Health Data Center' database where medical and pharmacy claims records are kept. Objective was to study potentially inappropriate medicines. |
| Prasert et al 2019 | 10.1111/jep.13065 | Studied effect of computerised decisiono support system on potentially inappropriate medications using a centralised database of medical and pharmacy claims. There is a nationwide system (Health Data Center), however only four hospitals included in this study. EMR reportedly used 'widely' in public hospitals nationally. |
| Prattanaprateep et al 2017 | 10.1186/s12911-017-0496-3 | Bankok hospital with a database of patients who have attended (in and outpatients). Investigated prescription records to evaluate rational use of nonsteroidal anti-inflammatory drugs and gastroprotection. |
| Priya et al 2017 | 10.4172/pharmaceutical-sciences.1000321 | New e-prescribing system at quarternary centre. CPOE/order entry functionality and prescription records. No mention of dispensing records. |
| Purssell et al 2017 | 10.4269/ajtmh.abstract2016 | Abstract only. Lahore hospital deploying a new CPOE system, after which authors note a decrease in medication errors. |
| Puspitasari et al 2022 | 10.3897/pharmacia.69.e82330 | EMR data from a single community health centre used to study antihypertensive use. |
| Puttkammer et al 2014 | 10.1371/journal.pone.0112261 | Describes development of iSante system for PLHIV data. Prescription and dispensing records plus other information about demographics. |
| Puttkammer et al 2016 | 10.1016/j.ijmedinf.2015.11.003 | Assessment of data quality in a nationwide EMR in Haiti, which has pharmacy records and possibly prescription records. Various methods of data-entry at different sites (point-of-care vs retrospective). |
| Puttkammer et al 2018 | https://www.croiconference.org/abstract/multi-month-scripting-mms-and-retention-hiv-antiretroviral-therapy-haiti/ | Abstract only. Studied antiretroviral prescribing patterns using prescription records in the national iSante database. |
| Puttkammer et al 2020 | 10.1007/s10461-020-02945-8 | Nationally rolled-out EMR system, covering 120 primary and secondary care facilities (as of 2020), most of which provide antiretroviral therapy (ART). Used these data to predict those at risk of missing/failing ART treatment and intervene. |
| Qin et al 2017 | 29295155 | Chinese hospital EMR with CPOE - un/semi-structured data and CPOE data used to detect adverse drug event signals. |
| Qiu et al 2018 | 10.1177/0004867418805559 | Two chinese hospitals with HIS that have been converted to EMRs for research. Investigated antipsychotic polypharmacy. |
| Qureshi et al 2021 | 10.9734/JPRI/2021/v33i44A32609 | Studied prescription errors pre- and post-CPOE deployment at a single hospital site, fining CPOE reduced error rates. |
| Raeesi et al 2021 | 10.1017/S0266462321000052 | E-prescribing system that provides alerts and access to patients' medication history. Used in every Social Security Organization affiliated site in the country. Data not shared between sites. |
| Rafael et al 2019 | 10.22239/2317-269x.01264 | Implemented in 2006, e-prescribing of parenteral nutrition (PN). Private pharmacy specializing in PN. Medical clients use e-prescribing system online which can then be sent to production remotely after algorithm checks general appropriateness of formulation. States most clients of this company are in Sao Paolo Brazil, but it is a multinational company. |
| Raiman et al 2015 | 10.1007/s10875-015-0192-y | Paediatric hospital pharmacy electronic dispensing records for intravenous immunoglobulin used to estimate annual cost. |
| Ratchakit-Nedsuwan et al 2020 | 10.1080/23744235.2019.1688862 | Trial at tertiary centre in northern Thailand using e-pillbox system to monitor adherence to tuberculosis medicines - data automatically update server when box opened/oversue to be opened. |
| Rattanachotphanit et al 2020 | 10.1111/ijpp.12665 | Studies effect of policy on outpatient prescribing safety using ministry of public health (MOPH) hospital databases including historical drug data. Fifteen sites included in study. More than half of MOPH hospitals reportedly still use handwritten prescriptions - despite this in these hospitals the prescriptions are later recorded digitally by the pharmacy department. MOPH hospitals all have prescription data keyed in manually by the pharmacy department, but less than half have e-prescribing, and even fewer have clinical decision support system that extends beyond alerts relating to drug allergies. MOPH health data center receive and maintain data from all affiliated hospitals. |
| Rattanaumpawan et al 2018 | 10.1016/j.jgar.2017.08.015 | Investigated antimicrobial usage for upper respiratory tract infection in outpatients at a tertiary hospital in Bangkok. Hospital pharmacy database used as source of antimicrobials data, and administrative (rather than clinical emrs) ICD-10 coded database for diagnoses . |
| Riaz et al 2014 | 10.1371/journal.pone.0106080 | Lahore based hospital with e-prescribing system. Compared errors with nearby hospital with manual prescribing. |
| Rosa et al 2019 | 10.1590/1806-9282.65.11.1349 | Two tertiary hospitals implemented an e-prescribing feature, measured errors pre and post- introduction, and also with handwritten, pre-typed and 'mixed' prescriptions. |
| Roy et al 2020 | 10.1371/journal.pmed.1003116 | EMR in place in Zambia monitoring anti-retroviral regimens and pick-up dates as part of an 'adherence club' system aimed at improving adherence to anti-retroviral therapy in people with HIV. 'Smartcare' System itself is not HIV specific (used for 'routine clinical care'). |
| Roy et al 2023 | 10.1007/s10792-022-02562-5 | Reports accessing EMR data from system called EyeSmart. Studied microbial keratitis management during COVID-19 pandemic, finding treatment adherence may have been affected. This is a purpose-built system with linkage across a network of healthcare centres. Number of sites mentioned in related article as 176 primary care, 18 secondary care, and 1 tertiary care centre. |
| Ruiz-Talero et al 2020 | 10.1093/intqhc/mzaa037 | Colombian hospital introducing new venous thromboembolism prophylaxis guidelines/interventions. Report EMR apparently containing prescription data for pharamcological venous thromboembolism prophylaxis. |
| Sae-Ang et al 2023 | 10.3390/ijerph20010309 | Uses EMR data from a single hospital site to build machine learning models for recommending medications. |
| Sahu et al 2023 | 10.4103/ijph.ijph_803_22 | Uses sample of prescriptions from 31 primary care facilities within two regions to study compliance with World Health Organization guidance. 1000 e-prescriptions sampled with generally higher rates of compliance among these vs manual prescriptions. Minimal explanation of methods or data sources, unclear which or how many sites use e-prescribing. |
| Saleem et al 2019 | 10.1016/j.heliyon.2019.e02159 | Survey of many centres nationally in Pakistan investigating the nature of antimicrobial stewardship programs. 15% reported having some form of electronic prescribing system and 25% report a clinical decision support system integrated into the health record at time of prescribing. |
| Sãnchez et al 2023 | 10.1177/21501319231196110 | Uses data from EMR of 21 health facilities in one district under management by the Ministry of Public Health. Reports the whole district has used this EMR since 2010. Studies compliance of antibiotic prescriptions with World Health Organization guidelines. |
| Santos et al 2015 | 10.1186/s12887-016-0551-8 | Investigated inappropriate prescribing in neonatal intensive care unit at tertiary hospital using EMR/e-prescribing records. |
| Santos Garcia et al 2020 | 10.1007/s11096-020-01048-7 | Brazilian primary care practice investigating potentially inappropriate medicines from EMR data. Data on the use of medicines were gathered at each appointment - no data about prescriptions/dispensed medicines etc routinely vailable in the EMR. |
| Santoyo-Fexas et al 2020 | 10.1136/annrheumdis-2020-eular.2218 | Abstract only. Rheumatology clinic monitoring medication errors before and after deployment of e-prescribing (system name: REPAIR). No mention of broader EMR, but used retained prescription records to perform analysis and describes user input for system (automated/restricted user input at each stage of prescribing). |
| Serafim et al 2010 | 10.1590/S1807-59322010000400011 | Describing roll-out and scope of informatized drug distribution system (from arrival at pharmacy to administration to patient) at tertiary hospital in Brazil. |
| Seshadri et al 2018 | PMC6247653 | Abstract only (three very similar relevant abstracts within abstract supplement). Efficacy of various anti-diabetic medicines studied using data from CPOE with electronic prescription and dispensing records at a private company running diabetes specialist clinics across India with over 75000 patients. |
| Shang et al 2024 | 10.3389/fphar.2024.1302154 | Uses EMR prescription data from a single hospital site to study the impact of national procurement strategy on use of antihypertensive medicines. |
| Shawahna et al 2011 | 10.1111/j.1365-2702.2011.03714.x | Hospital introduced EMR and e-prescribing system. Used prescription records to compare pre and post deployment medication errors. |
| Shen et al 2020 | 10.1080/13696998.2020.1717500 | Multicentre oncology datbase including data on chemotherapy regimens, includes information on chemotherapy regimens from multiple tertiary centres, with 12 sites included for this study. Participating hospitals all have EMR, and shared oncology data is inferred to be managed by a network/organisation. |
| Siddiqui et al 2007 | 17990421 | Lahore based tertiary centre uses computer based antibiotic prescribing system which is linked to the pharmacy. Pharmacist would alert physicians after 48h of an order for broad spectrum antibiotics that it was due to expire, intended to discourage excessive use of these medicines to reduce risk of resistant bacteria developing. |
| Silva et al 2008 | https://www.researchgate.net/publication/289852244_The_process_of_drug_dispensing_and_distribution_at_four_Brazilian_hospitals_A_multicenter_descriptive_study | Survey of 4 Brazilian hospitals' prescribing/dispensing systems. One reported use of CPOE and barcode dispensing. None of the others used an electronic system for these functions. |
| Singh et al 2023 | 10.1080/17434440.2023.2264768 | e-Sanjeevai system in use at a tertiary hospital outpatient department. Reports use of e-prescriptions with little other information about EMMS in the abstract. Unable to access full article. |
| Skosana et al 2021 | 10.1016/j.jgar.2021.12.003 | Purpose-built web app for antimicrobial stewardship through point-prevalence monitoring. Described in more detail in Kruger et al 2021. |
| Souza e Silva et al 2015 | 10.15448/1980-6108.2015.4.21373 | EMR used to investigate patient profile and characteristics of those receiving antipsychotics. |
| Su et al 2021 | 10.1001/jamanetworkopen.2021.0775 | Data from many Chinese tertiary centres, all seem to have a local EMRs with data being extracted from all participating hospitals and then pooled, suggesting no direct linkage of systems/routine sharing of data. Data source: Epidemiology of AKI in Chinese Hospitalized Patients (EACH). |
| Surapat et al 2021 | 10.1111/jcpt.13293 | CPOE system in tertiary Thai hospital used to identify patients needing remote pharmacist intervention for drug monitoring etc during COVID pandemic. |
| Syarkevych et al 2016 | 10.1016/j.jval.2016.09.1935 | Abstract only. Describes insulin use/reimbursement records on national database specifically for diabetes patients. |
| Syowai et al 2020 | 10.1371/journal.pone.0232104 | Nationally used EMR and pharmacy 'antiretroviral dispensing tool' data for PLHIV. Each system had different patient IDs but data were linked at an individual level for this paper. |
| Tan et al 2022 | 10.3389/fped.2022.880928 | Analysed electronic prescriptions from a sample of primary health facilities in a region. Found inappropraite prescriptions most common with acute upper respiratory infections. Minimal description of data source or EMMS. |
| Tan et al 2024 | 10.1007/s10654-024-01124-6 | Region-wide health data systems described, with paper focusing on development of a new large cohort for monitoring maternal health and congenital abnormalities using routinely collected health data. |
| Tanaka et al 2015 | https://www.redalyc.org/pdf/469/46935880003.pdf | Cost analysis of e-prescription for paediatric dietary supplements/feeds in a hospital with e-prescribing, prescription records, and barcode dispensing |
| Tang et al 2019 | 10.2196/12577 | Investigated medications given in paediatric pneumonia - describes machine learning algorithms used to mine for patterns of prescribing in medicine administration record as part of EMR. |
| Tansitpong et al 2020 | 10.18178/ijpmbs.9.2.96-101 | Studied medicines use and variation in prescribing practice in national records of treatment costs and and diagnoses. A single hospital's local EMR database was investigated for this article, but exact geographical location is not specified. |
| Thompson et al 2010 | 10.3233/978-1-60750-588-4-332 | Describes pilot roll-out of OpenMRS in one clinic in Northern Nigeria. Directed at improving maternal and child health. EMR containing records on immunizations. |
| Tian et al 2022 | 10.3389/fphar.2022.935764 | Used electronic medical data from more than one hospital site outpatient department to study polypharmacy and inappropriate medicine use in older lung cancer patients. |
| Tierney et al 2010 | 10.3233/978-1-60750-588-4-371 | OpenMRS deployed in three countries, sites: 23 (kenya), 3 (tanz), 3 (uganda). Describes function and mentions records of 'treatments' as well as lots of other patient info (demographics etc) for PLHIV. Unclear if prescription records, dispensing records or other. While mostly used for PLHIV, uses have been diversified especially in Kenya. |
| Tilahun et al 2015 | 10.2196/medinform.4106 | Describes SmartCare system in use in Zambia, Ethiopia and South Africa, however this is a modular platform and unclear which modules are in use at chich sites/in which countries. Ethiopian deployments described in more detail - more than 5 sites had started using the system, and it had been adopted as the National EMR in 2013. Pharmacy and drug stock control system, as well as anti-retroviral management, plus EMR in- and out-patient functionality. |
| Tong et al 2023 | 10.3389/fphar.2023.1216182 | Used EMR data from a single hospital site to build machine learning models to predict HbA1c levels. |
| Tragulpiankit et al 2014 | https://onlinelibrary.wiley.com/doi/epdf/10.1002/pds.3701?saml_referrer | Abstract only. EMR at Thai teaching hospital used to investigate drug-related re-admissions. Most commonly suspected drug related readmissions were due to hypoglycaemia in older individuals with diabetes. |
| Truter 2001 | 10.1002/pds.647 | Evaluates ethical issues around selected retrospective medication use studies. Describes methods relevant to similar articles by same lead author. Database of nationwide private sector community pharmacy electronic dispensing records. ~10% of local pharmacies included in database. Data from pharmacies in all 9 provinces in SA. Unique patient ID mentioned. No description of system used at point-of-care |
| Truter 2016 | 10.1080/16070658.2016.1217643 | Studies dispensing patterns of prescription weight-loss medicines. Data source as for other publications by same lead author, see Truter 2001. |
| Truter 2017 | 10.1016/j.jval.2017.08.840 | Abstract only. Studies dispensing patterns of prescription weight-loss medicines. Data source as for other publications by same lead author, see Truter 2001. |
| Truter 2018 | 10.1016/j.jval.2018.09.2603 | Abstract only. Studies dispensing patterns of prescription opioids. Data source as for other publications by same lead author, see Truter 2001. |
| Truter et al 2004 | 10.4102/hsag.v9i4.180 | Studies dispensing patterns of prescription migraine medicines. Data source as for other publications by same lead author, see Truter 2001. |
| Truter et al 2016 | 10.1080/16070658.2016.1219468 | Abstract only. Studies dispensing patterns of vitamin products. Data source as for other publications by same lead author, see Truter 2001. |
| Vaidotas et al 2019 | 10.31744/einstein_journal/2019GS4282 | One hospital with 4 sites implemented new EMR at different times and measured error number and type before and after. Involved in prescribing and reimbursement, with data from dispensary invoices. |
| Valencia et al 2018 | 10.1200/CCI.18.00041 | Describes roll-out of a CPOE system, but only discusses chemotherapy e-prescribing component. Mentions local EMR system of which this new subsystem is now a part. Mentions similar systems are in place in Argentina, Brazil, Mexico, Panama, and Uruguay, but mostly in the private sector - insufficient additional detail/references provided regarding these locations. |
| Van Der Hoven et al 2022 | 10.1186/s12884-022-04765-1 | Describes Western Cape Pregnancy Exposure Registry with digital records including medicine use. Also uses data from the Western Cape Provincial Health Data Centre which contains dispensing data from electronic pharmacy systems. Compares different methods for ascertaining medicine exposure during pregnancy. |
| Varavithya et al 2022 | 10.1007/s00228-021-03269-9 | Retrospective EMR data study of potentially inappropriate medicine use among elderly patients at one outpatient hospital site, and their association with hospitalization. |
| Vatcharavongvan et al 2017 | 10.5114/fmpcr.2017.70818 | Primay care practice patients with polypharmacy studied using EMR data. |
| Vatcharavongvan et al 2019 | 10.18549/PharmPract.2019.3.1494 | Thai primary care clinic with EMR containing data on prescribed drugs, used to investigate potentially inappropriate medicines/polypharmacy etc. Records include national identification numbers. |
| Vatcharavongvan et al 2021 | 10.5770/cgj.24.516 | Data from a sample of primary care units' Health Data Center (HDC) databases used to retrospectively study potentially inappropriate medicines usage among elderly patients. |
| Volpe et al 2016 | 10.1590/1518-8345.0642.2742 | Studied medication errors with electronic and manual prescriptions within a system at a single hospital, implemented in 2011, incorporating EMR and e-prescribing. |
| Vonasek et al 2021 | 10.1097/QAD.0000000000002715 | Investigated tuberculosis incidence and antituberculosis treatment in multi-site EMR data among children and adolescents living with HIV utilising HIV services/clinics. |
| Wang et al 2016 | 10.1016/j.ijantimicag.2016.09.008 | Studied antimicrobial prescribing patterns within a local EMR at a single, large hospital in Shanghai. Both outpatient and inpatient functions - many patients use walk-ins as means to primary care needs. |
| Wang et al 2017 | http://www.pjps.pk/wp-content/uploads/pdfs/30/3/Paper-7.pdf | CPOE rolled out, e-prescribing is only module described, unclear if other parts. Medicines are scanned when leaving pharmacy, and also prior to administration. Primarily investigated as a means to reduce chemotherapy prescriptipon errors. |
| Wang et al 2022 | 10.1016/j.jad.2022.02.054 | Uses EMR data from two geographically distant hospital sites to study incidence and treatment of treatment resistant depression. |
| Wang et al 2022 | 10.1186/s12875-022-01875-9 | Uses EMR data from 75 primary healthcare centres in one region of China to study antibiotic prescribing patterns. |
| Wang et al 2023 | 10.3390/antibiotics12020297 | Uses EMR data from 37 primary healthcare centres in one region of China to study antibiotic prescribing patterns. |
| Wang, Dai et al 2020 | 10.1111/jcpt.13152 | Survey responses from pharmacists across country re: anti-infective treatment protocols and systems in place. Varying numbers reported HIS that helped them identify patients on certain antibiotics for e.g. de-prescribing. 103 sites had automatic notification of pharmacists for antimocrobial use consults. Individual systems not described in detail. |
| Wang, Liu et al 2020 | 10.3390/antibiotics9120876 | Studied antibiotic prescribing behaviour using consultation records and electronic prescription records from primary care records collected in Hubei region. |
| Wang, Peng et al 2019 | 10.1038/s41430-018-0281-0 | Tpotal parenteral nutrition e-prescription introduced at tertiaty hospital to optimise prescriptions. |
| Wang, Wang et al 2019 | 10.36468/pharmaceutical-sciences.544 | Regionally linked database of osteoarthritis patients - prescription and other records centralised. Investigated which drugs were prescribed. Unclear if e-prescribing is a feature. |
| Wang, Xuan et al 2020 | 10.1016/j.socscimed.2020.113035 | Interviewed community pharmacists to investigate non-prescription antibiotic prescribing. Superficially describes the use of e-prescriptions and dispensing records in various settings and organisational levels. No specific descriptions of a system at any particular location. |
| Weeks et al 2017 | 10.1016/j.aogh.2017.03.333 | Abstract only. Non-governmental organization mission outreach in a community in the Dominican Republic. Used EMR and measured appropriate prescribing through prescription records prior to and after deployment of an EMR system. |
| Wei et al 2019 | 10.1371/journal.pmed.1002733 | Hospital records for primary care outpatient clinics. Investigating antibiotics prescribing in child upper respiratory tract infection - only one county had electronic records of prescriptions. |
| Wei et al 2022 | 10.1080/17538157.2021.1990932 | Studies subjective mental workload of doctors using a CPOE system at a single hospital site. |
| Wei et al 2023 | 10.1177/03000605231204465 | Uses EMR data from 3 hospitals in one region to retrospectively study bladder cancer management. Does not report whether medicines data is for prescribing, dispensing, or other data sources. |
| Wei et al 2024 | 10.1016/S0140-6736(23)02270-5 | Reports a randomized controlled trial to establish if an electronic medication monitor improves adherence and treatment success for tuberculosis. Reports that following study's positive results the system is now used throughout Tibet. |
| Winijkul et al 2021 | 10.1186/s12872-021-01928-4 | Electronic registry of patients with atrial fibrillation in Thailand - includes information on antithrombotic therapy. |
| Wu et al 2016 | 10.1016/j.ijmedinf.2016.01.002 | Full CPOE, e-prescribing described within. Resident doctors were sampled from a Beijing hospital and tested on a system that mimicked their usual CPOE system to assess sources of transcribing/prescribing errors. |
| Wu et al 2021 | 10.1155/2021/9468227 | Identified patients with extrahepatic cholangiocarcinoma using EMR data to assist with building a cohort for studyingthe effect of metformin on survival. Minimal information about EMMS. |
| Xi et al 2023 | 10.36721/PJPS.2023.36.3.REG.879-885.1 | Studied irrational electronic prescribing in a single hospital outpatient department. Minimal information about EMMS, able to access abstract only. |
| Xia et al 2014 | 10.1093/trstmh/tru066 | Studies prevalence of extended treatment for pulmonary tuberculosis and its association with recurrence of disease. Tuberculosis database, containing information on patient demographics, directly observed therapy data, and drug usage. Study site is in Beijing, but article describes both their local medical records system and a national tuberculosis information system (NTIS) which contains information on a national level for all registered TB patients in China. |
| Xiao et al 2022 | 10.1097/CIN.0000000000000809 | Ethnographic study of physician-nurse communication in relation to a CPOE system implemented in 2005 at a tertiary teaching hospital. |
| Xin et al 2015 | 10.2147/PPA.S84411 | Investigates outcomes of pharmacist led educational intervention to improve insulin adherence. Minimal description of EMMS but reports relevance to both prescribing and dispensing. |
| Xu et al 2016 | 10.1016/j.ijmedinf.2016.02.008 | Full EMR at two tertiary centres, included CPOE, e-prescribing, list of regular medicines/allergies, and full medical record keeping. In one of the figures there is input for an MRN and this is mentioned briefly in text as well. |
| Xu et al 2021 | 10.1111/jcpt.13397 | Used EMR and CPOE data from a single hospital site to study effects of pharmacist interventions to improve intravenous to oral antibiotic switching. |
| Xu et al 2022 | 10.1192/bjo.2022.609 | Uses electronic prescription records data from 5 hospitals in geographically distinct areas to study benzodiazepine receptor agonist use in psychiatric settings. |
| Yan et al 2017 | 10.1186/s12913-017-2063-0 | Mixed methods study conducted at Zambian primary care hypertension clinics with full EMR with e-prescriptions. |
| Yan et al 2018 | 10.1159/000488591 | Local 'internet hospital' set up and used in this case to investigate outcomes for patients discharged with chronic obstructive pulmonary disease. Describes system at this virtual hospital which has EMR, e-prescribing, online ordering, and drug delivery (to a location, not administration of drug). . |
| Yang et al 2013 | https://pesquisa.bvsalud.org/portal/resource/pt/wpr-860142 | Abstract only. Investigated e-prescriptions for antibiotics at 79 hospitals around China before and after changes in national antibiotics guidance (around 2011). Data source: Hospital prescription analysis program |
| Yang, Liao et al 2019 | 10.1186/s12913-019-4843-1 | Describes locally deployed (in 2004) e-prescribing system, investigated rates of prescription errors. |
| Yang, Lin et al 2019 | 10.1007/s11096-019-00788-5 | Investigation of off-label tamoxifen use in a tertiary hospital in China. Describes e-prescribing system and numerous patient demorgaphics as part of the broader hospital information system. |
| Yue et al 2020 | 10.1177/0300060520914197 | Exploring medicine usage at neonatal unit in tertiary hospital. Data on medicines extracted from EMR and electronic database of prescriptions. |
| Yugandhar et al 2017 | 10.5530/jyp.2017.9.86 | E-prescribing system rolled-out for discharge prescribing only at this hospital. Prescription errors compared with and without e-prescribing. |
| Yulistiani et al 2023 | 10.3897/pharmacia.70.e101609 | E-prescription data from geriatrics outpatient clinic at a single hospital site used to study use of medicines that increase fall risk among older patients. |
| Yusof et al 2023 | 10.1093/ijpp/riac104 | Electronic database of prescriptions at a tertiary hospital used to study proton pump inhibitor prescribing treands over time. |
| Zaffar et al 2020 | 10.5455/JPMA.18218 | Investigated attitudes and knowledge of e-prescribing at a hospital where e-prescribing was already used. Does not describe the system itself in any further detail. |
| Zaliska et al 2020 | 10.1016/j.jval.2020.08.725 | Ukrainian national database of electronic prescriptions. Investigating uptake of new reimbursement programme. E-prescriptions utilised nationally since 2019. |
| Zaliska et al 2021 | https://www.valueinhealthjournal.com/article/S1098-3015(21)01106-2/pdf | Uses national database of e-prescriptions to study trends in e-prescription use under a specific government program set up in 2019. Found increasing uptake in all regions. Abstract only. |
| Zaliska et al 2021 | 10.1016/j.jval.2021.04.380 | Uses national database of e-prescriptions to study trends in medicine use under a specific government program for provision of cardiovascular medicines which was set up in 2017, with e-prescriptions introduced as part of program in 2019. Study found increasing costs of reimbusement and that over 50% of prescriptions were for only four medicines. Abstract only. |
| Zare et al 2024 | 10.32598/RJ.25.1.3211.3 | Data extracted from hospital information system, including data about medicine prescriptions, to study reasons for insurance deductions. Found deductions mostly related to drug and consumable expenses and surgical fees, and were caused by incorrect coding or excessive requests. |
| Zaver et al 2020 | https://accp.confex.com/accp/2020am/meetingapp.cgi/Paper/55674 | Abstract only. Studied how an electronic dispensary and inventory management system impacted stock outs at a rural clinic in Honduras. Same system as Idrees et al 2018. |
| Zhang et al 2016 | 10.1186/s40064-016-3701-4 | Large tertiary centre in China, describes implementing a rule-based medication error tool to EMR and CPOE systems. |
| Zhang et al 2017 | 10.1080/16549716.2017.1287334 | Regional hospitals with electronic medical records and prescription records. County level hospitals have electronic records and township hospitals have paper records. Investigated antibiotics prescribing trends. |
| Zhang et al 2020 | 10.1007/s12325-020-01271-8 | Two centres from different provinces with unlinked EMRs - investigated cardiac events in patients prescribed drugs for gastrointestinal conditions. |
| Zhang et al 2021 | 10.1186/s12879-021-05804-6 | Conducted a point-prevalence survey of antibiotic prescriptions at a single hospital with an e-prescribing system. |
| Zhang et al 2023 | 10.1093/eurpub/ckad138 | Internet hospital set up as a section of an existing hospital to meet COVID-19 pandemic needs, included e-prescription service. Briefly describes set up process, and investigates demographics, symptoms, and medicines used. |
| Zhang et al 2024 | 10.1186/s12912-024-02178-3 | Clinical trial describing the roll-out of nursing EMR quality control system at a single tertiary hospital. Describes barcode administration system of e-prescribed medicines. Found computerized documentation quality control system reduced time spent on this procedure. |
| Zhao et al 2022 | 10.3389/fphar.2022.946415 | Used EMR data from one hospital site to study potential drug-drug interactions among patients treated for 'chronic coronary syndrome'. |
| Zhao et al 2024 | 10.1089/tmj.2023.0025 | Survey study to investigate trends in use of internet medical services in China. Responses from ~4800 hospitals, about 20% of which had e-prescribing, though this was >3 times as prevalent in tertiary settings. Unable to access full article. |
| Zhou et al 2008 | 10.3748/wjg.14.6065 | Details of clarithromycin use for gastrointestinal diseases extracted from e-prescriptions at a single hospital over 1-year period. |
| Zhou et al 2023 | 10.1186/s12913-023-09742-8 | Describes gradual region-wide adoption of e-prescribing functionality at community pharmacies, provided by 9 different third-parties. Study in question used trained participants to simulate patients with different care needs and requests. Found frequent non-prescription dispensing based on patient request rather than appropriateness. |
| Zhu et al 2017 | 10.1097/MD.0000000000006475 | Studied patient characteristics and perceived health status of individuals with HIV and tuberculosis at a single hospital. Very little description of system beyond mention of extraction of medication information from electronic records. |
| Zhu et al 2024 | 10.2147/CIA.S450319 | Reports narratively a series of adverse events related to medicines reconciliation at point of discharge from one hospital, and makes specific mention of the presence of a CPOE system. |

**Supplementary table S3: A list of countries eligible for inclusion**

N.B. The 2020 UN Human Development report does not report data for Somalia (hence it is not included in the table below), however, we considered it eligible for inclusion based on other sources suggesting it has a sufficiently low HDI. [https://globaldatalab.org/shdi/table/shdi/SOM/?levels=1]

| **Country** | **HDI group** |
| --- | --- |
| Afghanistan | Low |
| Albania | High |
| Algeria | High |
| Angola | Medium |
| Antigua | High |
| Barbuda | High |
| Armenia | High |
| Azerbaijan | High |
| Bangladesh | Medium |
| Belize | High |
| Benin | Low |
| Bhutan | Medium |
| Bolivia | High |
| Bosnia and Herzegovina | High |
| Botswana | High |
| Brazil | High |
| Burkina Faso | Low |
| Burundi | Low |
| Cape Verde | Medium |
| Cambodia | Medium |
| Cameroon | Medium |
| Central African Republic | Low |
| Chad | Low |
| China | High |
| Colombia | High |
| Comoros | Medium |
| Republic of Congo | Medium |
| Democratic Republic of the Congo | Low |
| Ivory Coast | Low |
| Cuba | High |
| Djibouti | Low |
| Dominica | High |
| Dominican Republic | High |
| Ecuador | High |
| Egypt | High |
| El Salvador | Medium |
| Equatorial Guinea | Medium |
| Eritrea | Low |
| Swaziland | Medium |
| Ethiopia | Low |
| Fiji | High |
| Gabon | High |
| Gambia | Low |
| Ghana | Medium |
| Grenada | High |
| Guatemala | Medium |
| Guinea | Low |
| Guinea-Bissau | Low |
| Guyana | Medium |
| Haiti | Low |
| Honduras | Medium |
| India | Medium |
| Indonesia | High |
| Iran | High |
| Iraq | Medium |
| Jamaica | High |
| Jordan | High |
| Kenya | Medium |
| Kiribati | Medium |
| Kyrgyzstan | Medium |
| Laos | Medium |
| Lebanon | High |
| Lesotho | Low |
| Liberia | Low |
| Libya | High |
| Madagascar | Low |
| Malawi | Low |
| Maldives | High |
| Mali | Low |
| Marshall Islands | High |
| Mauritania | Low |
| Mexico | High |
| Micronesia | Medium |
| Moldova | High |
| Mongolia | High |
| Morocco | Medium |
| Mozambique | Low |
| Myanmar | Medium |
| Namibia | Medium |
| Nepal | Medium |
| Nicaragua | Medium |
| Niger | Low |
| Nigeria | Low |
| North Macedonia | High |
| Pakistan | Medium |
| Palestine | High |
| Papua New Guinea | Medium |
| Paraguay | High |
| Peru | High |
| Philippines | High |
| Rwanda | Low |
| Nevis | High |
| Saint Kitts | High |
| Saint Lucia | High |
| Saint Vincent | High |
| Grenadines | High |
| Samoa | High |
| Sao Tome and Principe | Medium |
| Senegal | Low |
| Seychelles | High |
| Sierra Leone | Low |
| Solomon Islands | Medium |
| South Africa | High |
| South Sudan | Low |
| Sri Lanka | High |
| Sudan | Low |
| Suriname | High |
| Syria | Medium |
| Tajikistan | Medium |
| Tanzania | Low |
| Thailand | High |
| Timor-Leste | Medium |
| Togo | Low |
| Tonga | High |
| Trinidad | High |
| Tobago | High |
| Tunisia | High |
| Turkmenistan | High |
| Uganda | Low |
| Ukraine | High |
| Uzbekistan | High |
| Vanuatu | Medium |
| Venezuela | High |
| Vietnam | High |
| Yemen | Low |
| Zambia | Medium |
| Zimbabwe | Medium |

**Section 2: Summary of reasons for exclusion of articles at the full-text stage**

Irrelevant (excluded at initial screening): 2023

816 total excluded at full text stage with reasons:

Developed country: 366

Not medicines management system: 205

No clinical/full deployment of system (e.g. prototype, or only used in the context of the respective study): 147

No EMMS described: 5

Review/commentary/editorial/conference proceedings: 37

Manually identified duplicates: 7

Location not reported: 1

No patient/prescription-level data: 19

Not electronic: 19

Protocol/registration only: 17

Unable to access adequate full text: 12

**Section 3: Search strategy**

Detailed search terms provided below. All searches updated on 23/10/2024

MEDLINE

Ovid MEDLINE(R) ALL <1946 to May 24, 2021>

1 ((electronic or computeri$ed or digital) adj5 prescri*).ti,kw,ab. **1812**

2 "e-prescri*".ti,kw,ab. **526**

3 (barcode adj10 (drug* or medic*)).ti,kw,ab. **246**

4 (barcode adj10 dispens*).ti,kw,ab. **22**

5 ((digital or electronic or computeri$ed) adj5 dispens*).ti,kw,ab. **214**

6 ((digital or electronic or computeri$ed) and ((drug* or medic*) adj3 administration record*)).ti,kw,ab. **129**

7 ((digital or electronic or computeri$ed) adj5 medication record*).ti,kw,ab. **37**

8 ((digital or electronic or computeri$ed) and ((drug* or medic*) adj2 management)).ti,kw,ab. **1184**

9 "computerised physician order entry".ti,kw,ab. **89**

10 "computerized physician order entry".ti,kw,ab. **803**

11 "computerised provider order entry".ti,kw,ab. **40**

12 "computerized provider order entry".ti,kw,ab. **453**

13 CPOE.ti,kw,ab. **1123**

14 exp drug therapy, computer-assisted/ **1683**

15 exp Medical Order Entry Systems/ **2324**

16 Electronic Prescribing/cl, ec, nu, st, sn [Classification, Economics, Nursing, Standards, Statistics & Numerical Data] **437**

17 1 or 2 or 3 or 4 or 5 or 6 or 7 or 8 or 9 or 10 or 11 or 12 or 13 **5353**

18 14 or 15 or 16 **4154**

19 17 or 18 **8029**

20 (electronic health record* adj10 (medicine* or drug* or prescri* or dispens*)).ti,ab. **899**

21 (EHR adj10 (medicine* or drug* or prescri* or dispens*)).ti,ab. **446**

22 (computerised health record* adj10 (medicine* or drug* or prescri* or dispens*)).ti,ab. **3**

23 (computerized health record* adj10 (medicine* or drug* or prescri* or dispens*)).ti,ab. **2**

24 (electronic medical record* adj10 (medicine* or drug* or prescri* or dispens*)).ti,ab. **785**

25 (EMR adj10 (medicine* or drug* or prescri* or dispens*)).ti,ab. **269**

26 20 or 21 or 22 or 23 or 24 or 25 **2091**

27 19 or 26 **9602**

28 Developing Countries.sh,kf. **88292**

29 Africa/ or Asia/ or Caribbean/ or West Indies/ or Middle East/ or South America/ or Latin America/ or Central America/ **85993**

30 (Africa or Asia or Caribbean or West Indies or Middle East or South America or Latin America or Central America).tw. **219095**

31 ((Africa or Asia or Caribbean or "West Indies" or "Middle East" or "South America" or "Latin America" or "Central America" or Afghanistan or Albania or Algeria or Angola or Argentina or Armenia or Armenian or Azerbaijan or Bangladesh or Benin or Byelarus or Byelorussian or Belarus or Belorussian or Belorussia or Belize or Bhutan or Bolivia or Bosnia or Herzegovina or Hercegovina or Botswana or Brazil or Bulgaria or "Burkina Faso" or "Burkina Fasso" or "Upper Volta" or Burundi or Urundi or Cambodia or "Khmer Republic" or Kampuchea or Cameroon or Cameroons or Cameron or Camerons or "Cape Verde" or "Central African Republic" or Chad or China or Colombia or Comoros or "Comoro Islands" or Comores or Mayotte or Congo or Zaire or "Costa Rica" or "Cote d'Ivoire" or "Ivory Coast" or Cuba or Djibouti or "French Somaliland" or Dominica or "Dominican Republic" or "East Timor" or "East Timur" or "Timor Leste" or Ecuador or Egypt or "United Arab Republic" or "El Salvador" or Eritrea or Ethiopia or Fiji or Gabon or Gabonese Republic or Gambia or Gaza or Georgia Republic or Georgian Republic or Ghana or Grenada or Guatemala or Guinea or Guiana or Guyana or Haiti or Honduras or India or Maldives or Indonesia or Iran or Iraq or Jamaica or Jordan or Kazakhstan or Kazakh or Kenya or Kiribati or Korea or Kosovo or Kyrgyzstan or Kirghizia or "Kyrgyz Republic" or Kirghiz or Kirgizstan or "Lao PDR" or Laos or Lebanon or Lesotho or Basutoland or Liberia or Libya or Macedonia or Madagascar or "Malagasy Republic" or Malaysia or Malaya or Malay or Sabah or Sarawak or Malawi or Mali or "Marshall Islands" or Mauritania or Mauritius or "Agalega Islands" or Mexico or Micronesia or "Middle East" or Moldova or Moldovia or Moldovian or Mongolia or Montenegro or Morocco or Ifni or Mozambique or Myanmar or Myanma or Burma or Namibia or Nepal or "Netherlands Antilles" or Nicaragua or Niger or Nigeria or Muscat or Pakistan or Palau or Palestine or Panama or Paraguay or Peru or Philippines or Philipines or Phillipines or Phillippines or "Papua New Guinea" or Romania or Rumania or Roumania or Rwanda or Ruanda or "Saint Lucia" or "St Lucia" or "Saint Vincent" or "St Vincent" or Grenadines or Samoa or "Samoan Islands" or "Navigator Island" or "Navigator Islands" or "Sao Tome" or Senegal or Serbia or Montenegro or Seychelles or "Sierra Leone" or "Sri Lanka" or "Solomon Islands" or Somalia or Sudan or Suriname or Surinam or Swaziland or "South Africa" or Syria or Tajikistan or Tadzhikistan or Tadjikistan or Tadzhik or Tanzania or Thailand or Togo or "Togolese Republic" or Tonga or Tunisia or Turkey or Turkmenistan or Turkmen or Uganda or Ukraine or Uzbekistan or Uzbek or Vanuatu or "New Hebrides" or Venezuela or Vietnam or "Viet Nam" or "West Bank" or Yemen or Zambia or Zimbabwe) not ("African-American*" or "African American*" or "Mexican American*" or "American Indian*" or "Asian American*" or "native american*" or "south korea*")).ti,ab. **1258174**

32 exp africa/ or algeria/ or egypt/ or libya/ or morocco/ or tunisia/ or cameroon/ or central african republic/ or chad/ or congo/ or "democratic republic of the congo"/ or equatorial guinea/ or gabon/ or burundi/ or djibouti/ or eritrea/ or ethiopia/ or kenya/ or rwanda/ or somalia/ or south sudan/ or sudan/ or tanzania/ or uganda/ or angola/ or botswana/ or lesotho/ or malawi/ or mozambique/ or namibia/ or south africa/ or swaziland/ or zambia/ or zimbabwe/ or benin/ or burkina faso/ or cape verde/ or cote d'ivoire/ or gambia/ or ghana/ or guinea/ or guinea-bissau/ or liberia/ or mali/ or mauritania/ or niger/ or nigeria/ or senegal/ or sierra leone/ or togo/ or americas/ or exp caribbean region/ or exp west indies/ or exp central america/ or belize/ or costa rica/ or el salvador/ or guatemala/ or honduras/ or nicaragua/ or panama/ or panama canal zone/ or latin america/ or mexico/ or exp south america/ or argentina/ or bolivia/ or brazil/ or chile/ or colombia/ or ecuador/ or french guiana/ or guyana/ or paraguay/ or peru/ or suriname/ or uruguay/ or venezuela/ or asia/ or asia, central/ or kazakhstan/ or kyrgyzstan/ or tajikistan/ or turkmenistan/ or uzbekistan/ or exp asia, southeastern/ or borneo/ or brunei/ or cambodia/ or timor-leste/ or indonesia/ or laos/ or malaysia/ or mekong valley/ or myanmar/ or philippines/ or singapore/ or thailand/ or vietnam/ or asia, western/ or bangladesh/ or bhutan/ or india/ or sikkim/ or middle east/ or afghanistan/ or bahrain/ or iran/ or iraq/ or israel/ or jordan/ or kuwait/ or lebanon/ or oman/ or qatar/ or saudi arabia/ or syria/ or turkey/ or united arab emirates/ or yemen/ or nepal/ or pakistan/ or sri lanka/ or far east/ or china/ or beijing/ or macau/ or tibet/ or korea/ or mongolia/ or taiwan/ or indian ocean islands/ or comoros/ or madagascar/ or mauritius/ or reunion/ or seychelles/ or pacific islands/ or exp melanesia/ or exp micronesia/ or polynesia/ or pitcairn island/ or exp samoa/ or tonga/ or prince edward island/ or west indies/ or "antigua and barbuda"/ or bahamas/ or barbados/ or cuba/ or dominica/ or dominican republic/ or grenada/ or guadeloupe/ or haiti/ or jamaica/ or martinique/ or netherlands antilles/ or puerto rico/ or "saint kitts and nevis"/ or saint lucia/ or "saint vincent and the grenadines"/ or "trinidad and tobago"/ or united states virgin islands/ or oceania/ **1206454**

33 ((developing or less* developed or under developed or underdeveloped or middle income or low* income or underserved or under served or deprived or poor*) adj (countr* or nation? or population? or world or state*)).ti,ab. **110939**

34 ((developing or less* developed or under developed or underdeveloped or middle income or low* income) adj (economy or economies)).ti,ab. **665**

35 (low* adj (gdp or gnp or gross domestic or gross national)).tw. **281**

36 (low adj3 middle adj3 countr*).tw. **20394**

37 (lmic or lmics or third world or lami countr*).tw. **9053**

38 transitional countr*.tw. **170**

39 28 or 29 or 30 or 31 or 32 or 33 or 34 or 35 or 36 or 37 or 38 **1846855**

40 27 and 39 **411**

Embase

Embase <1974 to 2021 May 24>

1 ((electronic or computeri$ed or digital) adj5 prescri*).ti,kw,ab. **4041**

2 "e-prescri*".ti,kw,ab. **871**

3 (barcode adj10 (drug* or medic*)).ti,kw,ab. **366**

4 (barcode adj10 dispens*).ti,kw,ab. **40**

5 ((digital or electronic or computeri$ed) adj5 dispens*).ti,kw,ab. **418**

6 ((digital or electronic or computeri$ed) and ((drug* or medic*) adj3 administration record*)).ti,kw,ab. **244**

7 ((digital or electronic or computeri$ed) adj5 medication record*).ti,kw,ab. **102**

8 ((digital or electronic or computeri$ed) and ((drug* or medic*) adj2 management)).ti,kw,ab. **2337**

9 (electronic health record* adj10 (medicine* or drug* or prescri* or dispens*)).ti,ab. **1425**

10 (EHR adj10 (medicine* or drug* or prescri* or dispens*)).ti,ab. **873**

11 (computerised health record* adj10 (medicine* or drug* or prescri* or dispens*)).ti,ab. **4**

12 (computerized health record* adj10 (medicine* or drug* or prescri* or dispens*)).ti,ab. **3**

13 (electronic medical record* adj10 (medicine* or drug* or prescri* or dispens*)).ti,ab. **1735**

14 (EMR adj10 (medicine* or drug* or prescri* or dispens*)).ti,ab. **735**

15 "computerised physician order entry".ti,kw,ab. **185**

16 "computerized physician order entry".ti,kw,ab. **1250**

17 "computerised provider order entry".ti,kw,ab. **48**

18 "computerized provider order entry".ti,kw,ab. **559**

19 CPOE.ti,kw,ab. **1731**

20 exp drug therapy, computer-assisted/ **927**

21 1 or 2 or 3 or 4 or 5 or 6 or 7 or 8 or 9 or 10 or 11 or 12 or 13 or 14 or 15 or 16 or 17 or 18 or 19 **13261**

22 exp computerized provider order entry/ **5299**

23 exp physician order entry system/ **287**

24 Africa/ or Asia/ or Caribbean/ or West Indies/ or Middle East/ or South America/ or Latin America/ or Central America/ **163443**

25 (Africa or Asia or Caribbean or West Indies or Middle East or South America or Latin America or Central America).tw. **273221**

26 ((Africa or Asia or Caribbean or "West Indies" or "Middle East" or "South America" or "Latin America" or "Central America" or Afghanistan or Albania or Algeria or Angola or Argentina or Armenia or Armenian or Azerbaijan or Bangladesh or Benin or Byelarus or Byelorussian or Belarus or Belorussian or Belorussia or Belize or Bhutan or Bolivia or Bosnia or Herzegovina or Hercegovina or Botswana or Brazil or Bulgaria or "Burkina Faso" or "Burkina Fasso" or "Upper Volta" or Burundi or Urundi or Cambodia or "Khmer Republic" or Kampuchea or Cameroon or Cameroons or Cameron or Camerons or "Cape Verde" or "Central African Republic" or Chad or China or Colombia or Comoros or "Comoro Islands" or Comores or Mayotte or Congo or Zaire or "Costa Rica" or "Cote d'Ivoire" or "Ivory Coast" or Cuba or Djibouti or "French Somaliland" or Dominica or "Dominican Republic" or "East Timor" or "East Timur" or "Timor Leste" or Ecuador or Egypt or "United Arab Republic" or "El Salvador" or Eritrea or Ethiopia or Fiji or Gabon or Gabonese Republic or Gambia or Gaza or Georgia Republic or Georgian Republic or Ghana or Grenada or Guatemala or Guinea or Guiana or Guyana or Haiti or Honduras or India or Maldives or Indonesia or Iran or Iraq or Jamaica or Jordan or Kazakhstan or Kazakh or Kenya or Kiribati or Korea or Kosovo or Kyrgyzstan or Kirghizia or "Kyrgyz Republic" or Kirghiz or Kirgizstan or "Lao PDR" or Laos or Lebanon or Lesotho or Basutoland or Liberia or Libya or Macedonia or Madagascar or "Malagasy Republic" or Malaysia or Malaya or Malay or Sabah or Sarawak or Malawi or Mali or "Marshall Islands" or Mauritania or Mauritius or "Agalega Islands" or Mexico or Micronesia or "Middle East" or Moldova or Moldovia or Moldovian or Mongolia or Montenegro or Morocco or Ifni or Mozambique or Myanmar or Myanma or Burma or Namibia or Nepal or "Netherlands Antilles" or Nicaragua or Niger or Nigeria or Muscat or Pakistan or Palau or Palestine or Panama or Paraguay or Peru or Philippines or Philipines or Phillipines or Phillippines or "Papua New Guinea" or Romania or Rumania or Roumania or Rwanda or Ruanda or "Saint Lucia" or "St Lucia" or "Saint Vincent" or "St Vincent" or Grenadines or Samoa or "Samoan Islands" or "Navigator Island" or "Navigator Islands" or "Sao Tome" or Senegal or Serbia or Montenegro or Seychelles or "Sierra Leone" or "Sri Lanka" or "Solomon Islands" or Somalia or Sudan or Suriname or Surinam or Swaziland or "South Africa" or Syria or Tajikistan or Tadzhikistan or Tadjikistan or Tadzhik or Tanzania or Thailand or Togo or "Togolese Republic" or Tonga or Tunisia or Turkey or Turkmenistan or Turkmen or Uganda or Ukraine or Uzbekistan or Uzbek or Vanuatu or "New Hebrides" or Venezuela or Vietnam or "Viet Nam" or "West Bank" or Yemen or Zambia or Zimbabwe) not ("African-American*" or "African American*" or "Mexican American*" or "American Indian*" or "Asian American*" or "native american*" or "south korea*")).ti,ab. **1545875**

27 exp africa/ or algeria/ or egypt/ or libya/ or morocco/ or tunisia/ or cameroon/ or central african republic/ or chad/ or congo/ or "democratic republic of the congo"/ or equatorial guinea/ or gabon/ or burundi/ or djibouti/ or eritrea/ or ethiopia/ or kenya/ or rwanda/ or somalia/ or south sudan/ or sudan/ or tanzania/ or uganda/ or angola/ or botswana/ or lesotho/ or malawi/ or mozambique/ or namibia/ or south africa/ or swaziland/ or zambia/ or zimbabwe/ or benin/ or burkina faso/ or cape verde/ or cote d'ivoire/ or gambia/ or ghana/ or guinea/ or guinea-bissau/ or liberia/ or mali/ or mauritania/ or niger/ or nigeria/ or senegal/ or sierra leone/ or togo/ or americas/ or exp caribbean region/ or exp west indies/ or exp central america/ or belize/ or costa rica/ or el salvador/ or guatemala/ or honduras/ or nicaragua/ or panama/ or panama canal zone/ or latin america/ or mexico/ or exp south america/ or argentina/ or bolivia/ or brazil/ or chile/ or colombia/ or ecuador/ or french guiana/ or guyana/ or paraguay/ or peru/ or suriname/ or uruguay/ or venezuela/ or asia/ or asia, central/ or kazakhstan/ or kyrgyzstan/ or tajikistan/ or turkmenistan/ or uzbekistan/ or exp asia, southeastern/ or borneo/ or brunei/ or cambodia/ or timor-leste/ or indonesia/ or laos/ or malaysia/ or mekong valley/ or myanmar/ or philippines/ or singapore/ or thailand/ or vietnam/ or asia, western/ or bangladesh/ or bhutan/ or india/ or sikkim/ or middle east/ or afghanistan/ or bahrain/ or iran/ or iraq/ or israel/ or jordan/ or kuwait/ or lebanon/ or oman/ or qatar/ or saudi arabia/ or syria/ or turkey/ or united arab emirates/ or yemen/ or nepal/ or pakistan/ or sri lanka/ or far east/ or china/ or beijing/ or macau/ or tibet/ or korea/ or mongolia/ or taiwan/ or indian ocean islands/ or comoros/ or madagascar/ or mauritius/ or reunion/ or seychelles/ or pacific islands/ or exp melanesia/ or exp micronesia/ or polynesia/ or pitcairn island/ or exp samoa/ or tonga/ or prince edward island/ or west indies/ or "antigua and barbuda"/ or bahamas/ or barbados/ or cuba/ or dominica/ or dominican republic/ or grenada/ or guadeloupe/ or haiti/ or jamaica/ or martinique/ or netherlands antilles/ or puerto rico/ or "saint kitts and nevis"/ or saint lucia/ or "saint vincent and the grenadines"/ or "trinidad and tobago"/ or united states virgin islands/ or oceania/ **1516242**

28 ((developing or less* developed or under developed or underdeveloped or middle income or low* income or underserved or under served or deprived or poor*) adj (countr* or nation? or population? or world or state*)).ti,ab. **139716**

29 ((developing or less* developed or under developed or underdeveloped or middle income or low* income) adj (economy or economies)).ti,ab. **824**

30 (low* adj (gdp or gnp or gross domestic or gross national)).tw. **400**

31 (low adj3 middle adj3 countr*).tw. **23429**

32 (lmic or lmics or third world or lami countr*).tw. **10937**

33 transitional countr*.tw. **239**

34 exp developing country/ **96051**

35 24 or 25 or 26 or 27 or 28 or 29 or 30 or 31 or 32 or 33 or 34 **2165764**

36 20 or 21 or 22 or 23 **15995**

37 35 and 36 **800**

International Pharmaceutical Abstracts <1970 to May 2021>

1 "resource poor".mp. [mp=title, subject heading word, registry word, abstract, trade name/generic name] **79**

2 ((Africa or Asia or Caribbean or "West Indies" or "Middle East" or "South America" or "Latin America" or "Central America" or Afghanistan or Albania or Algeria or Angola or Argentina or Armenia or Armenian or Azerbaijan or Bangladesh or Benin or Byelarus or Byelorussian or Belarus or Belorussian or Belorussia or Belize or Bhutan or Bolivia or Bosnia or Herzegovina or Hercegovina or Botswana or Brazil or Bulgaria or "Burkina Faso" or "Burkina Fasso" or "Upper Volta" or Burundi or Urundi or Cambodia or "Khmer Republic" or Kampuchea or Cameroon or Cameroons or Cameron or Camerons or "Cape Verde" or "Central African Republic" or Chad or China or Colombia or Comoros or "Comoro Islands" or Comores or Mayotte or Congo or Zaire or "Costa Rica" or "Cote d'Ivoire" or "Ivory Coast" or Cuba or Djibouti or "French Somaliland" or Dominica or "Dominican Republic" or "East Timor" or "East Timur" or "Timor Leste" or Ecuador or Egypt or "United Arab Republic" or "El Salvador" or Eritrea or Ethiopia or Fiji or Gabon or Gabonese Republic or Gambia or Gaza or Georgia Republic or Georgian Republic or Ghana or Grenada or Guatemala or Guinea or Guiana or Guyana or Haiti or Honduras or India or Maldives or Indonesia or Iran or Iraq or Jamaica or Jordan or Kazakhstan or Kazakh or Kenya or Kiribati or Korea or Kosovo or Kyrgyzstan or Kirghizia or "Kyrgyz Republic" or Kirghiz or Kirgizstan or "Lao PDR" or Laos or Lebanon or Lesotho or Basutoland or Liberia or Libya or Macedonia or Madagascar or "Malagasy Republic" or Malaysia or Malaya or Malay or Sabah or Sarawak or Malawi or Mali or "Marshall Islands" or Mauritania or Mauritius or "Agalega Islands" or Mexico or Micronesia or "Middle East" or Moldova or Moldovia or Moldovian or Mongolia or Montenegro or Morocco or Ifni or Mozambique or Myanmar or Myanma or Burma or Namibia or Nepal or "Netherlands Antilles" or Nicaragua or Niger or Nigeria or Muscat or Pakistan or Palau or Palestine or Panama or Paraguay or Peru or Philippines or Philipines or Phillipines or Phillippines or "Papua New Guinea" or Romania or Rumania or Roumania or Rwanda or Ruanda or "Saint Lucia" or "St Lucia" or "Saint Vincent" or "St Vincent" or Grenadines or Samoa or "Samoan Islands" or "Navigator Island" or "Navigator Islands" or "Sao Tome" or Senegal or Serbia or Montenegro or Seychelles or "Sierra Leone" or "Sri Lanka" or "Solomon Islands" or Somalia or Sudan or Suriname or Surinam or Swaziland or "South Africa" or Syria or Tajikistan or Tadzhikistan or Tadjikistan or Tadzhik or Tanzania or Thailand or Togo or "Togolese Republic" or Tonga or Tunisia or Turkey or Turkmenistan or Turkmen or Uganda or Ukraine or Uzbekistan or Uzbek or Vanuatu or "New Hebrides" or Venezuela or Vietnam or "Viet Nam" or "West Bank" or Yemen or Zambia or Zimbabwe) not ("African-American*" or "African American*" or "Mexican American*" or "American Indian*" or "Asian American*" or "native american*" or "south korea*")).ti,ab. **21987**

3 ((developing or less* developed or under developed or underdeveloped or middle income or low* income or underserved or under served or deprived or poor*) adj (countr* or nation? or population? or world or state*)).ti,ab. **1679**

4 ((developing or less* developed or under developed or underdeveloped or middle income or low* income) adj (economy or economies)).ti,ab. **3**

5 (low* adj (gdp or gnp or gross domestic or gross national)).tw. **2**

6 (low adj3 middle adj3 countr*).tw. **179**

7 (lmic or lmics or third world or lami countr*).tw.  **159**

8 transitional countr*.tw. **5**

9 Developing Countries.sh. **1791**

10 1 or 2 or 3 or 4 or 5 or 6 or 7 or 8 or 9 **23674**

11 (((electronic or computeri$ed or digital) adj5 prescri*) or e-prescri*).tw. **588**

12 (barcode adj10 (medic* or drug*)).tw. **106**

13 (barcode adj10 dispens*).tw. **31**

14 ((digital or electronic or computeri$ed) adj5 dispens*).tw. **60**

15 ((digital or electronic or computeri$ed) and ((medic* or drug*) adj3 administration record*)).tw. **91**

16 ((digital or electronic or computeri$ed) adj5 medication record*).tw. **8**

17 ((digital or electronic or computeri$ed) and ((medic* or drug*) adj2 management)).tw. **204**

18 "computerised physician order entry".tw. **14**

19 "computerized physician order entry".tw. **292**

20 "computerized provider order entry".tw. **124**

21 CPOE.tw. **468**

22 11 or 12 or 13 or 14 or 15 or 16 or 17 or 18 or 19 or 20 or 21 **1553**

23 10 and 22 **26**

CENTRAL

Search Name: LMIC MM 280521

Date Run: 28/05/2021 14:28:27

ID Search Hits

#1 MeSH descriptor: [Drug Therapy, Computer-Assisted] this term only **151**

#2 MeSH descriptor: [Electronic Prescribing] this term only **23**

#3 MeSH descriptor: [Drug Information Services] explode all trees **148**

#4 MeSH descriptor: [Medication Therapy Management] this term only **131**

#5 ((Africa or Asia or Caribbean or "West Indies" or "Middle East" or "South America" or "Latin America" or "Central America" or Afghanistan or Albania or Algeria or Angola or Argentina or Armenia or Armenian or Azerbaijan or Bangladesh or Benin or Byelarus or Byelorussian or Belarus or Belorussian or Belorussia or Belize or Bhutan or Bolivia or Bosnia or Herzegovina or Hercegovina or Botswana or Brazil or Bulgaria or "Burkina Faso" or "Burkina Fasso" or "Upper Volta" or Burundi or Urundi or Cambodia or "Khmer Republic" or Kampuchea or Cameroon or Cameroons or Cameron or Camerons or "Cape Verde" or "Central African Republic" or Chad or China or Colombia or Comoros or "Comoro Islands" or Comores or Mayotte or Congo or Zaire or "Costa Rica" or "Cote d'Ivoire" or "Ivory Coast" or Cuba or Djibouti or "French Somaliland" or Dominica or "Dominican Republic" or "East Timor" or "East Timur" or "Timor Leste" or Ecuador or Egypt or "United Arab Republic" or "El Salvador" or Eritrea or Ethiopia or Fiji or Gabon or Gabonese Republic or Gambia or Gaza or Georgia Republic or Georgian Republic or Ghana or Grenada or Guatemala or Guinea or Guiana or Guyana or Haiti or Honduras or India or Maldives or Indonesia or Iran or Iraq or Jamaica or Jordan or Kazakhstan or Kazakh or Kenya or Kiribati or Korea or Kosovo or Kyrgyzstan or Kirghizia or "Kyrgyz Republic" or Kirghiz or Kirgizstan or "Lao PDR" or Laos or Lebanon or Lesotho or Basutoland or Liberia or Libya or Macedonia or Madagascar or "Malagasy Republic" or Malaysia or Malaya or Malay or Sabah or Sarawak or Malawi or Mali or "Marshall Islands" or Mauritania or Mauritius or "Agalega Islands" or Mexico or Micronesia or "Middle East" or Moldova or Moldovia or Moldovian or Mongolia or Montenegro or Morocco or Ifni or Mozambique or Myanmar or Myanma or Burma or Namibia or Nepal or "Netherlands Antilles" or Nicaragua or Niger or Nigeria or Muscat or Pakistan or Palau or Palestine or Panama or Paraguay or Peru or Philippines or Philipines or Phillipines or Phillippines or "Papua New Guinea" or Romania or Rumania or Roumania or Rwanda or Ruanda or "Saint Lucia" or "St Lucia" or "Saint Vincent" or "St Vincent" or Grenadines or Samoa or "Samoan Islands" or "Navigator Island" or "Navigator Islands" or "Sao Tome" or Senegal or Serbia or Montenegro or Seychelles or "Sierra Leone" or "Sri Lanka" or "Solomon Islands" or Somalia or Sudan or Suriname or Surinam or Swaziland or "South Africa" or Syria or Tajikistan or Tadzhikistan or Tadjikistan or Tadzhik or Tanzania or Thailand or Togo or "Togolese Republic" or Tonga or Tunisia or Turkey or Turkmenistan or Turkmen or Uganda or Ukraine or Uzbekistan or Uzbek or Vanuatu or "New Hebrides" or Venezuela or Vietnam or "Viet Nam" or "West Bank" or Yemen or Zambia or Zimbabwe) not ("African-American*" or "African American*" or "Mexican American*" or "American Indian*" or "Asian American*" or "native american*" or "south korea*")):ti,ab,kw **87125**

#6 (low* near/1 (gdp or gnp or "gross domestic" or "gross national")):ti,ab **49**

#7 (low near/3 middle near/3 countr*):ti,ab **1554**

#8 (lmic or lmics or "third world" or "lami countr*" or "transitional countr*" or "resource poor"):ti,ab **960**

#9 MeSH descriptor: [Developing Countries] this term only **870**

#10 [mh Africa] or [mh Asia] or [mh Caribbean] or [mh "West Indies"] or [mh "Middle East"] or

[mh "South America"] or [mh "Latin America"] or [mh "Central America"] **30585**

#11 (((electronic or computeri* or digital) near/5 prescri*) or "e-prescri*"):ti,kw,ab **300**

#12 (barcode near/10 (medic* or drug*)):ti,kw,ab **16**

#13 (barcode near/10 dispens*):ti,kw,ab **3**

#14 ((digital or electronic or computeri*) near/5 dispens*):ti,kw,ab **62**

#15 ((digital or electronic or computeri*) and ((medic* of drug*) near/3 administration record*)):ti,kw,ab **903**

#16 ((digital or electronic or computeri*) near/5 medication record*):ti,kw,ab **205**

#17 ((digital or electronic or computeri*) and ((medic* or drug*) near/3 management)):ti,kw,ab **498**

#18 "computerised physician order entry":ti,kw,ab **54**

#19 "computerised provider order entry":ti,kw,ab **51**

#20 CPOE:ti,kw,ab **50**

#21 (EMR near/10 (medicine* or drug* or prescri* or dispens*)):ti,ab **48**

#22 (electronic medical record* near/10 (medicine* or drug* or prescri* or dispens*)):ti,ab **226**

#23 (computerized health record* near/10 (medicine* or drug* or prescri* or dispens*)):ti,ab **38**

#24 (computerised health record* near/10 (medicine* or drug* or prescri* or dispens*)):ti,ab **38**

#25 (EHR near/10 (medicine* or drug* or prescri* or dispens*)):ti,ab **64**

#26 (electronic health record* near/10 (medicine* or drug* or prescri* or dispens*)):ti,ab **222**

#27 {or #1-#10} **94795**

#28 {or #11-#26} **2162**

#29 #27 and #28 **199**

CINAHL

| **#** | **Query** | **Limiters/Expanders** | **Results** |
| --- | --- | --- | --- |
| S30 | S14 AND S29 | Expanders - Apply equivalent subjects Search modes - Boolean/Phrase | 388 |
| S29 | S1 OR S15 OR S16 OR S17 OR S18 OR S19 OR S20 OR S21 OR S22 OR S23 OR S24 OR S25 OR S26 OR S27 OR S28 | Expanders - Apply equivalent subjects Search modes - Boolean/Phrase | 9,468 |
| S28 | (MM "Drug Therapy, Computer Assisted") | Expanders - Apply equivalent subjects Search modes - Boolean/Phrase | 321 |
| S27 | TI (CPOE) OR AB (CPOE) | Expanders - Apply equivalent subjects Search modes - Boolean/Phrase | 2 |
| S26 | TI "computerized provider order entry" OR AB "computerized provider order entry" | Expanders - Apply equivalent subjects Search modes - Boolean/Phrase | 293 |
| S25 | TI "computerised provider order entry" OR AB "computerised provider order entry" | Expanders - Apply equivalent subjects Search modes - Boolean/Phrase | 23 |
| S24 | TI "computerized physician order entry" OR AB "computerized physician order entry" | Expanders - Apply equivalent subjects Search modes - Boolean/Phrase | 473 |
| S23 | TI "computerised physician order entry" OR AB "computerised physician order entry" | Expanders - Apply equivalent subjects Search modes - Boolean/Phrase | 46 |
| S22 | TI ((digital or electronic or computeri*) and medicine* and management) OR AB ((digital or electronic or computeri*) and medicine* and management) | Expanders - Apply equivalent subjects Search modes - Boolean/Phrase | 866 |
| S21 | TI ((digital or electronic or computeri*) and medication record*) OR AB ((digital or electronic or computeri*) and medication record*) | Expanders - Apply equivalent subjects Search modes - Boolean/Phrase | 615 |
| S20 | TI ((digital or electronic or computeri*) and medic* and administration record*) OR AB ((digital or electronic or computeri*) and medic* and administration record*) | Expanders - Apply equivalent subjects Search modes - Boolean/Phrase | 242 |
| S19 | TI ((digital or electronic or computeri*) and dispens*) OR AB ((digital or electronic or computeri*) and dispens*) | Expanders - Apply equivalent subjects Search modes - Boolean/Phrase | 683 |
| S18 | TI (barcode and dispens*) OR AB (barcode and dispens*) | Expanders - Apply equivalent subjects Search modes - Boolean/Phrase | 43 |
| S17 | TI (barcode and medic*) OR AB (barcode and medic*) | Expanders - Apply equivalent subjects Search modes - Boolean/Phrase | 256 |
| S16 | TI ((electronic or computeri* or digital) AND prescri*) OR AB ((electronic or computeri* or digital) AND prescri*) | Expanders - Apply equivalent subjects Search modes - Boolean/Phrase | 5,682 |
| S15 | TI (e-prescribing or "electronic prescribing") OR AB (e-prescribing or "electronic prescribing") | Expanders - Apply equivalent subjects Search modes - Boolean/Phrase | 882 |
| S14 | S2 OR S3 OR S4 OR S5 OR S6 OR S7 OR S8 OR S9 OR S10 OR S11 OR S12 OR S13 | Expanders - Apply equivalent subjects Search modes - Boolean/Phrase | 323,448 |
| S13 | TI "middle income" OR AB "middle income" | Expanders - Apply equivalent subjects Search modes - Boolean/Phrase | 10,638 |
| S12 | TI "low income" OR AB "low income" | Expanders - Apply equivalent subjects Search modes - Boolean/Phrase | 20,817 |
| S11 | TI ((Africa or Asia or Caribbean or "West Indies" or "Middle East" or "South America" or "Latin America" or "Central America" or Afghanistan or Albania or Algeria or Angola or Argentina or Armenia or Armenian or Azerbaijan or Bangladesh or Benin or Byelarus or Byelorussian or Belarus or Belorussian or Belorussia or Belize or Bhutan or Bolivia or Bosnia or Herzegovina or Hercegovina or Botswana or Brazil or Bulgaria or "Burkina Faso" or "Burkina Fasso" or "Upper Volta" or Burundi or Urundi or Cambodia or "Khmer Republic" or Kampuchea or Cameroon or Cameroons or Cameron or Camerons or "Cape Verde" or "Central African Republic" or Chad or China or Colombia or Comoros or "Comoro Islands" or Comores or Mayotte or Congo or Zaire or "Costa Rica" or "Cote d'Ivoire" or "Ivory Coast" or Cuba or Djibouti or "French Somaliland" or Dominica or "Dominican Republic" or "East Timor" or "East Timur" or "Timor Leste" or Ecuador or Egypt or "United Arab Republic" or "El Salvador" or Eritrea or Ethiopia or Fiji or Gabon or Gabonese Republic or Gambia or Gaza or Georgia Republic or Georgian Republic or Ghana or Grenada or Guatemala or Guinea or Guiana or Guyana or Haiti or Honduras or India or Maldives or Indonesia or Iran or Iraq or Jamaica or Jordan or Kazakhstan or Kazakh or Kenya or Kiribati or Korea or Kosovo or Kyrgyzstan or Kirghizia or "Kyrgyz Republic" or Kirghiz or Kirgizstan or "Lao PDR" or Laos or Lebanon or Lesotho or Basutoland or Liberia or Libya or Macedonia or Madagascar or "Malagasy Republic" or Malaysia or Malaya or Malay or Sabah or Sarawak or Malawi or Mali or "Marshall Islands" or Mauritania or Mauritius or "Agalega Islands" or Mexico or Micronesia or "Middle East" or Moldova or Moldovia or Moldovian or Mongolia or Montenegro or Morocco or Ifni or Mozambique or Myanmar or Myanma or Burma or Namibia or Nepal or "Netherlands Antilles" or Nicaragua or Niger or Nigeria or Muscat or Pakistan or Palau or Palestine or Panama or Paraguay or Peru or Philippines or Philipines or Phillipines or Phillippines or "Papua New Guinea" or Romania or Rumania or Roumania or Rwanda or Ruanda or "Saint Lucia" or "St Lucia" or "Saint Vincent" or "St Vincent" or Grenadines or Samoa or "Samoan Islands" or "Navigator Island" or "Navigator Islands" or "Sao Tome" or Senegal or Serbia or Montenegro or Seychelles or "Sierra Leone" or "Sri Lanka" or "Solomon Islands" or Somalia or Sudan or Suriname or Surinam or Swaziland or "South Africa" or Syria or Tajikistan or Tadzhikistan or Tadjikistan or Tadzhik or Tanzania or Thailand or Togo or "Togolese Republic" or Tonga or Tunisia or Turkey or Turkmenistan or Turkmen or Uganda or Ukraine or Uzbekistan or Uzbek or Vanuatu or "New Hebrides" or Venezuela or Vietnam or "Viet Nam" or "West Bank" or Yemen or Zambia or Zimbabwe) not ("African-American*" or "African American*" or "Mexican American*" or "American Indian*" or "Asian American*" or "native american*" or "south korea*")) OR AB ((Africa or Asia or Caribbean or "West Indies" or "Middle East" or "South America" or "Latin America" or "Central America" or Afghanistan or Albania or Algeria or Angola or Argentina or Armenia or Armenian or Azerbaijan or Bangladesh or Benin or Byelarus or Byelorussian or Belarus or Belorussian or Belorussia or Belize or Bhutan or Bolivia or Bosnia or Herzegovina or Hercegovina or Botswana or Brazil or Bulgaria or "Burkina Faso" or "Burkina Fasso" or "Upper Volta" or Burundi or Urundi or Cambodia or "Khmer Republic" or Kampuchea or Cameroon or Cameroons or Cameron or Camerons or "Cape Verde" or "Central African Republic" or Chad or China or Colombia or Comoros or "Comoro Islands" or Comores or Mayotte or Congo or Zaire or "Costa Rica" or "Cote d'Ivoire" or "Ivory Coast" or Cuba or Djibouti or "French Somaliland" or Dominica or "Dominican Republic" or "East Timor" or "East Timur" or "Timor Leste" or Ecuador or Egypt or "United Arab Republic" or "El Salvador" or Eritrea or Ethiopia or Fiji or Gabon or Gabonese Republic or Gambia or Gaza or Georgia Republic or Georgian Republic or Ghana or Grenada or Guatemala or Guinea or Guiana or Guyana or Haiti or Honduras or India or Maldives or Indonesia or Iran or Iraq or Jamaica or Jordan or Kazakhstan or Kazakh or Kenya or Kiribati or Korea or Kosovo or Kyrgyzstan or Kirghizia or "Kyrgyz Republic" or Kirghiz or Kirgizstan or "Lao PDR" or Laos or Lebanon or Lesotho or Basutoland or Liberia or Libya or Macedonia or Madagascar or "Malagasy Republic" or Malaysia or Malaya or Malay or Sabah or Sarawak or Malawi or Mali or "Marshall Islands" or Mauritania or Mauritius or "Agalega Islands" or Mexico or Micronesia or "Middle East" or Moldova or Moldovia or Moldovian or Mongolia or Montenegro or Morocco or Ifni or Mozambique or Myanmar or Myanma or Burma or Namibia or Nepal or "Netherlands Antilles" or Nicaragua or Niger or Nigeria or Muscat or Pakistan or Palau or Palestine or Panama or Paraguay or Peru or Philippines or Philipines or Phillipines or Phillippines or "Papua New Guinea" or Romania or Rumania or Roumania or Rwanda or Ruanda or "Saint Lucia" or "St Lucia" or "Saint Vincent" or "St Vincent" or Grenadines or Samoa or "Samoan Islands" or "Navigator Island" or "Navigator Islands" or "Sao Tome" or Senegal or Serbia or Montenegro or Seychelles or "Sierra Leone" or "Sri Lanka" or "Solomon Islands" or Somalia or Sudan or Suriname or Surinam or Swaziland or "South Africa" or Syria or Tajikistan or Tadzhikistan or Tadjikistan or Tadzhik or Tanzania or Thailand or Togo or "Togolese Republic" or Tonga or Tunisia or Turkey or Turkmenistan or Turkmen or Uganda or Ukraine or Uzbekistan or Uzbek or Vanuatu or "New Hebrides" or Venezuela or Vietnam or "Viet Nam" or "West Bank" or Yemen or Zambia or Zimbabwe) not ("African-American*" or "African American*" or "Mexican American*" or "American Indian*" or "Asian American*" or "native american*" or "south korea*")) | Expanders - Apply equivalent subjects Search modes - Boolean/Phrase | 290,983 |
| S10 | TI "resource poor" OR AB "resource poor" | Expanders - Apply equivalent subjects Search modes - Boolean/Phrase | 1,812 |
| S9 | TI (third world countr*) OR AB (third world countr*) | Expanders - Apply equivalent subjects Search modes - Boolean/Phrase | 233 |
| S8 | TI (less developed countr*) OR AB (less developed countr*) | Expanders - Apply equivalent subjects Search modes - Boolean/Phrase | 428 |
| S7 | TI (underdeveloped countr*) OR AB (underdeveloped countr*) | Expanders - Apply equivalent subjects Search modes - Boolean/Phrase | 232 |
| S6 | TI (LMI) OR AB (LMI) | Expanders - Apply equivalent subjects Search modes - Boolean/Phrase | 140 |
| S5 | TI (LMIC) OR AB (LMIC) | Expanders - Apply equivalent subjects Search modes - Boolean/Phrase | 2,366 |
| S4 | TI (transitional countr*) OR AB (transitional countr*) | Expanders - Apply equivalent subjects Search modes - Boolean/Phrase | 111 |
| S3 | TI (developing countr*) OR AB (developing countr*) | Expanders - Apply equivalent subjects Search modes - Boolean/Phrase | 16,204 |
| S2 | (MM "Developing Countries") OR (MM "Low and Middle Income Countries") | Expanders - Apply equivalent subjects Search modes - Boolean/Phrase | 6,783 |
| S1 | (MM "Electronic Order Entry") | Expanders - Apply equivalent subjects Search modes - Boolean/Phrase | 1,858 |

Web of Science

| **Set** | **Results** | **Save History / Create AlertOpen Saved History** |
| --- | --- | --- |
| # 22 | [**1,118**](https://apps-webofknowledge-com.libproxy.ucl.ac.uk/summary.do?product=WOS&doc=1&qid=37&SID=C2GljNlVrqt3Wd674te&search_mode=CombineSearches&update_back2search_link_param=yes) | #21 AND #10  *Indexes=SCI-EXPANDED, SSCI, A&HCI, CPCI-S, CPCI-SSH, BKCI-S, BKCI-SSH, ESCI, CCR-EXPANDED, IC Timespan=All years* |
| # 21 | [**7,138**](https://apps-webofknowledge-com.libproxy.ucl.ac.uk/summary.do?product=WOS&doc=1&qid=33&SID=C2GljNlVrqt3Wd674te&search_mode=CombineSearches&update_back2search_link_param=yes) | #20 OR #19 OR #18 OR #17 OR #16 OR #15 OR #14 OR #13 OR #12 OR #11  *Indexes=SCI-EXPANDED, SSCI, A&HCI, CPCI-S, CPCI-SSH, BKCI-S, BKCI-SSH, ESCI, CCR-EXPANDED, IC Timespan=All years* |
| # 20 | [**996**](https://apps-webofknowledge-com.libproxy.ucl.ac.uk/summary.do?product=WOS&doc=1&qid=20&SID=C2GljNlVrqt3Wd674te&search_mode=AdvancedSearch&update_back2search_link_param=yes) | TS= CPOE  *Indexes=SCI-EXPANDED, SSCI, A&HCI, CPCI-S, CPCI-SSH, BKCI-S, BKCI-SSH, ESCI, CCR-EXPANDED, IC Timespan=All years* |
| # 19 | [**420**](https://apps-webofknowledge-com.libproxy.ucl.ac.uk/summary.do?product=WOS&doc=1&qid=19&SID=C2GljNlVrqt3Wd674te&search_mode=AdvancedSearch&update_back2search_link_param=yes) | TS="computeri* provider order entry"  *Indexes=SCI-EXPANDED, SSCI, A&HCI, CPCI-S, CPCI-SSH, BKCI-S, BKCI-SSH, ESCI, CCR-EXPANDED, IC Timespan=All years* |
| # 18 | [**906**](https://apps-webofknowledge-com.libproxy.ucl.ac.uk/summary.do?product=WOS&doc=1&qid=18&SID=C2GljNlVrqt3Wd674te&search_mode=AdvancedSearch&update_back2search_link_param=yes) | TS="computeri* physician order entry"  *Indexes=SCI-EXPANDED, SSCI, A&HCI, CPCI-S, CPCI-SSH, BKCI-S, BKCI-SSH, ESCI, CCR-EXPANDED, IC Timespan=All years* |
| # 17 | [**2,007**](https://apps-webofknowledge-com.libproxy.ucl.ac.uk/summary.do?product=WOS&doc=1&qid=23&SID=C2GljNlVrqt3Wd674te&search_mode=AdvancedSearch&update_back2search_link_param=yes) | TS=((digital or electronic or computeri*) and ((medic* or drug*) near/2 management))  *Indexes=SCI-EXPANDED, SSCI, A&HCI, CPCI-S, CPCI-SSH, BKCI-S, BKCI-SSH, ESCI, CCR-EXPANDED, IC Timespan=All years* |
| # 16 | [**41**](https://apps-webofknowledge-com.libproxy.ucl.ac.uk/summary.do?product=WOS&doc=1&qid=16&SID=C2GljNlVrqt3Wd674te&search_mode=AdvancedSearch&update_back2search_link_param=yes) | TS=((digital or electronic or computeri*) near/5 "medication record*")  *Indexes=SCI-EXPANDED, SSCI, A&HCI, CPCI-S, CPCI-SSH, BKCI-S, BKCI-SSH, ESCI, CCR-EXPANDED, IC Timespan=All years* |
| # 15 | [**139**](https://apps-webofknowledge-com.libproxy.ucl.ac.uk/summary.do?product=WOS&doc=1&qid=26&SID=C2GljNlVrqt3Wd674te&search_mode=AdvancedSearch&update_back2search_link_param=yes) | TS=((digital or electronic or computeri*) and ((medic* or drug*) near/3 "administration record*"))  *Indexes=SCI-EXPANDED, SSCI, A&HCI, CPCI-S, CPCI-SSH, BKCI-S, BKCI-SSH, ESCI, CCR-EXPANDED, IC Timespan=All years* |
| # 14 | [**439**](https://apps-webofknowledge-com.libproxy.ucl.ac.uk/summary.do?product=WOS&doc=1&qid=14&SID=C2GljNlVrqt3Wd674te&search_mode=AdvancedSearch&update_back2search_link_param=yes) | TS=((digital or electronic or computeri*) near/5 dispens*)  *Indexes=SCI-EXPANDED, SSCI, A&HCI, CPCI-S, CPCI-SSH, BKCI-S, BKCI-SSH, ESCI, CCR-EXPANDED, IC Timespan=All years* |
| # 13 | [**25**](https://apps-webofknowledge-com.libproxy.ucl.ac.uk/summary.do?product=WOS&doc=1&qid=13&SID=C2GljNlVrqt3Wd674te&search_mode=AdvancedSearch&update_back2search_link_param=yes) | TS=(barcode near/10 dispens*)  *Indexes=SCI-EXPANDED, SSCI, A&HCI, CPCI-S, CPCI-SSH, BKCI-S, BKCI-SSH, ESCI, CCR-EXPANDED, IC Timespan=All years* |
| # 12 | [**330**](https://apps-webofknowledge-com.libproxy.ucl.ac.uk/summary.do?product=WOS&doc=1&qid=29&SID=C2GljNlVrqt3Wd674te&search_mode=AdvancedSearch&update_back2search_link_param=yes) | TS=(barcode near/10 (medic* or drug*) )  *Indexes=SCI-EXPANDED, SSCI, A&HCI, CPCI-S, CPCI-SSH, BKCI-S, BKCI-SSH, ESCI, CCR-EXPANDED, IC Timespan=All years* |
| # 11 | [**3,163**](https://apps-webofknowledge-com.libproxy.ucl.ac.uk/summary.do?product=WOS&doc=1&qid=32&SID=C2GljNlVrqt3Wd674te&search_mode=AdvancedSearch&update_back2search_link_param=yes) | TS=(((electronic or computeri* or digital) near/5 prescri*) or e-prescri*)  *Indexes=SCI-EXPANDED, SSCI, A&HCI, CPCI-S, CPCI-SSH, BKCI-S, BKCI-SSH, ESCI, CCR-EXPANDED, IC Timespan=All years* |
| # 10 | [**17,294,393**](https://apps-webofknowledge-com.libproxy.ucl.ac.uk/summary.do?product=WOS&doc=1&qid=36&SID=C2GljNlVrqt3Wd674te&search_mode=CombineSearches&update_back2search_link_param=yes) | #9 OR #8 OR #7 OR #6 OR #5 OR #4 OR #3 OR #2 OR #1  *Indexes=SCI-EXPANDED, SSCI, A&HCI, CPCI-S, CPCI-SSH, BKCI-S, BKCI-SSH, ESCI, CCR-EXPANDED, IC Timespan=All years* |
| # 9 | [**527**](https://apps-webofknowledge-com.libproxy.ucl.ac.uk/summary.do?product=WOS&doc=1&qid=9&SID=C2GljNlVrqt3Wd674te&search_mode=AdvancedSearch&update_back2search_link_param=yes) | TS=("transitional countr*")  *Indexes=SCI-EXPANDED, SSCI, A&HCI, CPCI-S, CPCI-SSH, BKCI-S, BKCI-SSH, ESCI, CCR-EXPANDED, IC Timespan=All years* |
| # 8 | [**24,508**](https://apps-webofknowledge-com.libproxy.ucl.ac.uk/summary.do?product=WOS&doc=1&qid=35&SID=C2GljNlVrqt3Wd674te&search_mode=AdvancedSearch&update_back2search_link_param=yes) | TS=(lmic or lmics or "third world" or "lami countr*" or "resource poor")  *Indexes=SCI-EXPANDED, SSCI, A&HCI, CPCI-S, CPCI-SSH, BKCI-S, BKCI-SSH, ESCI, CCR-EXPANDED, IC Timespan=All years* |
| # 7 | [**22,898**](https://apps-webofknowledge-com.libproxy.ucl.ac.uk/summary.do?product=WOS&doc=1&qid=7&SID=C2GljNlVrqt3Wd674te&search_mode=AdvancedSearch&update_back2search_link_param=yes) | TS=(low NEAR/3 middle NEAR/3 countr*)  *Indexes=SCI-EXPANDED, SSCI, A&HCI, CPCI-S, CPCI-SSH, BKCI-S, BKCI-SSH, ESCI, CCR-EXPANDED, IC Timespan=All years* |
| # 6 | [**751**](https://apps-webofknowledge-com.libproxy.ucl.ac.uk/summary.do?product=WOS&doc=1&qid=6&SID=C2GljNlVrqt3Wd674te&search_mode=AdvancedSearch&update_back2search_link_param=yes) | TS=(low* NEAR/1 (gdp or gnp or "gross domestic" or "gross national") )  *Indexes=SCI-EXPANDED, SSCI, A&HCI, CPCI-S, CPCI-SSH, BKCI-S, BKCI-SSH, ESCI, CCR-EXPANDED, IC Timespan=All years* |
| # 5 | [**275,595**](https://apps-webofknowledge-com.libproxy.ucl.ac.uk/summary.do?product=WOS&doc=1&qid=5&SID=C2GljNlVrqt3Wd674te&search_mode=AdvancedSearch&update_back2search_link_param=yes) | TS=((developing or "less* developed" or "under developed" or underdeveloped or "middle income" or "low* income" or underserved or "under served" or deprived or poor*) NEAR/1 (countr* or nation? or population? or world or state* or economy or economies) )  *Indexes=SCI-EXPANDED, SSCI, A&HCI, CPCI-S, CPCI-SSH, BKCI-S, BKCI-SSH, ESCI, CCR-EXPANDED, IC Timespan=All years* |
| # 4 | [**409,406**](https://apps-webofknowledge-com.libproxy.ucl.ac.uk/summary.do?product=WOS&doc=1&qid=4&SID=C2GljNlVrqt3Wd674te&search_mode=AdvancedSearch&update_back2search_link_param=yes) | CU=(Africa or Asia or Caribbean or "West Indies" or "Middle East" or "South America" or "Latin America" or "Central America")  *Indexes=SCI-EXPANDED, SSCI, A&HCI, CPCI-S, CPCI-SSH, BKCI-S, BKCI-SSH, ESCI, CCR-EXPANDED, IC Timespan=All years* |
| # 3 | [**15,762,658**](https://apps-webofknowledge-com.libproxy.ucl.ac.uk/summary.do?product=WOS&doc=1&qid=3&SID=C2GljNlVrqt3Wd674te&search_mode=AdvancedSearch&update_back2search_link_param=yes) | CU=(Afghanistan or Albania or Algeria or Angola or Argentina or Armenia or Armenian or Azerbaijan or Bangladesh or Benin or Byelarus or Byelorussian or Belarus or Belorussian or Belorussia or Belize or Bhutan or Bolivia or Bosnia or Herzegovina or Hercegovina or Botswana or Brazil or Bulgaria or "Burkina Faso" or "Burkina Fasso" or "Upper Volta" or Burundi or Urundi or Cambodia or "Khmer Republic" or Kampuchea or Cameroon or Cameroons or Cameron or Camerons or "Cape Verde" or "Central African Republic" or Chad or China or Colombia or Comoros or "Comoro Islands" or Comores or Mayotte or Congo or Zaire or "Costa Rica" or "Cote d'Ivoire" or "Ivory Coast" or Cuba or Djibouti or "French Somaliland" or Dominica or "Dominican Republic" or "East Timor" or "East Timur" or "Timor Leste" or Ecuador or Egypt or "United Arab Republic" or "El Salvador" or Eritrea or Ethiopia or Fiji or Gabon or "Gabonese Republic" or Gambia or Gaza or "Georgia Republic" or "Georgian Republic" or Ghana or Grenada or Guatemala or Guinea or Guiana or Guyana or Haiti or Honduras or India or Maldives or Indonesia or Iran or Iraq or Jamaica or Jordan or Kazakhstan or Kazakh or Kenya or Kiribati or Korea or Kosovo or Kyrgyzstan or Kirghizia or "Kyrgyz Republic" or Kirghiz or Kirgizstan or "Lao PDR" or Laos or Lebanon or Lesotho or Basutoland or Liberia or Libya or Macedonia or Madagascar or "Malagasy Republic" or Malaysia or Malaya or Malay or Sabah or Sarawak or Malawi or Mali or "Marshall Islands" or Mauritania or Mauritius or "Agalega Islands" or Mexico or Micronesia or "Middle East" or Moldova or Moldovia or Moldovian or Mongolia or Montenegro or Morocco or Ifni or Mozambique or Myanmar or Myanma or Burma or Namibia or Nepal or "Netherlands Antilles" or Nicaragua or Niger or Nigeria or Muscat or Pakistan or Palau or Palestine or Panama or Paraguay or Peru or Philippines or Philipines or Phillipines or Phillippines or "Papua New Guinea" or Romania or Rumania or Roumania or Rwanda or Ruanda or "Saint Lucia" or "St Lucia" or "Saint Vincent" or "St Vincent" or Grenadines or Samoa or "Samoan Islands" or "Navigator Island" or "Navigator Islands" or "Sao Tome" or Senegal or Serbia or Montenegro or Seychelles or "Sierra Leone" or "Sri Lanka" or "Solomon Islands" or Somalia or Sudan or Suriname or Surinam or Swaziland or "South Africa" or Syria or Tajikistan or Tadzhikistan or Tadjikistan or Tadzhik or Tanzania or Thailand or Togo or "Togolese Republic" or Tonga or Tunisia or Turkey or Turkmenistan or Turkmen or Uganda or Ukraine or Uzbekistan or Uzbek or Vanuatu or "New Hebrides" or Venezuela or Vietnam or "Viet Nam" or "West Bank" or Yemen or Zambia or Zimbabwe)  *Indexes=SCI-EXPANDED, SSCI, A&HCI, CPCI-S, CPCI-SSH, BKCI-S, BKCI-SSH, ESCI, CCR-EXPANDED, IC Timespan=All years* |
| # 2 | [**3,394,322**](https://apps-webofknowledge-com.libproxy.ucl.ac.uk/summary.do?product=WOS&doc=1&qid=2&SID=C2GljNlVrqt3Wd674te&search_mode=AdvancedSearch&update_back2search_link_param=yes) | TS=((Afghanistan or Albania or Algeria or Angola or Argentina or Armenia or Armenian or Azerbaijan or Bangladesh or Benin or Byelarus or Byelorussian or Belarus or Belorussian or Belorussia or Belize or Bhutan or Bolivia or Bosnia or Herzegovina or Hercegovina or Botswana or Brazil or Bulgaria or "Burkina Faso" or "Burkina Fasso" or "Upper Volta" or Burundi or Urundi or Cambodia or "Khmer Republic" or Kampuchea or Cameroon or Cameroons or Cameron or Camerons or "Cape Verde" or "Central African Republic" or Chad or China or Colombia or 8 Comoros or "Comoro Islands" or Comores or Mayotte or Congo or Zaire or "Costa Rica" or "Cote d'Ivoire" or "Ivory Coast" or Cuba or Djibouti or "French Somaliland" or Dominica or "Dominican Republic" or "East Timor" or "East Timur" or "Timor Leste" or Ecuador or Egypt or "United Arab Republic" or "El Salvador" or Eritrea or Ethiopia or Fiji or Gabon or "Gabonese Republic" or Gambia or Gaza or "Georgia Republic" or "Georgian Republic" or Ghana or Grenada or Guatemala or Guinea or Guiana or Guyana or Haiti or Honduras or India or Maldives or Indonesia or Iran or Iraq or Jamaica or Jordan or Kazakhstan or Kazakh or Kenya or Kiribati or Korea or Kosovo or Kyrgyzstan or Kirghizia or "Kyrgyz Republic" or Kirghiz or Kirgizstan or "Lao PDR" or Laos or Lebanon or Lesotho or Basutoland or Liberia or Libya or Macedonia or Madagascar or "Malagasy Republic" or Malaysia or Malaya or Malay or Sabah or Sarawak or Malawi or Mali or "Marshall Islands" or Mauritania or Mauritius or "Agalega Islands" or Mexico or Micronesia or "Middle East" or Moldova or Moldovia or Moldovian or Mongolia or Montenegro or Morocco or Ifni or Mozambique or Myanmar or Myanma or Burma or Namibia or Nepal or "Netherlands Antilles" or Nicaragua or Niger or Nigeria or Muscat or Pakistan or Palau or Palestine or Panama or Paraguay or Peru or Philippines or Philipines or Phillipines or Phillippines or "Papua New Guinea" or Romania or Rumania or Roumania or Rwanda or Ruanda or "Saint Lucia" or "St Lucia" or "Saint Vincent" or "St Vincent" or Grenadines or Samoa or "Samoan Islands" or "Navigator Island" or "Navigator Islands" or "Sao Tome" or Senegal or Serbia or Montenegro or Seychelles or "Sierra Leone" or "Sri Lanka" or "Solomon Islands" or Somalia or Sudan or Suriname or Surinam or Swaziland or "South Africa" or Syria or Tajikistan or Tadzhikistan or Tadjikistan or Tadzhik or Tanzania or Thailand or Togo or "Togolese Republic" or Tonga or Tunisia or Turkey or Turkmenistan or Turkmen or Uganda or Ukraine or Uzbekistan or Uzbek or Vanuatu or "New Hebrides" or Venezuela or Vietnam or "Viet Nam" or "West Bank" or Yemen or Zambia or Zimbabwe) not ("African-American*" or "African American*" or "Mexican American*" or "American Indian*" or "Asian American*" or "native american*"))  *Indexes=SCI-EXPANDED, SSCI, A&HCI, CPCI-S, CPCI-SSH, BKCI-S, BKCI-SSH, ESCI, CCR-EXPANDED, IC Timespan=All years* |
| # 1 | [**716,261**](https://apps-webofknowledge-com.libproxy.ucl.ac.uk/summary.do?product=WOS&doc=1&qid=1&SID=C2GljNlVrqt3Wd674te&search_mode=AdvancedSearch&update_back2search_link_param=yes) | TS=(Africa or Asia or Caribbean or "West Indies" or "Middle East" or "South America" or "Latin America" or "Central America")  *Indexes=SCI-EXPANDED, SSCI, A&HCI, CPCI-S, CPCI-SSH, BKCI-S, BKCI-SSH, ESCI, CCR-EXPANDED, IC Timespan=All years* |

**Section 4: PRISMA checklist**

| **Section and Topic** | **Item #** | **Checklist item** | **Location where item is reported** |
| --- | --- | --- | --- |
| **TITLE** | | |  |
| Title | 1 | Identify the report as a systematic review. | Page 1 |
| **ABSTRACT** | | |  |
| Abstract | 2 | See the PRISMA 2020 for Abstracts checklist. | Page 2 |
| **INTRODUCTION** | | |  |
| Rationale | 3 | Describe the rationale for the review in the context of existing knowledge. | Page 2-3 |
| Objectives | 4 | Provide an explicit statement of the objective(s) or question(s) the review addresses. | Page 3 |
| **METHODS** | | |  |
| Eligibility criteria | 5 | Specify the inclusion and exclusion criteria for the review and how studies were grouped for the syntheses. | Page 3 |
| Information sources | 6 | Specify all databases, registers, websites, organisations, reference lists and other sources searched or consulted to identify studies. Specify the date when each source was last searched or consulted. | Page 3 |
| Search strategy | 7 | Present the full search strategies for all databases, registers and websites, including any filters and limits used. | Supplementary material |
| Selection process | 8 | Specify the methods used to decide whether a study met the inclusion criteria of the review, including how many reviewers screened each record and each report retrieved, whether they worked independently, and if applicable, details of automation tools used in the process. | Page 3 |
| Data collection process | 9 | Specify the methods used to collect data from reports, including how many reviewers collected data from each report, whether they worked independently, any processes for obtaining or confirming data from study investigators, and if applicable, details of automation tools used in the process. | Page 3-4 |
| Data items | 10a | List and define all outcomes for which data were sought. Specify whether all results that were compatible with each outcome domain in each study were sought (e.g. for all measures, time points, analyses), and if not, the methods used to decide which results to collect. | Page 3 |
|  | 10b | List and define all other variables for which data were sought (e.g. participant and intervention characteristics, funding sources). Describe any assumptions made about any missing or unclear information. | Page 3 |
| Study risk of bias assessment | 11 | Specify the methods used to assess risk of bias in the included studies, including details of the tool(s) used, how many reviewers assessed each study and whether they worked independently, and if applicable, details of automation tools used in the process. | Not assessed, reported in methods |
| Effect measures | 12 | Specify for each outcome the effect measure(s) (e.g. risk ratio, mean difference) used in the synthesis or presentation of results. | Not applicable |
| Synthesis methods | 13a | Describe the processes used to decide which studies were eligible for each synthesis (e.g. tabulating the study intervention characteristics and comparing against the planned groups for each synthesis (item #5)). | Page 4 |
|  | 13b | Describe any methods required to prepare the data for presentation or synthesis, such as handling of missing summary statistics, or data conversions. | Page 4 |
|  | 13c | Describe any methods used to tabulate or visually display results of individual studies and syntheses. | Page 4 |
|  | 13d | Describe any methods used to synthesize results and provide a rationale for the choice(s). If meta-analysis was performed, describe the model(s), method(s) to identify the presence and extent of statistical heterogeneity, and software package(s) used. | Not applicable |
|  | 13e | Describe any methods used to explore possible causes of heterogeneity among study results (e.g. subgroup analysis, meta-regression). | Not applicable |
|  | 13f | Describe any sensitivity analyses conducted to assess robustness of the synthesized results. | Not applicable |
| Reporting bias assessment | 14 | Describe any methods used to assess risk of bias due to missing results in a synthesis (arising from reporting biases). | Not applicable |
| Certainty assessment | 15 | Describe any methods used to assess certainty (or confidence) in the body of evidence for an outcome. | Not applicable |
| **RESULTS** | | |  |
| Study selection | 16a | Describe the results of the search and selection process, from the number of records identified in the search to the number of studies included in the review, ideally using a flow diagram. | Page 4 |
|  | 16b | Cite studies that might appear to meet the inclusion criteria, but which were excluded, and explain why they were excluded. | Page 4 |
| Study characteristics | 17 | Cite each included study and present its characteristics. | Supplementary material |
| Risk of bias in studies | 18 | Present assessments of risk of bias for each included study. | Not applicable |
| Results of individual studies | 19 | For all outcomes, present, for each study: (a) summary statistics for each group (where appropriate) and (b) an effect estimate and its precision (e.g. confidence/credible interval), ideally using structured tables or plots. | Not applicable |
| Results of syntheses | 20a | For each synthesis, briefly summarise the characteristics and risk of bias among contributing studies. | Not applicable |
|  | 20b | Present results of all statistical syntheses conducted. If meta-analysis was done, present for each the summary estimate and its precision (e.g. confidence/credible interval) and measures of statistical heterogeneity. If comparing groups, describe the direction of the effect. | Not applicable |
|  | 20c | Present results of all investigations of possible causes of heterogeneity among study results. | Not applicable |
|  | 20d | Present results of all sensitivity analyses conducted to assess the robustness of the synthesized results. | Not applicable |
| Reporting biases | 21 | Present assessments of risk of bias due to missing results (arising from reporting biases) for each synthesis assessed. | Not applicable |
| Certainty of evidence | 22 | Present assessments of certainty (or confidence) in the body of evidence for each outcome assessed. | Not applicable |
| **DISCUSSION** | | |  |
| Discussion | 23a | Provide a general interpretation of the results in the context of other evidence. | Page 8 |
|  | 23b | Discuss any limitations of the evidence included in the review. | Page 8-9 |
|  | 23c | Discuss any limitations of the review processes used. | Page 8-9 |
|  | 23d | Discuss implications of the results for practice, policy, and future research. | Page 8 |
| **OTHER INFORMATION** | | |  |
| Registration and protocol | 24a | Provide registration information for the review, including register name and registration number, or state that the review was not registered. | Page 9 (not registered) |
|  | 24b | Indicate where the review protocol can be accessed, or state that a protocol was not prepared. | Not applicable |
|  | 24c | Describe and explain any amendments to information provided at registration or in the protocol. | Not applicable |
| Support | 25 | Describe sources of financial or non-financial support for the review, and the role of the funders or sponsors in the review. | Page 9 |
| Competing interests | 26 | Declare any competing interests of review authors. | Page 9 |
| Availability of data, code and other materials | 27 | Report which of the following are publicly available and where they can be found: template data collection forms; data extracted from included studies; data used for all analyses; analytic code; any other materials used in the review. | Page 9 |

*From:*  Page MJ, McKenzie JE, Bossuyt PM, Boutron I, Hoffmann TC, Mulrow CD, et al. The PRISMA 2020 statement: an updated guideline for reporting systematic reviews. BMJ 2021;372:n71. doi: 10.1136/bmj.n71

For more information, visit: <http://www.prisma-statement.org/>
